# What interventions are effective to prevent or respond to female genital mutilation? A review of existing evidence from 2008–2020

**DOI:** 10.1371/journal.pgph.0001855

**Published:** 2023-05-16

**Authors:** Dennis Juma Matanda, Nina Van Eekert, Melanie Croce-Galis, Jill Gay, Maria Johanna Middelburg, Karen Hardee

**Affiliations:** 1 Population Council, Nairobi, Kenya; 2 University of Antwerp, Antwerp, Belgium; 3 What Works Association, Washington DC, United States of America; 4 Right to Rise, Amsterdam, the Netherlands; UNAM: Universidad Nacional Autonoma de Mexico, MEXICO

## Abstract

As the final decade of acceleration towards zero new cases of Female Genital Mutilation (FGM, SDG Target 5.3) by 2030 has begun, increasing the rigour, relevance, and utility of research for programming, policy development and resource allocation is critical. This study aimed to synthesize and assess the quality and strength of existing evidence on interventions designed to prevent or respond to FGM between 2008 and 2020.The study drew on a Rapid Evidence Assessment of the available literature on FGM interventions. The quality of studies was assessed using the ‘How to Note: Assessing the Strength of Evidence’ guidelines published by the Foreign, Commonwealth and Development Office (FCDO) and strength of evidence using a modified Gray scale developed by the What Works Association. Of the 7698 records retrieved, 115 studies met the inclusion criteria. Of the 115 studies, 106 were of high and moderate quality and were included in the final analysis. This review shows that at the system level, legislation-related interventions must be multifaceted to be effective. Whilst all levels would benefit from more research, for the service level especially more research is needed into how the health system can effectively prevent and respond to FGM. Community-level interventions are effective for changing attitudes towards FGM, but more must be done to innovate with these interventions so that they move beyond affecting attitudes alone to creating behaviour change. At the individual level, formal education is effective in reducing FGM prevalence among girls. However, the returns of formal education in ending FGM may take many years to be realized. Interventions targeting intermediate outcomes, such as improvement in knowledge and change in attitudes and beliefs towards FGM, are equally needed at the individual level.

## Introduction

Female genital mutilation (FGM) is a practice that “involves partial or total removal of the female external genitalia or other injury to the female genital organs for non-medical reasons” [1]. The practice has no health benefits and is internationally recognized as a violation of girls’ and women’s human rights [1, 2]. Across the globe, more than 200 million girls and women alive today have undergone FGM in 31 countries with representative data on prevalence [3, 4]. While girls are one third less likely today to undergo the harmful practice than 30 years ago, rapid population growth in some of the world’s poorest countries where FGM persists threatens to roll back progress since there continue to be more girls exposed to the practice [3, 4]. Moreover, the emergence of the COVID-19 pandemic in 2020 had a huge impact on efforts towards achieving zero new cases of FGM by 2030, as envisaged in the Sustainable Development Goals (SDGs, Target 5.3) [2]. Measures to contain the spread of COVID-19, such as restrictions on movement and social distancing, have directly affected the implementation of FGM interventions. Closure of schools, along with local and national lockdowns, has led to girls spending more time at home and facing increased economic hardship, exacerbating the problem. It is estimated that 2 million additional FGM cases that would otherwise have been averted could occur over the next decade as a result of the pandemic [5]. In 2020 alone, an estimated 4.1 million girls were at risk of undergoing FGM, with the number of girls undergoing FGM each year projected to rise to 4.6 million by 2030 [4, 6]. The need to accelerate progress towards FGM elimination is therefore even more pertinent in the context of the COVID-19 pandemic. Without concerted and accelerated actions, an estimated 68 million girls are at risk of FGM by 2030 [6].

Over the last decade (2010–2020), despite intensified efforts to conduct research globally on addressing FGM, the evidence-base of rigorous high-quality studies on interventions that are effective in ending the practice remains sparce and not well known. Knowledge of what works has remained elusive, partly due to the limited synergy between evidence on ending FGM and programme and policy implementation. The disconnect between research and programming is reflected in the inadequate uptake of evidence-based FGM findings to inform policy and programmes and to support mobilization of resources to end the practice. Moreover, much of the research conducted to date has not adequately engaged affected communities, key stakeholders (e.g., including researchers, programme implementers and policymakers), from the outset. Other reasons for the limited uptake of evidence include inadequate communication of evidence-based findings and insufficient support or budgets to utilize and operationalize research findings. In addition, inadequate monitoring and evaluation indicators and a lack of coordination between programme personnel and research practitioners in the sector has posed challenges for determining the effectiveness of FGM programmes, while also resulting in research agendas that are not well-aligned with programme needs [7]. Yet, as the final decade of acceleration towards zero new cases of FGM by 2030 begins, increasing the rigour, relevance, and utility of research for programming, policy development and resource allocation is critical.

A small number of publications have collated evidence on the effectiveness of interventions to end FGM, either through systematic or non-systematic reviews [8–15]. Some of these reviews have only included studies that used experimental and quasi-experimental designs to determine the effectiveness of interventions to prevent FGM. Due to the limited number of such studies in the FGM field, most of these reviews have reported limited evidence [11, 12]. In their 2013 review aiming to identify what works and what does not in ending the practice, Johansen and colleagues described the merits and demerits of common approaches applied to end FGM, although they did not assess the quality of the evidence [8]. In 2011, in a non-systematic review of FGM programmatic interventions, the WHO focused mostly on Africa in highlighting successful approaches [15]. In a rapid evidence assessment, Esho and colleagues synthesized high quality studies investigating interventions to support FGM abandonment between January 2000 and August 2016 [16].

The current review was designed to contribute to SDG target 5.3 of zero new cases of FGM by 2030 through strengthening the synergy between evidence generation and FGM programmes, as well as to inform a global research agenda for FGM [17, 18]. In contrast to previous work, the current review considered qualitative, quantitative and mixed methods studies; included literature in three languages; and used a novel methodology to assess both the quality of studies and the strength of the evidence to inform the discourse on what works to end FGM and to guide programming. This study aimed to achieve the following objectives: (i) assess the quality of studies that have evaluated interventions for the prevention of, or response to, FGM; and (ii) describe the FGM interventions evaluated by studies deemed to be of moderate or high-quality.

## Methods

This review included available literature on FGM interventions from 2008–2020 [S1 Text]. The evidence was assessed from two dimensions. First, what was the quality of the studies that have evaluated interventions for the prevention of, or response to, FGM. Second, what was the strength of evidence of FGM interventions evaluated by studies deemed to be of moderate or high-quality. Interventions for which the evidence is of sufficient quality and strength are described, with interventions that are most promising for FGM prevention or response, based on studies of sufficient quality and strength of evidence, highlighted.

### Literature review

A Rapid Evidence Assessment (REA) approach was used to conduct a comprehensive review of the available literature on FGM interventions (S1 Table, S1 Checklist). The REA is a methodology for locating, appraising, and synthesizing evidence within a short period of time, and is primarily driven by the need to provide timely, rigorous reviews to support evidence-based recommendations [19]. For purposes of this review, ‘FGM interventions’ were defined as any form of action or process of intervening, or a deliberate process to interfere with, modify or change people’s (both women’s and men’s) thoughts, feelings, knowledge or behaviours to reduce the prevalence of FGM, or lead to the abandonment of FGM, or to offer care and other services to girls, women, and those indirectly affected by the practice (including men). The starting date of 1 January 2008 for the literature review coincided with the year that the UNFPA–UNICEF Joint Programme on the Elimination of Female Genital Mutilation: Accelerating Change was established, heralding increased global attention to FGM in terms of investment in both research and programming. The end date for the literature review was 31 August 2020. As with a systematic review, the number of studies included in the REA was determined by the search strategy [20].

The inclusion criteria for the literature search were established a priori: the search included studies focussing on assessing the effectiveness of interventions in ending FGM published in Arabic, English or French, as either evaluation reports, peer-reviewed articles or student theses, regardless of location in the world. Moreover, in order to enable assessment of quality, studies needed to have clear methodologies.

The key words used in the literature search were: (“female genital mutilation” OR “female genital cutting” OR “female genital mutilation/cutting” OR “FGM” OR “FGC” OR “FGM/C” OR “female circumcision” OR “FGM gash” OR “female genital distortion” “female sexual mutilation” OR “clitoridectomy”) AND “interventions”. The systematic search of literature was conducted in the following scientific databases: EBSCO (social sciences database, CHW Wilson, gender studies database, MEDLINE, CINAHL Plus and ERIC), JSTOR, Knowledge Commons, PubMed, SAGE journals, Web of Science and WILEY. Websites of institutions or organizations that have been involved in FGM work (n = 45) were purposively identified, according to prior knowledge of their work by the co-authors. We also used references in reports and other literature to identify institutions or organizations involved in FGM work. Additional literature was identified by searching references of retrieved studies and via suggestions from experts in the FGM field.

Literature that met the inclusion criteria was categorized as either a primary or secondary study. Primary studies refer to research that observes a phenomenon first-hand, collects, analyses and/or presents raw data, while secondary studies refer to research that summarizes and interrogates primary study data and findings.

### Assessing the quality of included studies

The quality of primary studies was assessed based on the United Kingdom Department for International Development (DfID) ‘How To Note’ guidelines [21]. Scores (0–2) were given to indicators aligning with six principles of quality: conceptual framing, transparency, appropriateness, cultural/context sensitive, validity and reliability (cfr. Table 1). A score of 2 indicated that the study adhered to all the quality measurement indicators; 1 indicated that the study adhered to at least of the quality measurement indicators, i.e., one question received a ‘Yes’ response, and the other received a ‘No’; and 0 indicated that the study did not adhere to any of the quality measurement indicators. The scores for each principle of quality were then summed, assuming equal weighting for each principle. A primary study with an aggregate score of 0–4 was considered of low quality; a study with a score of 5–8 was considered moderate quality, and a study with a score of 9–12 was considered high quality.

**Table 1:** Six principles of quality for primary and secondary studies and aligning indicators.

Principles of quality	Indicators: Does the study…?
**PRIMARY STUDIES**	
**Conceptual framing**	acknowledge existing research?
	pose a research question or outline a hypothesis?
**Transparency**	present or link to the raw data it analyses?
	declare sources of support/funding?
**Appropriateness**	identify a research design, methods, and analysis approach that is appropriate?
	demonstrate why the chosen design and method are well suited to the research question?
**Cultural/context sensitivity**	specify the geography/context in which it was conducted?
	explicitly consider any context‐specific cultural factors that may bias the analysis/findings?
**Validity**	minimize the possibility that some confounding or unseen variable is affecting any changes observed?
	specify how representative the sample used is?
**Reliability**	specify the measures that were put in place to ensure consistency of data collection?
	specify the extent to which the measures used are internally reliable?
**SECONDARY STUDIES**	
**Transparency**	describe where and how studies were selected for inclusion?
**Validity**	assess/consider the quality of the studies included?
**Reliability**	draw appropriate conclusions based on the reviews conducted?

The quality of secondary studies was assessed based on scores (0–2) given to indicators aligning with 3 principles of quality: transparency, validity and reliability (cfr. Table 1). A score of 2 indicated no limitations identified in relation to the indicator, a score of 1 denoted a lack of clarity and a score of 0 was assigned if a study did not address the principle being assessed. The score ranges for assessing the quality of secondary studies were: 5–6 (high quality), 3–4 (moderate quality), and 0–2 (low quality).

### Assessing the strength of evidence of the included studies

Data from studies that were graded as being of moderate or high quality were extracted using a pre-designed data extraction form, with columns for author/s name, the study title, year of publication, the study design, intervention description, study outcomes, reasons for success or failure of the intervention and study conclusions (S2 Table). Due to the heterogeneity in study designs, we carried out a thematic analysis focusing on the main themes (interventions) that were evident in the studies. We used a narrative synthesis: an approach that synthesises findings from multiple studies relying primarily on the use of words and text, to summarize and explain the findings on the effectiveness of interventions and the reasons given for their effectiveness, or lack thereof.

The strength of the evidence in these studies was evaluated using a modified Gray scale [22–24]. Originally developed with five levels of evidence to assess public health programming, the Gray scale was subsequently modified by Gay and colleagues [22] to sub-divide the level three classification into studies and evaluations whose designs include control groups (IIIa) and those that do not (IIIb) [22] (cfr. Table 2).

**Table 2:** Modified Gray scale of the strength of evidence of individual studies.

Type	Strength of evidence
**I**	Systematic review of multiple well-designed, randomized controlled trials
**II**	Well-designed, randomized controlled trial of sufficient size
**IIIa**	Well-designed trial/study without randomization that includes a control group (e.g., quasi-experimental, matched case-control studies, pre-post with control group)
**IIIb**	Well-designed trial/study without randomization that does not include a control group (e.g., single group pre-post, cohort, time series/interrupted time series, repeated cross-sectional studies)
**IV**	Well-designed, non-experimental study from more than one centre or research group, qualitative studies, and/or analysis of routine data
**V**	Opinions of respected authorities, based on clinical evidence, descriptive studies, or reports of expert committees.

To assess the strength of a body of evidence, criteria are set for the number of studies, the number of countries and the Gray scale ratings for each study. Interventions are described as 1) successful if supporting evidence includes four or more studies that are Gray IIIb or higher and have evidence of more than one country; as 2) promising when evidence includes three or fewer studies of any Gray level or two or more studies at Gray IIIb or higher but only from one country; and 3) as not working if four or more studies that are Gray IIIb or higher and have evidence from more than one country that the intervention does not work.

### Quality assurance

Quality assurance measures were applied at every step of the review, by guaranteeing researcher triangulation and consequently reducing bias in each step of the REA process: from screening studies to determine whether they met the inclusion criteria, over quality and strength assessment. A digital referencing tool, Zotero, was used to manage all bibliographic references. To screen studies, the Covidence online platform was used as it facilitates indication of duplicate studies and facilitates a team of reviewers to concurrently screen studies. In case conflicts arose on whether the study should be included or not, a third reviewer was involved in solving the conflict. When discrepancies in scores concerning quality and strength of the studies were detected through systematically organized comparison, a discussion was held to resolve these discrepancies and reach agreement on the quality and/or strength score.

## Results

**Fig 1:**
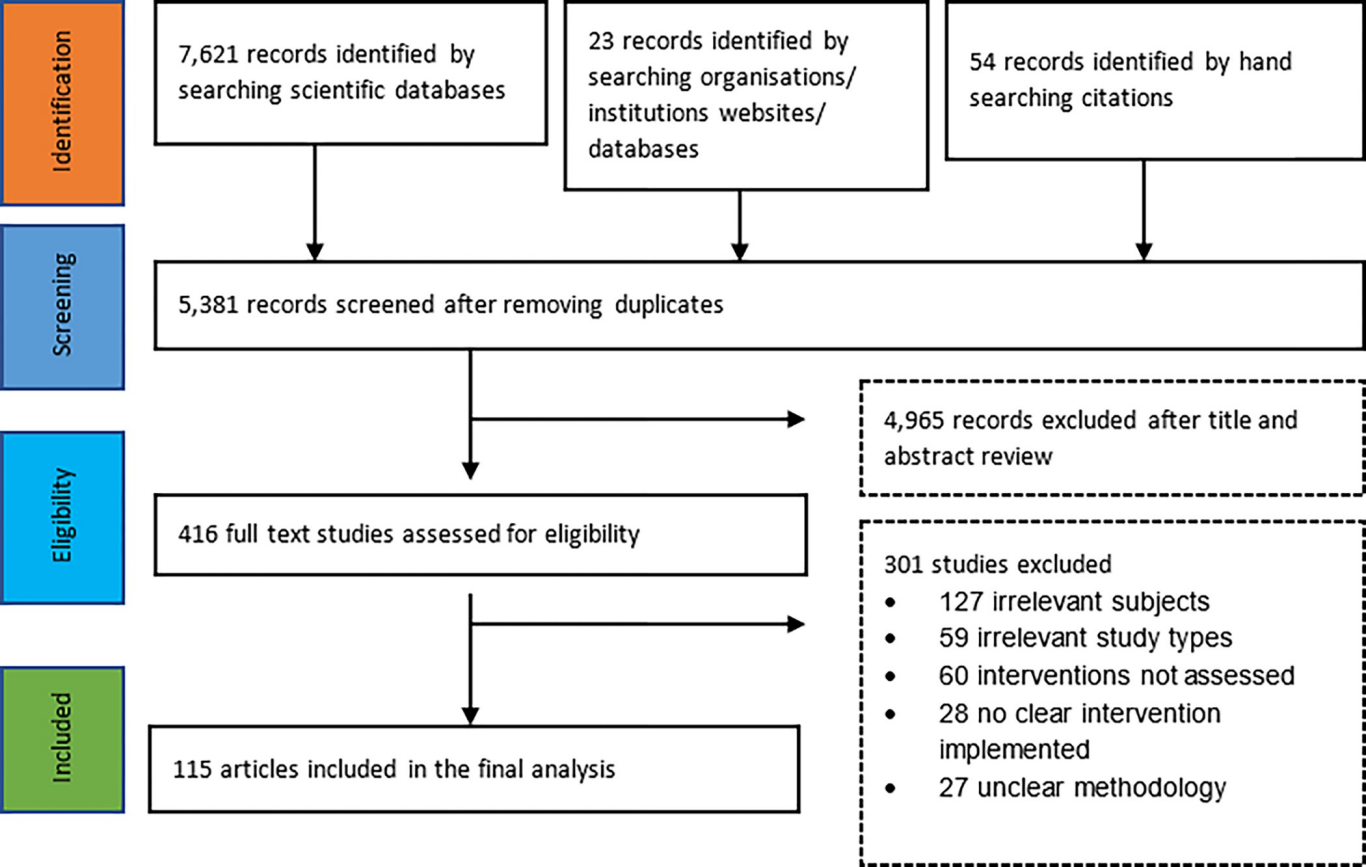
Prisma flow chart.

Of 7,698 records retrieved, 115 studies met the inclusion criteria (see Fig 1). In line with the inclusion criteria, the included studies were published between 2008 and 2020, with the highest number of studies published in 2019 and 2020 (n:26). Geographically, the studies were mostly carried out in Africa (n:82), 15 were based in Europe; and 3 were conducted in Asia. The remaining studies were inter-regional. Notably, none of the studies published in Arabic met the inclusion criteria, while the studies published in French were duplicates of studies published in English. Most studies were either of high quality (n:52) or moderate quality (n:54). Only 9 studies were considered low quality.

The UNFPA–UNICEF Joint Programme on the Elimination of Female Genital Mutilation: Accelerating Change global theory of change and the compendium of indicators for measuring the effectiveness of FGM interventions were drawn on to classify the various intervention approaches [25, 26]. Both the global theory of change and the compendium of indicators embrace a holistic and multisectoral approach to ending FGM. Approaches are categorized as either system level, community level, individual level, or service level (see Table 3) [25, 26]. Studies that involved several strategies and activities were classified based on the main intervention that was assessed. Most studies that were considered moderate and high quality were conducted at community level (41%), followed by system level (28%), individual level (20%) and service level (10%).

**Table 3:** Classification and strength of various intervention approaches.

Types of FGM interventions by Level	N total	N HQ	N MQ	Strength of evidence
**SYSTEM LEVEL**				
Legislative interventions	30	17	13	Promising when combined and with consideration for local context
**SERVICE LEVEL**				
Training health-care providers/capacity building of the health care system	6	4	2	Successful
Rescue centers	5	2	3	Ineffective on its own
**COMMUNITY LEVEL**				
Health education	9	2	7	Successful
Community engagement approaches	11	3	6	Successful
Social marketing and media efforts	18	8	11	Successful
Use of religious/cultural leaders	7	5	2	Successful
Public statements/declarations	4	2	2	Promising
Conversion of traditional practitioners	5	2	3	Ineffective
**INDIVIDUAL LEVEL**				
Formal education for girls	13	7	6	Successful
Alternative rites of passage	13	4	6	Ineffective on its own

N = number of studies; HQ = high quality studies; MQ = medium quality studies

The strength of the evidence was assessed for all interventions included in moderate and high-quality studies. Combining the Gray rating of the moderate and high-quality studies with the geographical spread of the interventions allowed for analysis of successful programming towards abandoning FGM. Using the criteria described above for interventions that are successful, promising, and not working, Table 3 shows that six successful interventions with supporting evidence were identified: one at individual level, four at community level and one at service level. Two identified interventions, one at the community and one at the system level, were shown to be promising; and four identified interventions, with one at each level, were considered not to be effective.

### System level: Enabling environment for ending FGM

System level interventions include those implemented at a macro level to provide an enabling environment for ending FGM. The system-level studies included in this review focused on assessing the effectiveness of national anti-FGM legislation in ending the practice.

#### Legislative interventions

Legislation can provide platforms and avenues upon which other interventions can be safely implemented. Legislation may also serve to accelerate change in FGM practice in environments where community members are already questioning or have abandoned the practice and are seeking social acceptance. Nevertheless, evidence suggests that legislation alone is not effective in changing attitudes towards FGM and its prevalence [27–32]. Previous research shows that legislation must be accompanied with political will [9, 33, 34], the existence of locally appropriate enforcement mechanisms [35], a combination of other interventions that are acceptable to the target community, sufficient resources for implementation [36] and sensitization [37] to show impact in knowledge, attitudes and norms driving the practice [9, 33–41]. Legislation can also be useful in an environment where it will be applied across a geographical jurisdiction and leaves little room for misinterpretation [9, 38]. However, in most countries, legislation may take a long time to end FGM as countries usually pass laws as a precursor to enforcement, with no effective mechanisms in place to report, refer, and protect girls and women at risk of FGM [27–32, 37, 38, 40, 42–44]. Consequently, the number of court cases are usually low or non-existent, suggesting a lack of political will and readiness of the community to abandon the practice [34]. Notably, some practising communities do not conduct FGM mainly due to fear of the legal consequences of disobedience [43].

Legislation enacted without consideration of the local context may be counterproductive and even harmful for intended beneficiaries [45, 46], as it can lead to transformation rather than the elimination of the practice, such as: increased medicalization and/or changes in the type of cut, cutting at younger ages, and/or the practice continuing in secret [33, 43, 46–54]. Moreover, the enforcement of legislation has, in some cases and especially in developed countries, alienated the intended beneficiaries, as it reduced the number of beneficiaries seeking care such as reproductive health services [55], and may even have compromised reporting [49].

### Service level: Services for FGM prevention, protection and care

Service-level interventions aim to protect girls and women at risk of FGM, prevent FGM, and provide care to women and girls who have undergone FGM. Interventions assessed for effectiveness in this review include training health-care providers and capacity-building of the health system, and the use of rescue centres.

#### Training health-care providers/capacity-building of the health-care system

Most of the interventions focused on imparting knowledge and skills to health-care providers, either to act as agents of change in the prevention of FGM or to offer better services to clients seeking health services post-FGM. Evidence shows that training can be effective in imparting knowledge to health-care workers [56, 57]; enhance their skills in managing complications related to FGM in health-care settings [58] as well as evaluating the risk of exposure to FGM [59]; and might change their attitudes towards FGM [56] or even be critical for rejecting/preventing the practice [56, 60]. However, in contrast, other systematic reviews suggested that training health personnel did not change their knowledge and beliefs/attitudes regarding FGM and fewer providers wished to play a role in educating clients about the practice and a sense of advocacy among participating health personnel appeared weak [10, 11]. Although there is limited evidence on the effectiveness of interventions at the service level, available evidence shows that training health-care providers can improve their knowledge and skills to act as agents of change in the prevention of FGM and offer quality services to clients seeking care post-FGM [56, 58]. An improved health-care system should therefore have the capacity to manage and provide optimal services to clients who have undergone FGM and prevent the practice from occurring among those at risk [61].

#### Rescue centres

Rescue centres or safe houses aim to provide protection and refuge for girls who are at risk of FGM during the cutting period. Notably, most of the studies included rescue centres among other interventions, with limited information on the assessment of rescue centres as an independent intervention. Apart from providing shelter to girls running away from FGM, rescue centres also educate girls on the health risks and illegality of FGM, and its violation of human rights [42, 62]. Rescue centres face challenges such as limited resources and lack of recognition and buy-in of the intervention by the community and there is therefore limited evidence on their effectiveness [50]. The few available studies show that rescue centres can provide short-term refuge for girls at risk of FGM [42] and can be successful if integrated with other interventions to eradicate FGM [50]. However, rescue centres are limited in providing long-term solutions to ending the practice [14, 39, 50].

### Community level: Addressing gender inequalities and social norms

This category included interventions implemented in communities with the aim of challenging existing gender inequalities and social norms associated with FGM. Specific interventions assessed for effectiveness at the community level were health education, comprehensive community engagement, media/social marketing campaigns/communication initiatives, public declarations/statements, working with religious/cultural leaders, and conversion of traditional practitioners.

#### Health education

Health education interventions were mostly educational campaigns for awareness creation targeted at community members. Health education using the health risk approach can be useful in imparting knowledge related to the physical, psychological, and emotional consequences of FGM [63, 64]. Health education can also trigger and guide discussions among practising communities on the effects of FGM and hence the need for action to eliminate the practice [65]. Several studies found that health education had a positive impact on changing knowledge, beliefs, and the attitude of individuals towards the practice [15, 63, 66–71].

A systematic review of educational sessions showed that the effects of health education across communities and target groups may not be uniform [11]. Previous research suggests that health education can be more effective in an environment where context is considered, especially to reduce resistance from the target group [72, 73]. Besides, it suggests that while health education may be effective in changing knowledge, attitudes and beliefs, an additional intervention may be needed to influence behaviour change [15].

#### Community engagement approaches

Community engagement approaches create a platform for internal community discussions on harmful traditional practices such as FGM. Most of the studies focused on community dialogues or conversations where participants were expected to begin to question the role of some practices that are proven to have negative consequences, and therefore facilitate change. A systematic review of interventions to end FGM found that an underlying change mechanism was that providing information about FGM to communities, including men, would increase knowledge and change attitudes [11, 74–78]. Yet, this change is not always translated to FGM abandonment [74, 79–81], and might sometimes lead to medicalization of the practice and a change in the type of FGM [82]. The evidence suggested that the key to changing FGM-related behaviour was how the information was disseminated [11].

Community engagement approaches that use a holistic approach and seek to empower community members have been effective in changing attitudes towards FGM and in some cases changing behaviour [65, 79]. It is shown that targeting FGM is most effective and well-received when a broader approach, targeting multiple sectors is used, and the community is supported to resolve other challenges that are not necessarily related to FGM [8, 83]. Evidence thus also shows that the use of tailored, contextually appropriate and locally generated interventions where the community is fully engaged can yield positive results in changing behaviour. A good example is the use of the community education and empowerment programmes (CEEP) which enabled communities to be engaged in the design of the interventions and receive formal and informal education through four modules that included human rights, problem-solving, basic hygiene, and women’s health [8, 84].

#### Social marketing and media efforts

Research has shown that social marketing and media efforts are effective in changing social norms and attitudes towards abandoning FGM and in some cases, reducing the practice [11, 26, 36, 50, 83, 85–94]. Mainstream newspapers, television reports, SMS messaging, social media, theatre productions, television and radio melodramas can all shape conversations about FGM and accelerate the shift in social norms towards FGM abandonment [26]. Positive shifts in attitudes can be achieved when considerable knowledge of risks is disseminated, enabling the target population to become a source of information and agent of change [87]. Effective sensitization campaigns have empowered girls to refuse FGM and to report to relevant authorities when at risk [50]. There also appears to be a substantially increased awareness not just of the illegality of FGM, but of the consequences of breaking the law [83] and of the negative impacts of infibulations [36]. Sensitization and training have also resulted in increased awareness and knowledge of the consequences and dangers of FGM, thereby contributing to disapproval and abandonment of the practice [11, 26, 85, 88, 92]. The UNFPA–UNICEF Joint Programme argues that young people can use social media to raise awareness about FGM and gender equality, as the promotion of FGM abandonment across social networks has given communities an opportunity to see the possibility of rapid, widespread change [95].

However, evidence shows that interventions which only supply information, education and campaigns to increase FGM awareness are not sufficient to change behaviour 15], and even might lead to medicalization of the practice [14]. FGM is an entrenched generational practice and eradicating it in a community requires concerted efforts over an extended period. Advocacy and awareness-raising efforts that take a holistic multisectoral approach constitute best practices that should be sustained in order to maintain their impact for future generations [96].

#### Use of religious/cultural leaders

As FGM is often associated with religious and cultural obligations, changing religion and culture to support abandonment of FGM is therefore a viable approach to contributing towards elimination of FGM [14]. Religious and cultural leaders can be at the forefront of questioning the religious underpinnings of the practice and in publicly declaring opposition to the practice [97, 98]. In many places where FGM is practiced, traditional and religious leaders sometimes wield more power and influence than the government [38]. Religious and cultural leaders can effectively pass on messages to the community, especially in communities that are ready for change, and play an important role in the elimination of FGM at the community level [14, 82, 99, 100]. Islamic religious leaders have previously issued edicts (Fatwa) against FGM [8, 96]. While the independent effects of such edicts are yet to be quantified, such strong positions may have some effect on behaviour and practices in communities that accord high levels of respect to religious leaders. Conversely, reluctance by religious leaders can hinder progress towards elimination of FGM [36, 38].

#### Public declarations/statements

Public declarations of FGM abandonment by community, religious and political leaders or other influential people in a community are critical, as they may signal a commitment and readiness to abandon the practice [26, 95, 99, 101]. Mass and social media and other forms of communication have played a central role in amplifying these public declarations and in encouraging other communities to abandon FGM. The review further found that public declarations, when supported by post-declaration follow-up and support, were highly effective in preventing further cases of FGM. Regular and repeated awareness-raising interventions addressed at all sections of the society and that stress the detrimental effects of FGM, in addition to encouraging communities to make a declaration of abandonment, may be a first step towards changing attitudes and practices among community members in FGM-prevalent settings [99].

#### Conversion of traditional practitioners

Intervention activities involved either working with former traditional practitioners who had abandoned performing FGM or providing an alternative income to active traditional practitioners to encourage them to stop carrying out FGM. Evidence shows that there have been efforts to convert and provide traditional practitioners with alternative sources of income, but such efforts have been largely ineffective [8, 33, 39, 50, 102]. In most cases, these efforts resulted in the practicing communities changing the location where the practice used to occur and the practitioner for FGM [33], increased medicalization of FGM [102] and/or secrecy in conducting the practice [39, 50]. Efforts to provide traditional practitioners with an alternative income have been largely ineffective and may need to be recalibrated if they are to be optimally used in the prevention of FGM at the community level [8].

### Individual level: Empowering girls and women

Interventions at the individual level include those that aim to empower girls and women to make their own informed decisions regarding their sexual and reproductive rights. Interventions assessed for effectiveness under this category were formal education for women in terms of educational attainment, and approaches that used alternative rites of passage to encourage abandonment.

#### Formal education for girls

Evidence shows that formal education is effective in reducing FGM prevalence [103, 104]. Formal education exposes girls to new information, including the health risks/consequences and illegality of FGM and can therefore play a significant role in the abandonment of the practice by empowering women and girls to demand their rights and to challenge existing gender and social inequalities such as FGM [10, 11, 42, 50, 54, 62, 105–107]. As well, studies have linked low levels of education to an increased likelihood of supporting and/or practising FGM with evidence suggesting that FGM was highly prevalent among girls who did not pursue formal education compared with those in school [103]. If a mother has higher education, her daughter is less likely to undergo FGM [50, 108, 109]. However, other studies did not find a relation between educational attainment and attitude towards FGM [32, 110].

#### Alternative rites of passage

Alternative rites of passage allow girls to undergo training and to graduate to womanhood without being subjected to FGM [42]. During this process, girls are also educated on different topics, such as human rights and the adverse effects of FGM, and are encouraged to abandon the practice [50]. Among groups that had dedicated training on the harmful effects of FGM, alternative rites of passage increased reproductive health knowledge. In some instances, while the training did not necessarily lead to the abandonment of FGM [111, 112], attitudes towards the practice might have changed [113, 114] as well as changes in the practice were reported–e.g. from severe to less severe cuts [111].

Alternative rites of passage facilitate community ownership and support, as they maintain key cultural practices, increase knowledge and empowerment of girls, and increase publicity about change through community celebrations [111]. However, this approach is only viable in communities where FGM is part of a rite of passage [8], and therefore its impact is limited [79]. Alternative rites of passage adopted in isolation does not address the underlying social values associated with FGM and therefore, the social stigma of not undergoing FGM remains and girls continue to be pressured to undergo the practice [79]. In this line, the success of alternative rites of passage has been curtailed by a trend in some communities where FGM is increasingly being performed at younger ages and with less ceremony and ritual. Thus, while some components of alternative rites of passage, such as educational programmes, can be effective in changing attitudes [54, 113], the risk of exclusion, perceived loss of cultural identity, changing meanings ascribed to cultural practices, lack of precise knowledge about subjective sexual experience and negative stereotyping limit the success of such programmes [115]. For alternative rites of passage to be effective, especially in dealing with the issue of stigma, it should be implemented in combination with other intervention approaches. This includes community awareness-raising initiatives, intensive community mobilization and sensitization about FGM combined with a public declaration ceremony which is fully integrated into a girls’ empowerment program [111, 116].

## Discussion

The review on which this paper is based has analysed the quality and strength of evidence for interventions to contribute to reaching SDG target 5.3 of zero new cases of FGM by 2030 [17]. Most of the studies reviewed consistently advocated for the implementation of holistic and multisectoral interventions to end FGM. The multisectoral approach envisages a scenario where: laws and policies are in place and enacted, and budgets and coordinated systems are in place; community members, including men and boys and religious leaders, deliberate new norms and are equipped with the skills to motivate others to abandon FGM; girls and women are empowered to defend their rights and access education, social, health and legal services; and FGM is mainstreamed in social development and services for women and girls.

While the holistic and multisectoral approach is based on existing evidence on the pathways towards change, it is acknowledged that behaviour change processes are not linear; pathways of change are sometimes interdependent or cross-cutting [117–119]. Synergies across the various levels (system, community, individual and service) are expected to enable FGM elimination and the advancement of gender equality. Additionally, it may not be practically possible for a programme to intervene fully in all the suggested domains. Instead, the scale of an FGM programme, existing partners and the local context should be considered in determining the most strategic combination of interventions [25, 26].

## Limitations

Whilst the strength of this review is evident, several limitations must be noted. First, this review is limited in its ability to reach a strong conclusion on the extent to which the assessed interventions led to a reduction in FGM prevalence, as most of the outcomes are related to knowledge and attitudinal changes. Moreover, causality was not ascertained as part of this review, as most of the interventions assessed did not use rigorous study designs for determining effects of interventions on intended outcomes. Second, most of the interventions reviewed had a short implementation span which may have been insufficient to observe changes in the practice. Equally importantly, this review only assessed evidence from existing evaluations, yet there may be many promising programmes being implemented that have not been evaluated and require further research. Third, measuring social change range from the description of how change occurs during and after implementation of the various interventions, to the measurement of changes in FGM practice or attitudes. This is challenging as it requires standardized indicators that can be compared over time and across settings. Fourth, the narrative synthesis approach combined with the Gray rating of the moderate and high-quality studies with the geographical spread of the interventions made it possible to analyse successful, promising and interventions with limited evidence on the effectiveness in ending FGM. Nonetheless, given the heterogeneity in study designs and the limited evidence across countries and regions overall, it is difficult to make strong claims about interventions that may be said to ‘work,’ particularly in varying cultural contexts.

Overall, this review identified a general gap in monitoring, evaluation, and learning across the various interventions. Most of the monitoring and evaluation frameworks tended to focus more on process and intermediate outcomes, and less on demonstrating if interventions were effective in reducing FGM prevalence. This highlights the need for the application of robust study designs and analytical techniques that can clearly demonstrate the effectiveness (cause and effect) of FGM interventions on FGM abandonment, as well as the complementarity of each intervention in complex interventions.

## Conclusion

By assessing the quality and strength of evidence, this study has identified successful and promising interventions, as well as interventions with inadequate evidence on their effectiveness, and thus offers suggestions for guiding potential programming and policymaking. Firstly, health education, community dialogues with parents and religious leaders, the use of media and social marketing efforts, and formal education for women and girls are examples of interventions that have a strong enough body of evidence to justify wider implementation as part of comprehensive efforts to eliminate FGM. Secondly, legislation accompanied by political will in combination with additional interventions, creating FGM-free communities through public declarations, and training health provider are promising interventions with further evidence needed. Thirdly, providing traditional practitioners with alternative sources of income and alternative rites of passage with a focus on public ceremonial passage of girls is not effective in ending FGM. Lastly, adequately addressing FGM requires a holistic approach bringing together interventions that are sensitive to the complexity of FGM.

## Supporting information

S1 Checklist The PRISMA 2020 that comprises a 27-item checklist addressing the introduction, methods, results and discussion sections of a systematic review report. (DOCX)

S1 Text Main study report from which the current study was based on. (PDF)

S1 Table Rapid Evidence Assessment (REA) checklist. (DOCX)

S2 Table Summary of studies that were considered to be of moderate or high quality and included in the final analysis. (DOCX)
