# Correction: What interventions are effective to prevent or respond to female genital mutilation? A review of existing evidence from 2008–2020

**DOI:** 10.1371/journal.pgph.0004141

**Published:** 2024-12-31

**Authors:** Dennis Juma Matanda, Nina Van Eekert, Melanie Croce-Galis, Jill Gay, Maria Johanna Middelburg, Karen Hardee

The affiliation for the second author is not indicated. Nina Van Eekert is also affiliated with: The Research Foundation—Flanders, grant number 12g3322N, Brussels, Belgium.
