# Supplementary material for: What interventions are effective to prevent or respond to female genital mutilation? A review of existing evidence from 2008–2020
Source: PLOS Glob Public Health. 2023 May 16;3(5):e0001855. doi: 10.1371/journal.pgph.0001855 (PMC10187928; doi:10.1371/journal.pgph.0001855)
Supplement: S1 Text — (PDF) [file pgph.0001855.s002.pdf]

Population Council
Knowledge Commons

6-1-2021

Effectiveness of interventions designed to prevent or respond to female genital mutilation: A review of evidence

Dennis Matanda
Population Council

Melanie Croce-Galis

Jill Gay

Karen Hardee

Follow this and additional works at: https://knowledgecommons.popcouncil.org/departments_sbsr-rh

Part of the Family, Life Course, and Society Commons, and the International Public Health Commons

How does access to this work benefit you? Let us know!

Recommended Citation
Matanda, Dennis, Melanie Croce-Galis, Jill Gay, and Karen Hardee. 2021. "Effectiveness of interventions designed to prevent or respond to female genital mutilation: A review of evidence." Nairobi: UNFPA, UNICEF, WHO, and Population Council Kenya.

This Report is brought to you for free and open access by the Population Council.

Effectiveness of Interventions Designed to Prevent or Respond to Female Genital Mutilation

A Review of Evidence

June 2021

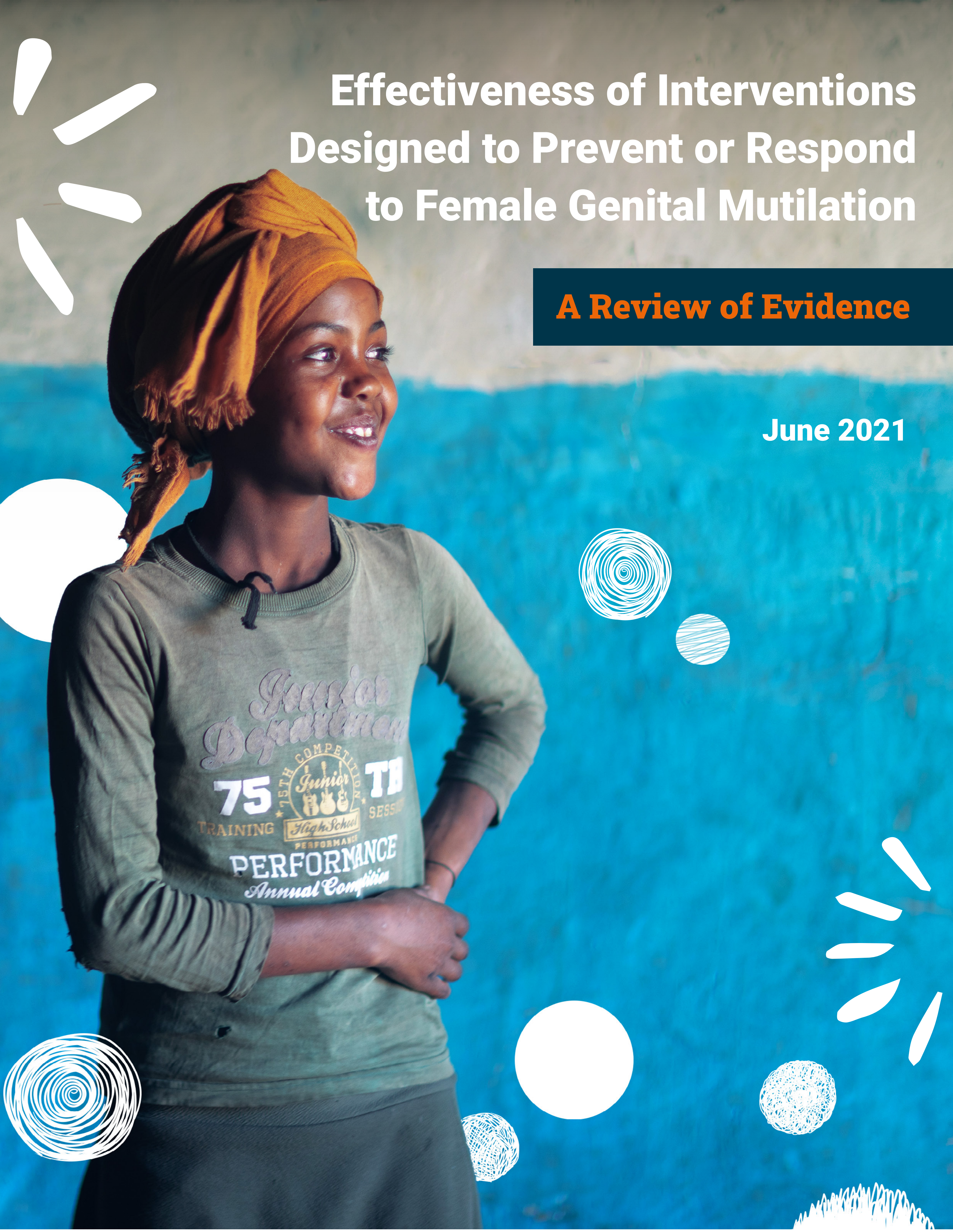

UNFPA
unicef for every child
World Health Organization
POPULATION COUNCIL KENYA
Ideas. Evidence. Impact.

Effectiveness of Interventions Designed to Prevent or Respond to Female Genital Mutilation

A Review of Evidence

Dennis Matanda
Population Council, Kenya

Melanie Groce-Galis
What Works Association

Jill Gay
What Works Association

Karen Hardee
What Works Association

Matanda Dennis, Groce-Galis Melanie, Gay Jill & Hardee Karen (2021). Effectiveness of Interventions Designed to Prevent or Respond to Female Genital Mutilation: A Review of Evidence. UNFPA, UNICEF, WHO and Population Council, Kenya.

TABLE OF CONTENTS

ACKNOWLEDGEMENTS 5

LIST OF ACRONYMS 7

EXECUTIVE SUMMARY 8

Background
Methods
Results
Conclusions

BACKGROUND 11

Introduction
Study objectives

METHODS 16

Study design
Definition of FGM interventions
Search strategy
Classification of the literature
Assessment of study quality
Describing the quality of the research
Assessing the strength of the research
Quality assurance
Evidence analysis and presentation

RESULTS 26

Description of studies assessed
Study identification, screening and eligibility assessment
Timeline of publications
Geographic location of publications
Type of publications retrieved
Quality of studies, research type and design
Classification of FGM interventions
Types of FGM intervention: moderate and high-quality studies
A. System level: Enabling environment for ending
Legislative interventions
B. Community level: Addressing gender inequalities and social norms
Health education
Community engagement approaches
Media/social marketing campaigns/ communication
Public declaration/statements
Religious/cultural leaders
Conversion of traditional practitioners
C. Individual level: Empowering girls and women
Formal education
Alternative rites of passage
D. Service level: Services for FGM prevention, protection and care
Training health-care providers/ capacity-building of the health-care system
Rescue centres
What works in FGM prevention and response?
Successful interventions with supporting evidence
Promising interventions that need further evidence
Interventions lacking evidence

DISCUSSION 66

System level: Enabling environment for ending FGM
Community level: Addressing gender inequalities and social norms
Individual level: Empowering girls and women
Service level: Services for FGM prevention, protection and care
A holistic and multisectoral approach to FGM interventions
Areas for further research
Gaps and study limitations

CONCLUSION 75

ENDNOTES 77

APPENDIX 81

Appendix 1: Summary of moderateand high-quality studies
Appendix 2: Areas for further research

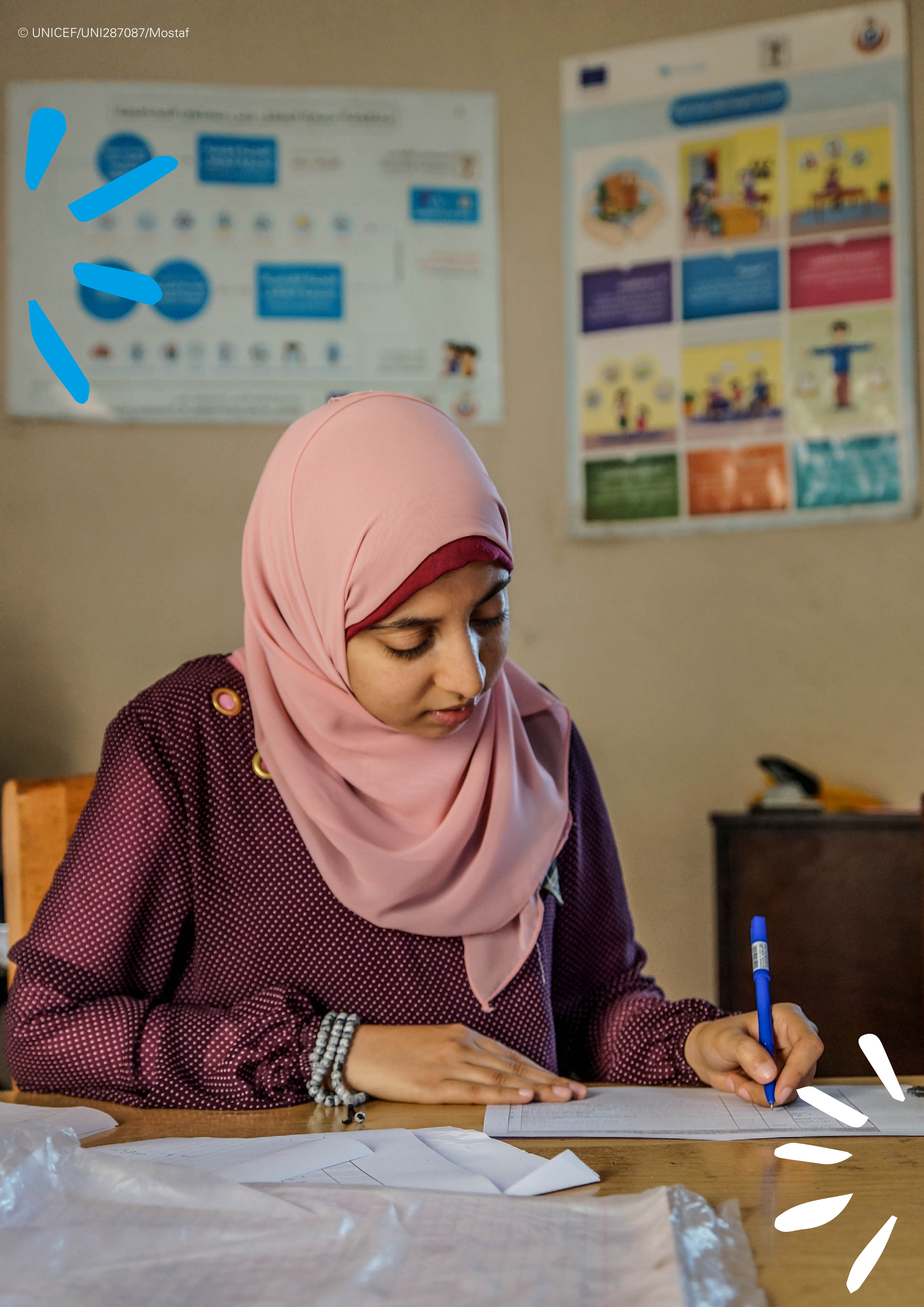
© UNICEF/UNI287087/Mostaf

ACKNOWLEDGEMENTS

We are grateful for the insightful comments and reviews by members of the reference group that enriched this report. Members of the reference group included: Nankali Maksud, Claudia Cappa, Harriet Akullu, Colleen Murray, Zahrah Nesbitt-Ahmed, Stephanie Baric, and Yasmine Sinkhada from UNICEF; Nafissatou J. Diop, Mireille Tushiminina, Berhanu Legesse, and Thierno Diouf from UNFPA; Christina Pallitto and Wisal Ahmed from WHO. We are thankful to Jacinta Muteshi for her invaluable reviews and thoughts. Many thanks to the research assistants: Erick Waga, Daniel Lango, Rachael Hongo and Teresa Bange for their diligence in reviewing thousands of materials during the literature review process. To the Population Council Librarian, Mary Chu, we appreciate your support in retrieving the literature from the various databases. Thank you, Chi-Chi Undie, Esther Lwanga Walgwe and Francis Obare of Population Council for the technical support in editing and reviewing the content. We recognize the editorial support provided by Green Ink and the design support provided by Be-nussy & The Fish.

This study was funded through the UNFPA–UNICEF Joint Programme on the Elimination of Female Genital Mutilation: Accelerating Change, which is generously supported by the Governments of Austria, France, Iceland, Italy, Luxembourg, Norway, AECID (Spain), Sweden, the United Kingdom and the United States of America, as well as the European Union.

© UNICEF/UNI394650/Dejongh

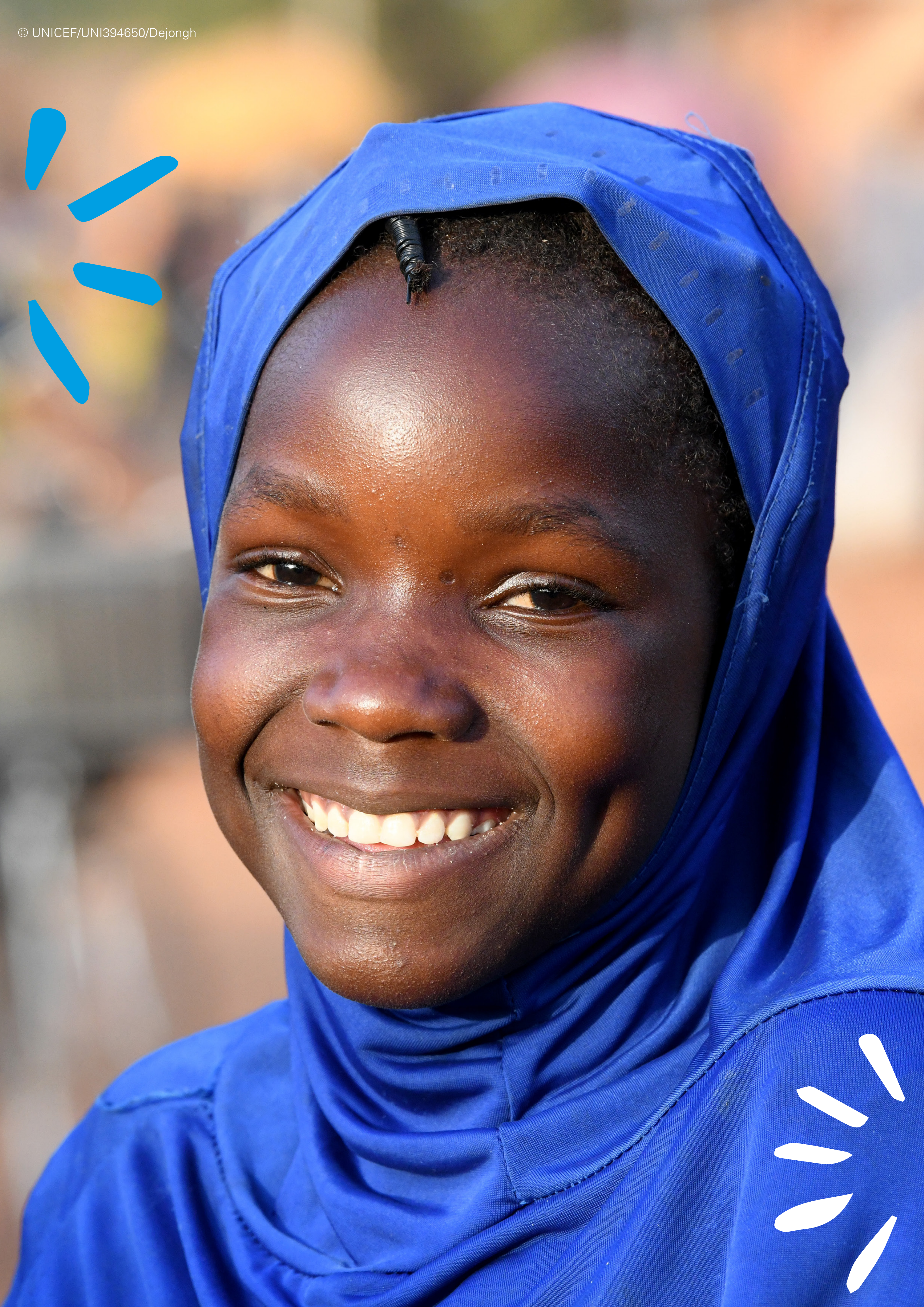

LIST OF ACRONYMS

ARP	Alternative rites of passage
CEEP	Community education and empowerment programmes
COVID-19	Coronavirus disease 2019
DfID	Department for International Development
EXP	Experiment
FGC	Female genital cutting
FGM/C	Female genital mutilation/cutting
FGM	Female genital mutilation
OBS	Observational/non-experimental
OR	Other review
QEX	Quasi-experimental
REA	Rapid Evidence Assessment
SDG	Sustainable Development Goals
UN	United Nations
UNESCO	United Nations Educational, Scientific and Cultural Organization
UNFPA	United Nations Population Fund
UNICEF	United Nations Children's Fund
WHO	World Health Organization

EXECUTIVE SUMMARY

Background

Girls are one-third less likely to be subjected to female genital mutilation (FGM) today than 30 years ago. However, rapid population growth in some of the world's poorest countries, where FGM persists, threatens to roll back progress. In 2020 alone, an estimated 4.1 million girls were at risk of being subjected to FGM. Without concerted and accelerated actions to end the practice, an estimated 68 million additional girls will have been subjected to FGM by 2030. Despite intensified global research efforts to inform strategies to address FGM, there has been little synergy between evidence generation and the implementation of programmes and policy designed to end the practice. As the final decade of acceleration towards zero new cases of FGM by 2030 begins, increasing the rigour, relevance, and utility of research for programming, policy development and resource allocation is critical. This study aimed to synthesize and assess the quality and strength of existing evidence on FGM interventions reported between 2008 and 2020, mapped onto the current theory of change for FGM programming. Study findings will contribute to strengthening the synergy between evidence generation and FGM programmes and inform a global research agenda for FGM.

Methods

This study drew on a Rapid Evidence Assessment of the available literature on FGM interventions published from 2008–2020. We conducted a systematic search of the literature in scientific databases. We also searched literature from websites of institutions or organizations involved in FGM work (n=45). Additional literature was identified by searching references of retrieved studies and suggestions from experts in the FGM field. The quality of studies was assessed using the 'How to Note: Assessing the Strength of Evidence' guidelines published by the United Kingdom's Department for International Development. The strength of evidence was evaluated using a modified Gray scale that has been previously applied to other reproductive health interventions.

Results

Of the 7,698 records retrieved, 115 studies met the inclusion criteria. Of the 115 studies included in the final analysis, 106 were of high and moderate quality. Study findings were organised according to the four levels of the multisectoral approach underpinning the UNFPA–UNICEF Joint Programme on the Elimination of Female Genital Mutilation: Accelerating Change's global, overarching theory of change. At the system level, legislation accompanied by political will, in combination with additional interventions such as sensitization and locally appropriate enforcement

mechanisms, are promising practices in reducing FGM. However, law and legal enforcement take a long time to produce results. At the community level, health education and community dialogues with parents and religious leaders can change attitudes towards FGM, an important step in the continuum of change towards the abandonment of the practice. Media and social marketing efforts are effective in changing social norms and attitudes towards abandoning and, in some cases, reducing FGM. Notably, efforts to convert and/or provide traditional practitioners with alternative sources of income are not effective in eliminating FGM. At the individual level, formal education (educating mothers) can reduce the number of girls being subjected to FGM, while educating girls leads to improved knowledge on the consequences of FGM and changing attitudes towards the need for the practice. At the service level, available evidence, though limited, shows that health-care provider training can improve the capacity for prevention and treatment of FGM. Notably, most of the studies addressed intermediate outcomes for behavioural change such as knowledge and attitudinal change.

Conclusions

At the system level, legislation-related interventions must be multifaceted to be effective. Community-level interventions are effective for changing attitudes towards FGM, but more must be done to innovate with these interventions so that they move beyond affecting attitudes alone to creating behaviour change. At the individual level, formal education is effective in reducing FGM prevalence among girls. However, the returns of formal education in ending FGM may take many years to be realized, so interventions targeting intermediate outcomes, such as improvement in knowledge and change in attitudes and beliefs towards FGM, are equally needed at the individual level. More research is needed at the service level, especially into how the health system can effectively prevent and respond to FGM.

© UNFPA/lz033

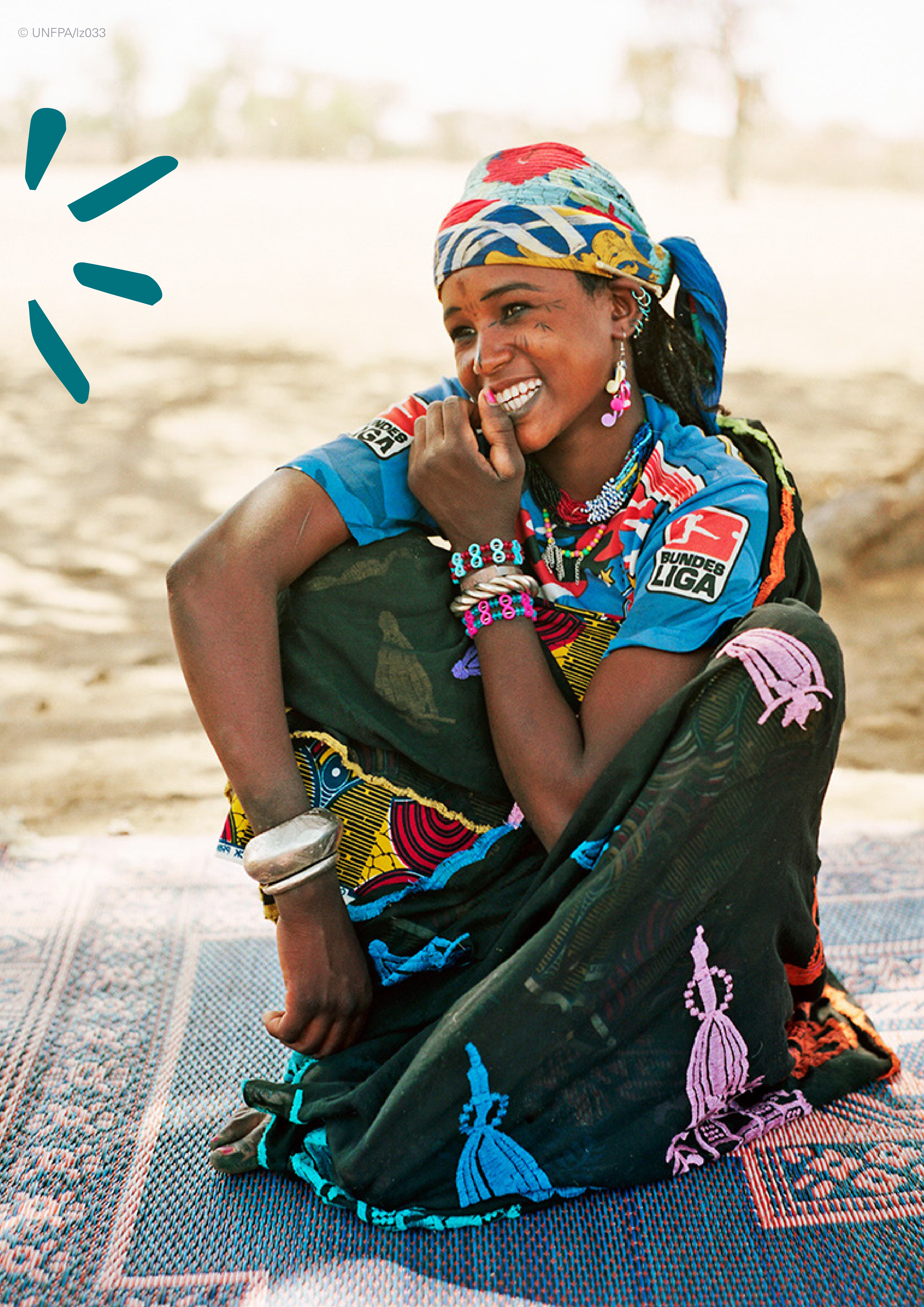

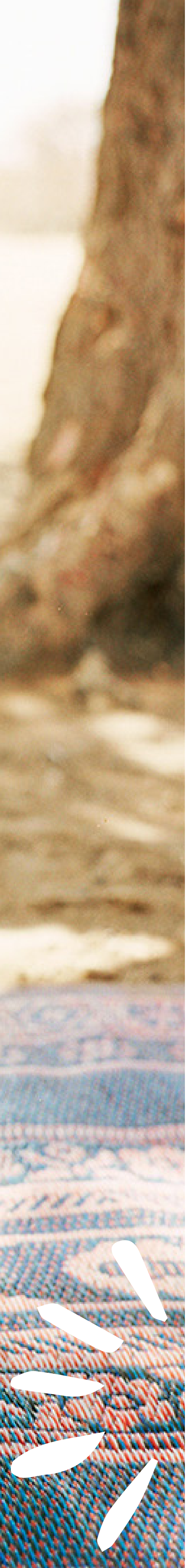

BACK— GROUND

Introduction
Study objectives

Introduction

Female genital mutilation (FGM) is a practice that "involves partial or total removal of the female external genitalia or other injury to the female genital organs for non-medical reasons" (Office of the United Nations High Commissioner for Human Rights (OHCHR), et al., 2008). FGM was officially recognized as a form of violence against women and a violation of human rights in 1993 (United Nations, 1993). The practice has no health benefits and is internationally recognized as a violation of girls' and women's human rights (United Nations, 2015). In 1995, the Beijing Declaration and Platform for Action advanced the international community's commitment to ending FGM by galvanizing the support of national governments (United Nations, 1995).

The World Health Organization (WHO) has classified FGM into four broad categories: FGM Type I, also called clitoridectomy (partial or total removal of the clitoral glans and/or the prepuce); FGM Type II, also called excision (partial or total removal of the clitoral glans and labia minora, with or without excision of the labia majora); FGM Type III, also called infibulation (narrowing of the vaginal orifice by cutting and bringing together the labia minora and/or the labia majora to create a type of seal, with or without excision of the clitoris; in most instances, the cut edges of the labia are stitched together); and FGM Type IV, which includes all other harmful procedures to the female genitalia for non-medical purposes, such as pricking, piercing, incising, scraping and cauterization (OHCHR et al., 2008).

Across the globe, more than 200 million girls and women alive today have been subjected to FGM in 31 countries with nationally representative data in Africa, the Middle East and Asia (Shell-Duncan et al., 2016; UNICEF, 2020). While girls are one third less likely today to be subjected to the harmful practice than 30 years ago, rapid population growth in some of the world's poorest countries where FGM persists threatens to roll back progress (Shell-Duncan et al., 2016; UNFPA, 2019; UNICEF, 2020). In 2020 alone, an estimated 4.1 million girls were at risk of being subjected to FGM, with the number of girls subjected to FGM each year projected to rise to 4.6 million by 2030 (UNFPA, 2019; UNICEF, 2020). Without concerted and accelerated actions, an estimated 68 million additional girls will have been subjected to FGM by 2030 (UNFPA, 2019).

The emergence of the COVID-19 pandemic in 2020 has had an unprecedented impact on the lives and livelihoods of millions of people around the world. The pandemic is also likely to have a huge impact on efforts towards achieving zero new cases of FGM by 2030, as envisaged in the Sustainable Development Goals (SDGs). Measures to contain the spread of COVID-19, such as restrictions on movement and social distancing, have directly affected the implementation of FGM interventions. Closure of schools, local and national lockdowns leading to girls spending more time at home and increased economic hardship are exacerbating the problem. Previous projections suggested that scaling up FGM prevention programmes could reduce new cases by 5.3 million between 2020 and 2030 (UNFPA, 2020). However, due to COVID-19 and the scaling down of FGM programmes, this milestone may not be achieved. It is estimated

that 2 million additional FGM cases that would otherwise have been averted could occur over the next decade as a result of the pandemic (UNFPA, 2020). The need to accelerate progress towards FGM elimination is therefore even more pertinent in the context of the COVID-19 pandemic.

Over the last decade (2010–2020), despite intensified efforts to conduct research globally on addressing FGM, there has been a lack of rigorous high-quality evidence on interventions that are effective in ending the practice. Knowledge of what works has remained elusive, partly due to the limited synergy between evidence on ending FGM and programme and policy implementation. The disconnect between research and programming is reflected in the limited uptake of evidence-based FGM findings to inform policy and programmes and to support mobilization of resources to end the practice. Much of the research conducted to date has not adequately engaged key stakeholders, including researchers, programme implementers and policymakers, from the outset. Other reasons for the limited uptake of evidence include inadequate communication of evidence-based findings and insufficient support or budgets to utilize and operationalize research findings. In addition, inadequate monitoring and evaluation indicators and a lack of coordination between programme personnel and research practitioners in the sector has posed challenges for determining the effectiveness of FGM programmes, while also resulting in research agendas that are not well-aligned with programme needs (UNFPA–UNICEF, 2017).

A number of studies have collated evidence on the effectiveness of interventions to end FGM, either through systematic or non-systematic reviews (Baillot et al., 2018; Berg and Denison, 2012b, 2012a, 2013; Denison et al., 2009; Esho et al., 2017; Johansen et al., 2013; PRB, 2013; WHO, 2011). Some of these reviews have only included studies that used experimental and quasi-experimental designs to determine the effectiveness of interventions to prevent FGM. Due to the limited number of such studies in the FGM field, most of these reviews have reported limited evidence (Berg and Denison, 2012a, 2013). Johansen and colleagues reviewed FGM interventions with the aim of identifying what works and what does not in ending the practice (Johansen et al., 2013). Although this review did not assess the quality of studies included in their review, the authors described the merits and demerits of common approaches applied by various organizations to end FGM.

The WHO has also conducted a non-systematic review of FGM programmatic interventions, mostly from Africa, and highlighted successful approaches (WHO, 2011). In assessing the global evidence on interventions for the reduction of FGM, Esho and colleagues synthesized high quality studies investigating FGM interventions between January 2000 and August 2016 (Esho et al., 2017).

The current study builds on the existing evidence by conducting an up-to-date global synthesis of evidence on the effectiveness of FGM interventions, spanning over a decade. In contrast to previous work, the present review considered qualitative, quantitative and mixed methods studies; literature in Arabic, English, and French; and assessed both the quality of

studies and the strength of the evidence to inform the discourse on what works to end FGM.

As the SDG target of zero new cases of FGM by 2030 approaches, increasing the rigour, relevance, and utility of research for programming, policy development and resource allocation is critical. This study contributes towards meeting this need, by synthesizing and grading the existing evidence on FGM interventions from 2008 to 2020 to provide guidance on evidence-informed programming.

Study objectives

This study aimed to achieve the following objectives:

1. Assess the quality of studies that have evaluated interventions for the prevention of, or response to, FGM.
2. Describe the FGM interventions evaluated by studies deemed to be of moderate or high-quality.
   - **2.a** Assess the strength of evidence of such studies.
   - **2.b** Identify interventions that are most promising for FGM prevention or response, based on such studies.

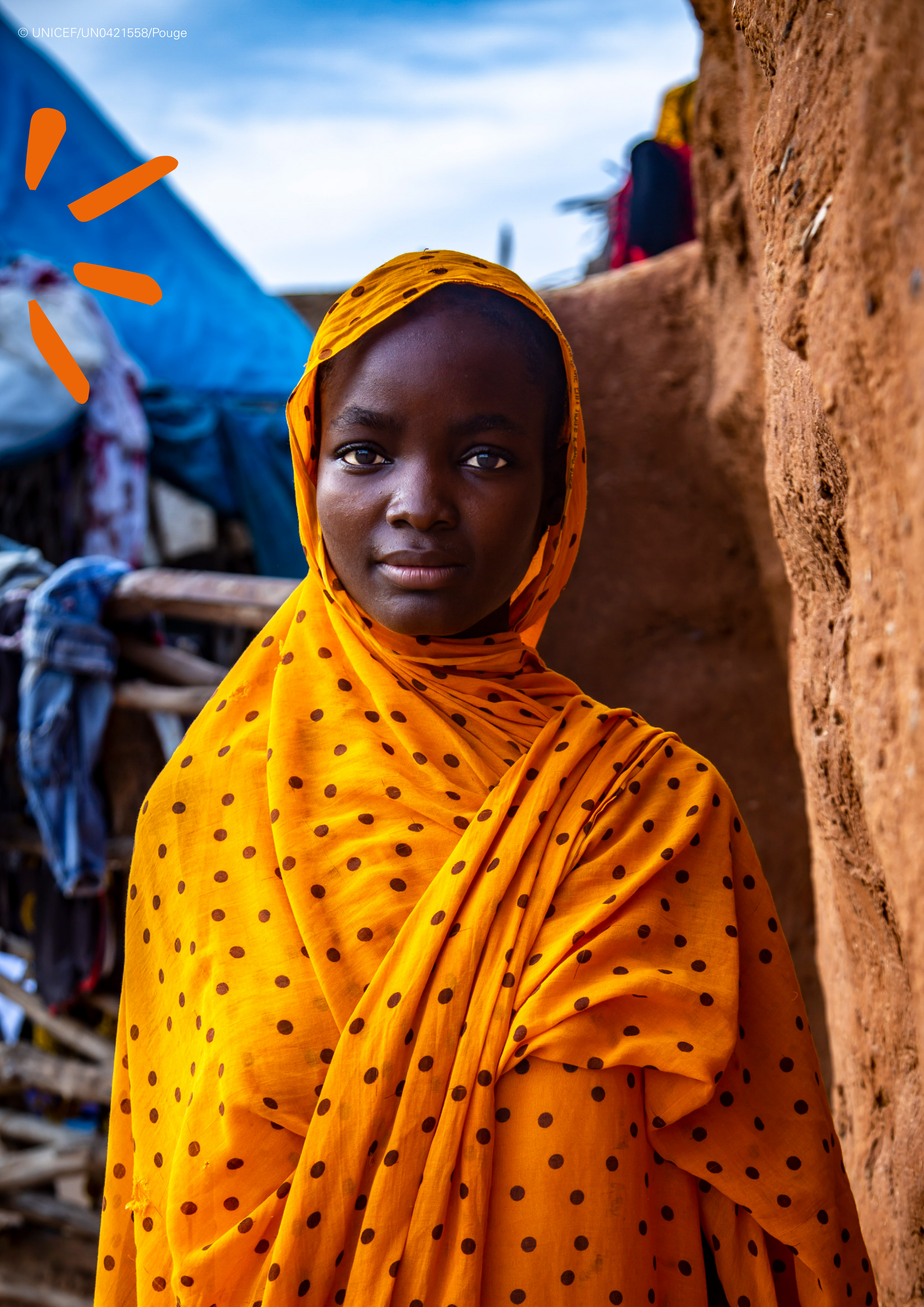

© UNICEF/UN0421558/Pouge

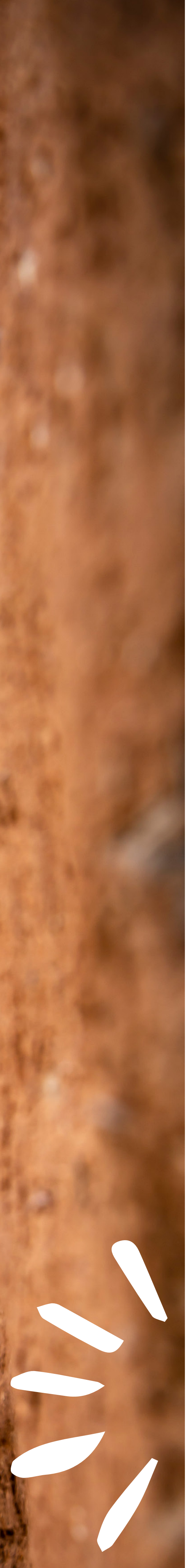

METHODS

Study design
Definition of FGM interventions
Search strategy
Classification of the literature
Assessment of study quality
Describing the quality of the research
Assessing the strength of the research
Quality assurance
Evidence analysis and presentation

Study design

In 2017, the Population Council, under the Evidence to End FGM: Research to Help Girls and Women Thrive programme, conducted a review of studies assessing interventions to support FGM abandonment (Esho et al., 2017). Specifically, the review assessed the quality of studies that have evaluated different interventions for the abandonment or prevention of FGM and described the FGM interventions evaluated by high quality studies. The review used the Rapid Evidence Assessment (REA) approach to synthesize studies investigating FGM interventions from 1 January 2000 to 31 August 2016. The REA is an emerging methodology for locating, appraising, and synthesizing evidence within a short period of time, and is primarily driven by the need to provide timely, rigorous reviews to support evidence-based recommendations (Varker et al., 2015). Within the field of evidence-based practice, policy makers, health care professionals and consumers require timely reviews to inform decisions on efficacious health care and treatments.

The present study uses the REA approach to conduct a comprehensive review of evidence from 1 January 2008 to 31 August 2020. The current study differs from previous work both methodologically and in expansiveness: it considered literature in Arabic, English, and French, and assessed both the quality of studies and the strength of the evidence to make conclusions on what works in ending FGM. Literature from January 2008 onwards was selected because the UNICEF–UNFPA Joint Programme on FGM was established in 2008, heralding increased global attention to FGM in terms of investment in both research and programming.

Definition of FGM interventions

We define an 'FGM intervention' as any form of action or process of intervening, or a deliberate process to interfere with, modify or change people's (both women's and men's) thoughts, feelings, knowledge or behaviours to reduce the prevalence of FGM, or lead to the abandonment of FGM, or to offer care and other services to girls, women, and those indirectly affected by the practice (including men).

Search strategy

The inclusion and exclusion criteria for the literature search were established a priori and are outlined in Table 1. In general, the search included studies published as either evaluation reports, peer-reviewed articles or student theses between January 2008 and August 2020. The key words used in the literature search were: ("female genital mutilation" OR "female genital cutting" OR "female genital mutilation/cutting" OR "FGM" OR "FGC" OR "FGM/C" OR "female circumcision" OR "FGM gash" OR "female genital distortion" "female sexual mutilation" OR "clitoridectomy") AND "interventions".

We conducted a systematic search of literature in the following scientific databases: EBSCO (social sciences database, CHW Wilson, gender studies database, MEDLINE, CINAHL Plus and ERIC), JSTOR, Knowledge Commons, PubMed, SAGE journals, Web of Science and WILEY. Websites of institutions or organizations that have been involved in FGM work (n=45) were purposively identified, according to prior knowledge of their work by the co-authors. We also used references in reports and other literature to identify institutions or organizations involved in FGM work. Additional literature was identified by searching references of retrieved studies and via suggestions from experts in the FGM field. A referencing tool, Zotero, was used to manage all bibliographic references (Zotero, 2021). The references were exported to Covidence for screening and review. Covidence is an online screening and data extraction tool for reviewers conducting standard intervention reviews (Covidence, 2021).

Table 1. Inclusion and exclusion criteria

Considerations	Inclusion criteria	Exclusion criteria
**Geographical location**	Studies investigating FGM interventions, regardless of location	Studies not investigating FGM interventions
**Language**	Arabic, English or French	Literature in languages other than Arabic, English, or French
**Publication date**	1 January 2008 – 31 August 2020	Pre-1 January 2008 and post-31 August 2020 literature
**Publication format**	Evaluation reports, research studies and student theses	Theoretical notes, programme reports not involving evaluations, personal narrations
**Aim of study**	Studies focusing on assessing the effectiveness of interventions in ending FGM	Studies not testing approaches to end FGM
**Study design**	All study designs with clear methodologies for enabling an assessment of quality	Studies without clear methodologies to enable assessment of quality

Classification of the literature

Literature that met the inclusion criteria was categorized as either a primary or secondary study, based on the United Kingdom Department for International Development (DfID) 'How To Note' guidelines (DfID, 2014). Here, primary studies refer to research that observes a phenomenon first-

hand, collects, analyses and/or presents raw data, while secondary studies refer to research that summarizes and interrogates primary study data and findings (DfID, 2014). Table 2 details the various sub-categories under primary and secondary studies.

Table 2.
Framework for general description and study categorization

Study type	Study design	Study method
Primary (PS)	**Experimental (EXP)**	Qualitative; quantitative; mixed methods
	**Quasi-Experimental (QEX)**	Qualitative; quantitative; mixed methods
	**Observational/ non-experimental (OBS)**	Qualitative; quantitative; mixed methods
Secondary (SS)	**Systematic Review (SR)**	Systematic review of literature
	**Other reviews (OR)**	Non-systematic review of literature

Assessment of study quality

In assessing the quality of individual studies, we used the DfID 'How to Note' guidelines (DfID, 2014). Table 3 summarizes the six principles of quality adapted from the 'How to Note' guidelines to assess the quality of primary studies. Studies derived from the review were scored according to indicators aligning with these principles.

For individual studies that met the inclusion criteria, a score of 0 to 2 was given for each quality principle. A score of 2 indicated that the study adhered to all the quality measurement indicators (questions assessing the principle of quality), i.e., all questions received a 'Yes' response; 1 indicated that the study adhered to some of the quality measurement indicators, i.e., one question received a 'Yes' response, and the other received a 'No'; and 0 indicated that the study did not adhere to any of the quality measurement indicators, i.e., all questions received a 'No' response. The scores for each principle of quality were then summed, assuming equal weighting for each principle. A primary study with an aggregate score of 0–4 was considered of low quality; a study with a score of 5–8 was considered moderate quality, and a study with a score of 9–12 was considered high quality.

Table 3. Quality assessment for primary studies

Principles of quality	Indicators	Score
Conceptual framing	Does the study acknowledge existing research?<br>Does the study pose a research question or outline a hypothesis?	0 All questions received a 'No' response<br>1 One question received a 'Yes' response<br>2 All questions received a 'Yes' response
Transparency	Does the study present or link to the raw data it analyses?<br>Does the study declare sources of support/funding?	0 All questions received a 'No' response<br>1 One question received a 'Yes' response<br>2 All questions received a 'Yes' response
Appropriateness	Does the study identify a research design, methods, and analysis approach that is appropriate?<br>Does the study demonstrate why the chosen design and method are well suited to the research question?	0 All questions received a 'No' response<br>1 One question received a 'Yes' response<br>2 All questions received a 'Yes' response
Cultural/context sensitivity	Does the study specify the geography/context in which it was conducted?<br>Does the study explicitly consider any context-specific cultural factors that may bias the analysis/findings?	0 All questions received a 'No' response<br>1 One question received a 'Yes' response<br>2 All questions received a 'Yes' response
Validity	Does the study minimize the possibility that some confounding or unseen variable is affecting any changes observed?<br>Does the study specify how representative the sample used is?	0 All questions received a 'No' response<br>1 One question received a 'Yes' response<br>2 All questions received a 'Yes' response
Reliability	Does the study specify the measures that were put in place to ensure consistency of data collection?<br>Does the study specify the extent to which the measures used are internally reliable?	0 All questions received a 'No' response<br>1 One question received a 'Yes' response<br>2 All questions received a 'Yes' response
**Score (sum)**	0–4 (low quality),<br>5–8 (moderate quality)<br>9–12 (high quality)	0–6

Table 4 summarizes the principles of quality and indicators used to assess the quality of secondary studies. Studies were assigned a score between 0 and 2 for each principle of quality. A score of 2 indicated no limitations identified in relation to the indicator, i.e., the question received a ‘Yes’ response. A score of 1 denoted a lack of clarity, or ‘Unclear’, meaning the study tried to respond to the indicator being assessed, but did not explicitly describe what it entailed. A score of 0, or ‘No’, was assigned if a study did not address the principle being assessed. The aggregate score for each study ranged from 0 to 6. The score ranges for assessing the quality of secondary studies were: 5–6 (high quality), 3–4 (moderate quality), and 0–2 (low quality).

Table 4.
Quality assessment for secondary studies (systematic/non-systematic reviews)

Principles of quality	Indicators	Scoring
**Transparency**	Does the study describe where and how studies were selected for inclusion?	**No = 0**<br>**Unclear = 1**<br>**Yes = 2**
**Validity**	Does the study assess/consider the quality of the studies included?	**No = 0**<br>**Unclear = 1**<br>**Yes = 2**
**Reliability**	Does the study draw appropriate conclusions based on the reviews conducted?	**No = 0**<br>**Unclear = 1**<br>**Yes = 2**
**Score (sum)**	**0–2** (low quality),<br>**3–4** (moderate quality)<br>**5–6** (high quality)	**0–6**

Describing the quality of the research

Studies are described using the format prescribed in the ‘How to Note’ guidelines, i.e.: ‘<Author Name Year>’, ‘<Study type>’; ‘<Quality of evidence>’.

THE SUMMARY ARROW DESCRIPTORS HAVE BEEN USED TO DENOTE THE QUALITY OF STUDIES:

↑ high quality

→ moderate quality

↓ low quality.

Assessing the strength of the research

The strength of the studies was evaluated using a modified Gray scale (Gay et al., 2016; Gray and Chambers, 1997; Gray, 2009). The Gray Scale was developed by Sir John Muir Gray, founder of the Cochrane Reviews, to translate medical evidence into public health policy and practice. Originally developed with five levels of evidence, the Gray scale was subsequently modified by Gay and colleagues to sub-divide the level three classification into studies and evaluations whose designs include control groups (IIIa) and those that do not (IIIb) (Gay et al., 2016) (Table 5). It has also been used by the United Nations Educational, Scientific and Cultural Organization (UNESCO) in a review of early and unintended pregnancy and the education sector, and by Huber et al., in a review of post-abortion care interventions (Huber et al., 2016; UNESCO, 2017). The Gray scale is also

the basis for assessing strength of evidence in the Family Planning High Impact Practice Initiative (www.fphighimpactpractices.org).

While there are numerous scales for evaluating the strength of evidence (e.g., the WHO uses the Grading of Recommendations, Assessment, Development and Evaluations [GRADE] framework to assess clinical interventions and develop guidelines), the Gray scale lends itself particularly well to public health programmatic work and policymaking. In this review, the modified Gray ratings identify the strength of the intervention of a particular study, which is later used to assess a body of evidence based on the methodology listed in Table 5.

To assess the strength of a body of evidence (for example, legal strategies related to FGM), criteria are set for the number of studies, the number of countries and the Gray scale ratings for each study. Promising and successful interventions are based on the range of related studies and their Gray ratings. Criteria to rank policies and interventions as successful, promising, or not effective are explained in the 'What works in FGM prevention and response?' section of this report.

Type	Strength of evidence
I	Systematic review of multiple well-designed, randomized controlled trials.
II	Well-designed, randomized controlled trial of sufficient size.
IIIa	Well-designed trial/study without randomization that includes a control group (e.g., quasi-experimental, matched case-control studies, pre-post with control group).
IIIb	Well-designed trial/study without randomization that does not include a control group (e.g., single group pre-post, cohort, time series/interrupted time series, repeated cross-sectional studies).
IV	Well-designed, non-experimental study from more than one centre or research group, qualitative studies, and/or analysis of routine data.
V	Opinions of respected authorities, based on clinical evidence, descriptive studies, or reports of expert committees.

Table 5.
Modified Gray scale of the strength of evidence of individual studies

Source: *Gray, 1997; Gray, 2009; Gay et al., 2016.*

Quality assurance

Quality assurance measures were applied at every step of the review. These measures included the following:

We used the Covidence online platform to screen studies and determine whether they met the inclusion criteria. The Covidence platform allowed the importation of studies to its database, facilitated the detection of duplicate studies, and made it possible for a team of reviewers to concurrently screen studies. Covidence randomly assigned each study to two reviewers. Where conflicts arose on whether a study was to be included or not, Covidence flagged the conflict and a third reviewer was involved in resolving the conflict. This process ensured efficiency in study screening and reduced bias in the selection of studies that were included in the final quality assessment and analysis.

At the start of the quality assessment phase, five reviewers with a social science background familiarized themselves with the "How to Note" guidelines. Reviewers were trained to conduct the quality assessment with a focus on the principles of quality assessment. The training allowed the team to develop a shared understanding of the principles of quality and the indicators, and of how to score individual studies while being sensitive to the diverse study methodologies.

Each study was assessed by two reviewers who recorded notes justifying the scores awarded. The two reviewers compared their scores and held discussions to resolve any discrepancies. This approach reduced bias in how studies were scored and eventually graded to determine whether they were of low, moderate or high quality.

During the assessment of the strength of evidence, each study was graded by two reviewers who compared their grades and held discussions to resolve any discrepancies. The approach reduced bias in how studies were graded using the modified Gray scale of the strength of evidence.

Evidence analysis and presentation

Data from studies that were graded as being of moderate or high quality were extracted using a pre-designed data extraction form. Some of the key areas in the data extraction form included: author/s name, the study title, year of publication, the study design, intervention description, study outcomes, reasons for success or failure of the intervention and study conclusions. The data extraction form was developed in Microsoft Excel, which allowed the team to crosscheck extraction details and ensure accuracy. Discrepancies in the information extracted were discussed and agreed upon by the review team.

Due to the heterogeneity in study designs, we carried out a thematic analysis focusing on the main themes (interventions) that were evident in the studies. We used a narrative synthesis: an approach that synthesises findings from multiple studies relying primarily on the use of words and text, to summarize and explain the findings on the effectiveness of interventions and the reasons given for their effectiveness, or lack thereof. For the current analysis, moderate and high-quality studies were used to either corroborate or contrast study findings on the effectiveness of the various interventions. Interventions were then reviewed and assigned a Gray rating based on the criteria in Table 5.

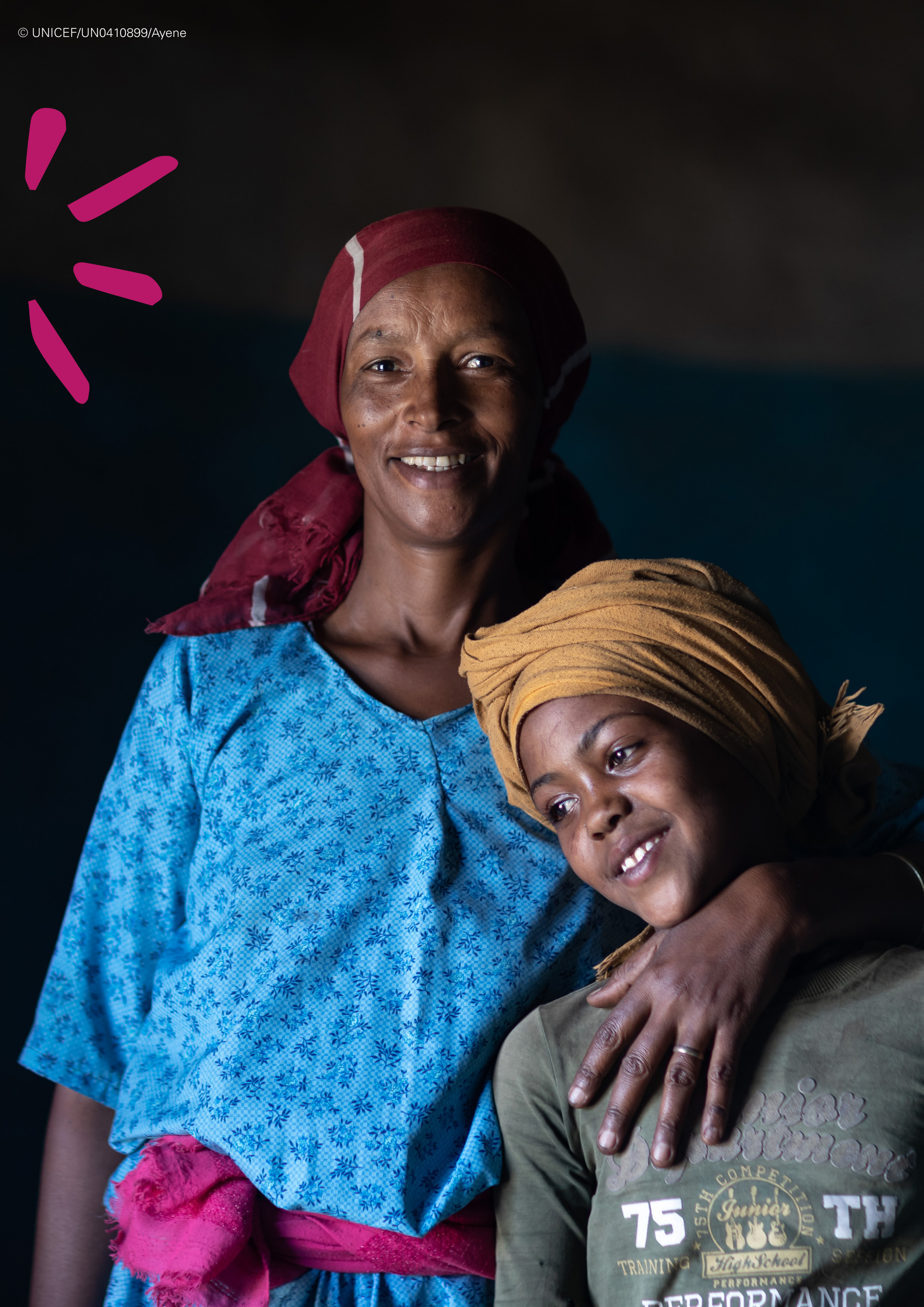

© UNICEF/UN0410899/Ayene

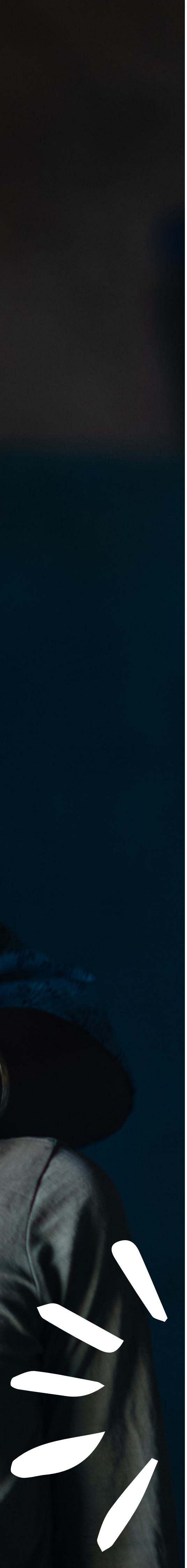

RESULTS

Description of studies assessed

Study identification, screening and eligibility assessment
Timeline of publications
Geographic location of publications
Type of publications retrieved
Quality of studies, research type and design

Classification of FGM interventions

Types of FGM intervention: moderate and high-quality studies

A. System level: Enabling environment for ending Legislative interventions

B. Community level: Addressing gender inequalities and social norms

Health education
Community engagement approaches
Media/social marketing campaigns/communication
Public declaration/statements
Religious/cultural leaders
Conversion of traditional practitioners

C. Individual level: Empowering girls and women

Formal education
Alternative rites of passage

D. Service level: Services for FGM prevention, protection and care

Training health-care providers/capacity-building of the health-care system
Rescue centres

What works in FGM prevention and response?

Successful interventions with supporting evidence
Promising interventions that need further evidence
Interventions lacking evidence

Description of studies assessed

Study identification, screening and eligibility assessment

Figure 1 shows a Preferred Reporting Items for Systematic Reviews and Meta-Analyses (PRISMA) flow chart summarizing the number of studies identified, screened, assessed for eligibility, and included in the final analysis. Of 7,698 records retrieved, 115 studies met the inclusion criteria. Of the 115 studies included in the final analysis, 106 were of high or moderate quality. There is no recommended minimum or maximum number of studies to be included in a systematic review or REA; rather, the number is determined by the search strategy (Harari et al., 2020). Notably, none of the studies published in Arabic met the inclusion criteria, while the studies published in French were duplicates of studies published in English.

Figure 1.
PRISMA flow chart

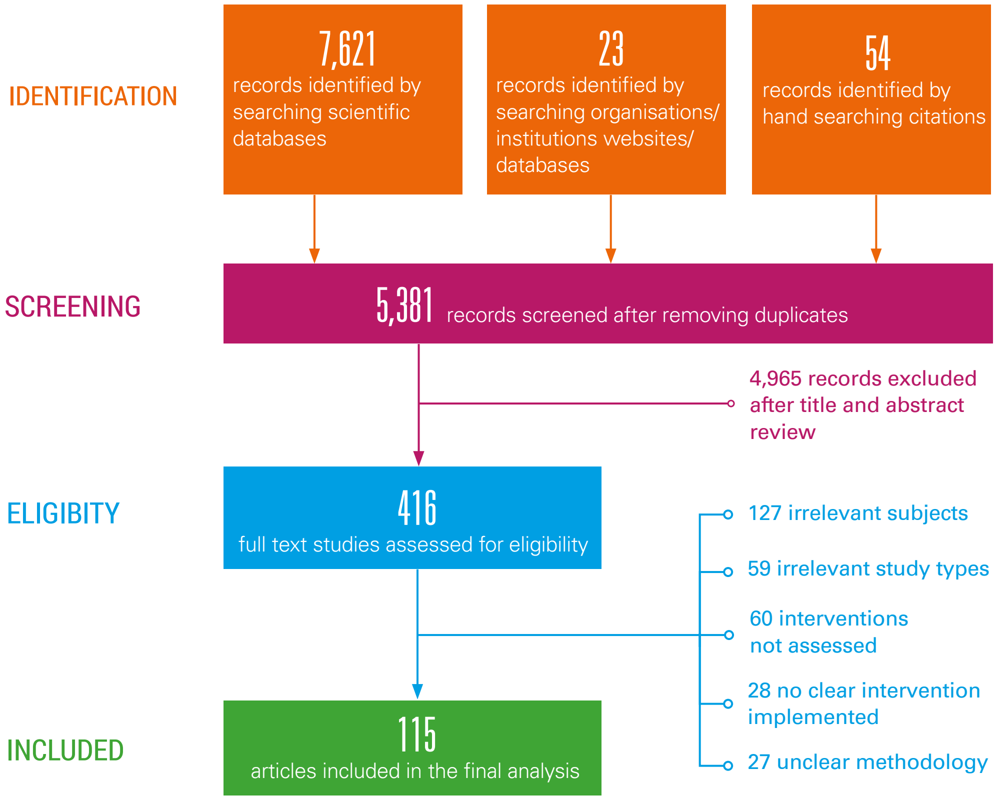

Timeline of publications

In line with the inclusion criteria, the included studies were published between 2008 and 2020. Figure 2 shows the distribution of studies by year of publication. The total number of FGM studies published increased over time. However, there was a decline in publications in 2010, which was followed by a steady increase from 2011 up to 2014. This was followed by a decline in 2015, 2017 and 2018. The highest number of studies were published in 2019 and 2020.

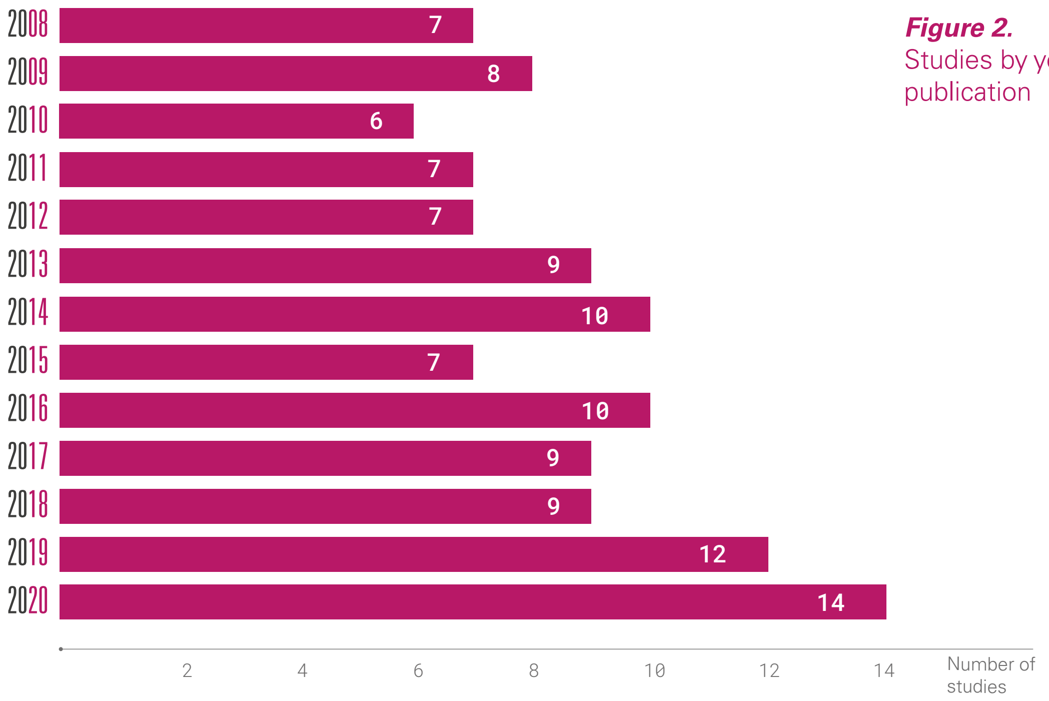

Figure 2.
Studies by year of publication

Geographic location of publications

The majority of the studies (82 out of 115, or 71 per cent) were carried out in Africa; 15 (13 per cent) were based in Europe; and 3 (3 per cent) were conducted in Asia. The remaining 15 of the 115 studies (13 per cent) were inter-regional and mostly reviewed interventions across the globe (*see Figure 3*).

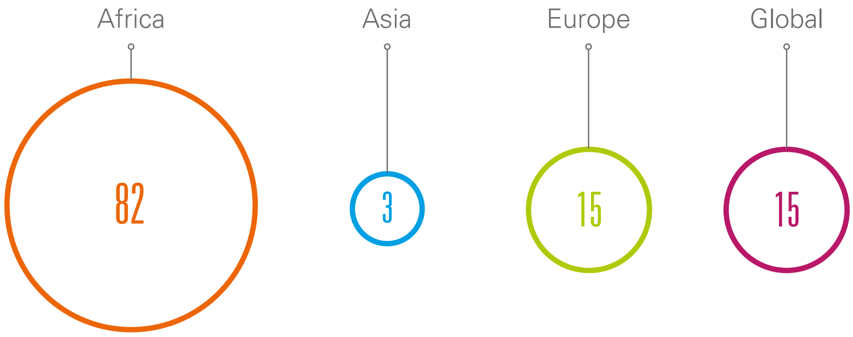

Figure 3.
Geographic location of publications

Type of publications retrieved

The majority of the studies (68 out of 115, or 59 per cent) were peer-review journal articles, 33 (29 per cent) were reports, 8 (7 per cent) were working papers, 5 (4 per cent) were student theses, and 1 (1 per cent) was an evidence brief.

Figure 4.
Studies by types of publications

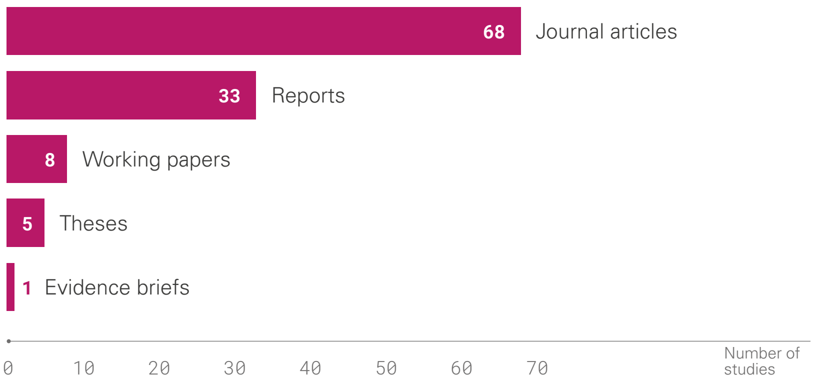

Quality of studies, research type and design

The majority of the studies were either of high quality (52 of 115) or moderate quality (54 of 115). Few studies were considered low quality (9 of 115).

Figure 5.
Quality of studies that met the inclusion criteria

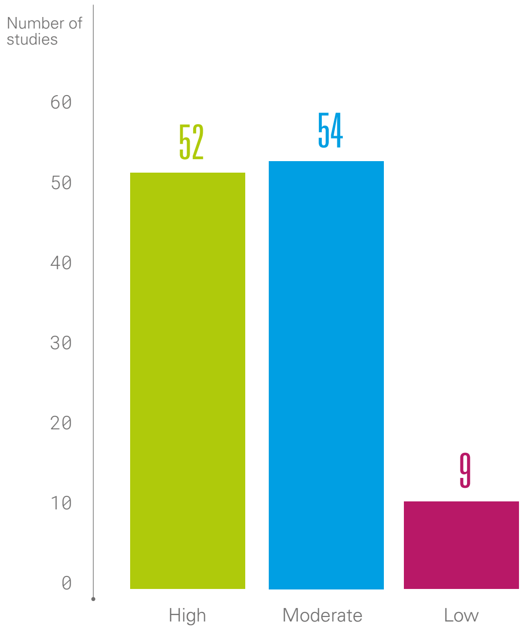

Most of the studies reviewed were primary (87 out of 115) while the rest (28/115) were secondary studies (*see Figure 5*). The majority (81) of the primary studies employed an observational/non-experimental design, four used a quasi-experimental design and two an experimental design. Of the 28 secondary studies, there was an equal number of systematic reviews (14) and non-systematic/other reviews (14).

Classification of FGM interventions

The 115 studies that met the inclusion criteria assessed a diverse range of interventions classified into the following thematic areas (*see Table 6*): legislation/policy measures/human rights; health-care professional training and/or capacity-building of the health-care system, alternative rites of passage, health education, formal education, community engagement, media/social marketing/communication approaches; public statements/declaration; working with religious/cultural leaders, and the conversion of traditional practitioners). Most studies assessed a single type of intervention, although some single interventions involved several strategies and activities. For example, a study might indicate that it focused on a single intervention/theme (such as human rights), while in fact the project employed various communication interventions to reach the target population with human rights messages. Further, some studies incorporated multiple components within the interventions. For example, a project could simultaneously use legislation, community awareness and alternative rites of passage to address FGM. Studies that involved several strategies and activities were classified based on the main intervention that was assessed. Systematic or literature reviews of various types of interventions were classified based on the type of interventions reviewed.

A full description of the studies is provided in Appendix 1.

Table 6.
Types of FGM interventions reported by studies that met the inclusion criteria

Types of FGM interventions	Number of studies
Legal/legislation – any law/policy criminalizing FGM or banning it and human rights approaches	30
Health-care professionals' training/capacity-building of the health-care system – to prevent FGM or manage FGM-related complications	6
Alternative rituals – ceremonies conducted as a rite of passage for girls without undergoing FGM	13
Health education – health risk approaches	9
Formal education – formal schooling/educational attainment	13
Community engagement – community education and empowerment programmes	11
Media/social marketing/communication	18
Public statement/declaration	4
Rescue centres – safe houses for girls running away from FGM	5
Use of religious/cultural leaders	7
Conversion of traditional practitioners	5

Types of FGM intervention: moderate- and high-quality studies

To classify the various intervention approaches, we drew on the UNFPA–UNICEF Joint Programme on FGM global theory of change and the compendium of indicators for measuring the effectiveness of FGM interventions (UNFPA–UNICEF, 2017, 2020). Both the global theory of change and the compendium of indicators embrace a holistic and multisectoral approach to ending FGM. Approaches are categorized as either system level (providing an enabling environment for ending FGM), community level (challenging gender and social norms around FGM), individual level (empowering women and girls), or service level (providing services for FGM prevention, protection and care) (UNFPA–UNICEF, 2017, 2020). Interventions were therefore considered according to these four broad thematic areas, based on the level at which they were implemented:

System level: Refers to existing policies and legislation for the elimination of FGM; policies and legislation intended to empower and protect women and girls at risk of and/or affected by FGM to access comprehensive services; policies and legislation intended to provide an enabling environment for individuals, families, and communities to accept the norm of not subjecting girls to FGM and increasing girls' agency.

Community level: Refers to interventions that target community members, including women leaders, women's associations, groups of men and boys, as well as religious and traditional leaders, to engage in critical reflection and deliberations on new norms and behaviour to improve well-being. It also includes interventions geared towards building the capacities of community members to motivate others to abandon FGM.

Individual level: Refers to interventions targeting girls and women to improve their economic status, capabilities, and agency. It also includes interventions that seek to promote women's and girls' rights to contribute to the emergence of new egalitarian gender norms.

Service level: Refers to interventions that seek to build and strengthen the capacities of health, social and legal service providers to prevent or respond to FGM. It also includes efforts to mainstream FGM in school curricula and social protection programmes targeting girls and women, as well as provision of legal, social and health services for prevention of and response to FGM.

Figure 7 summarizes the distribution of moderate and high-quality studies published from 2008–2010, 2011–2015 and 2016–2020, according to the four levels of intervention. There were fewer studies conducted at all levels in the 2008–2010 period, compared with 2011–2015 and 2016–2020. From 2008 to 2020, more studies were conducted at the community and system level than at the service level.

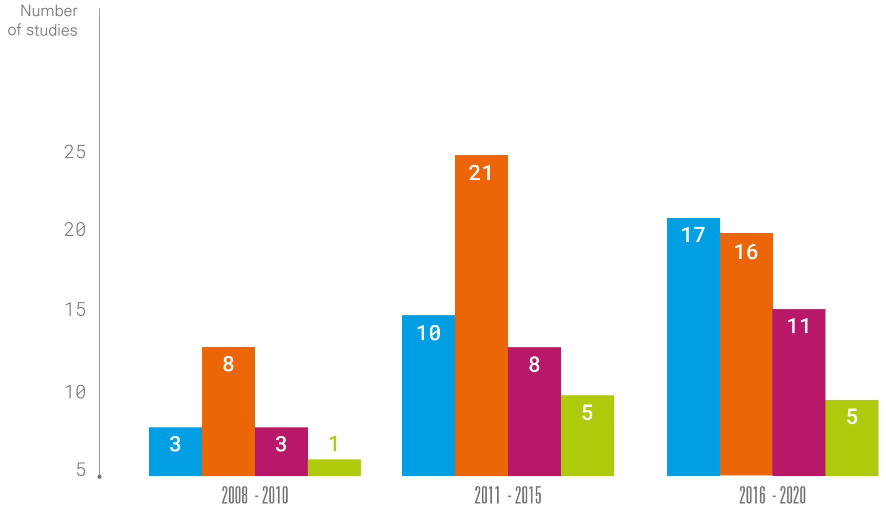

Figure 7.
Studies implemented at the four intervention levels between 2008 and 2020

LEGEND

System level
Community level
Individual level
Service level

A. System level: Enabling environment for ending FGM

System level interventions include those implemented at a macro level to provide an enabling environment for ending FGM. In this review, system level interventions included enactment and legislation-focused interventions.

Legislative interventions

Legislation can provide platforms and avenues upon which other interventions can be safely implemented. Legislation may also serve to accelerate change in FGM practice in environments where community members are already questioning or have abandoned the practice and are seeking social acceptance. Table 7 summarizes the studies that assessed the effectiveness of legislative interventions. A total of 30 studies were identified, of which 13 were of moderate quality and 17 were of high quality. Eight studies were rated at Gray level IIIa or b (evidence from well-designed trials (a) with or (b) without a control group); 16 were at Gray level IV (well-designed, non-experimental research studies), with several of these involving legislative actions as part of a multi-component study, and 5 were Gray level V (descriptive studies or expert opinions based on clinical evidence).

KEY TO DENOTE QUALITY OF STUDY

↑ high quality
→ moderate quality
↓ low quality.

Evidence has shown that political will is critical when legislation is enacted (Boyle and Corl, 2010; S, OR, ↑; Alkhalaileh et al., 2017; P, OBS, ↑)[1]. Johansen (2013) suggests that criminalization of FGM can be effective if there is full commitment and political will within the government and that, in addition to enacting laws, governments should establish programmes, structures and resources to intensify sensitization against the practice (Johansen, 2013; S, OR, →). In the Sudan, Al-Nagar et al. (2017) noted that, in the Sudan's Red Sea State, the State Minister of Health was responsible for issuing a decree forbidding FGM, but there was no specified penalty

1 OBS: observational/non-experimental; OR: other review; P: primary study; S: secondary study.

for offenders. The consequence was that as long as the decree had not been issued, offenders could not be brought to justice; therefore, the law had no practical effect (Al-Nagar et al., 2017; S, OR, ↑). Other studies suggest that enforcement is a critical part of legislation (Meroka-Mutua et al., 2020; P, OBS, ↑; Wouango et al., 2020; P, OBS, ↑; Nambisia, 2014; P, OBS, →).

FGM is performed in many African countries without legal consequences for offenders, despite the existence of laws prohibiting the practice (Ako and Akweongo, 2009; P, OBS, ↑). In most countries, there are no effective mechanisms in place to report, refer, and protect girls and women at risk of FGM. Consequently, the number of court cases is low or non-existent, suggesting a lack of political will and readiness of the community to abandon the practice (Nabaneh and Muula, 2019; P, OBS, →). Notably, some practising communities do not conduct FGM mainly due to fear of the legal consequences of disobedience. This has led to changes in the practice, such as cutting younger girls, performing supposedly less severe cuts, or conducting FGM in secret with more adverse unforeseen effects on girls (Meroka-Mutua et al., 2020; P, OBS, ↑; Matanda et al., 2018; P, OBS, ↑).

This review identified varied effects of the law across regions within the same jurisdiction. Crisman et al. (2016) found that while it is probable that legislation averted FGM among 237,591 girls and women in Burkina Faso in the 10 years since enactment of the law criminalizing the practice, the results varied across regions, possibly due to differences in enforcement, media access, and the pace of community acceptance (Crisman et al., 2016; S, OR, ↑). A recent study by Wouango et al. (2020) showed that 20 years of effort by the Burkina Faso government since the enactment of laws and policies against FGM were bearing fruit, with projected future prevalence rates in Burkina Faso lower than those in Mali – a country that does not have a law against FGM. The study nonetheless notes that some families in Burkina Faso continue to defy anti-FGM laws and all related interventions (Wouango et al., 2020; P; OBS; ↑). In Senegal, despite enactment of the law, rural residence, religion (animist), and ethnicity (Poular, Mandingue, Diola and Soninke) were associated with a higher likelihood of undergoing FGM (Kandala and Komba, 2015; P, OBS, ↑).

The enforcement of legislation has, in some cases and especially in developed countries, alienated the intended beneficiaries, as it reduced the number of beneficiaries seeking care such as reproductive health services, and may even have compromised reporting (Plugge et al., 2019; P, OBS, ↑). Johnsdotter (2019) conducted a review of 122 police files, including criminal investigations regarding suspected FGM cases, mostly based on the mandatory examination of Swedish African girls for evidence of the practice. The study reported psychological trauma among girls and parents as the main unintended outcome of the mandatory genital examination of Swedish–African girls (Johnsdotter, 2019; P, OBS, →). Karlsen et al. (2020) also found that unnecessary, repeated, and insensitive questioning, which assumes levels of dishonesty, criminality and risk, fosters distrust and fear in, and ultimately disengagement from, FGM safeguarding services in the United Kingdom (Karlsen et al., 2020; P, OBS, ↑).

Enactment and enforcement of laws sometimes led to adjustments in FGM practice, including lowering the age of cutting and greater secrecy

in performing FGM. Additionally, legislation enacted without consideration of the local context may be counterproductive and even harmful for intended beneficiaries (Boyden, 2012; P, OBS, ↑; Boyden et al., 2013; P, OBS, →). Through analysis of demographic and health survey (DHS) data, Camilotti (2016) found that girls born in a year and a region where the law against FGM had been legally enforced were cut almost one year earlier than those born before the law was enforced. The law had no significant effect in reducing the prevalence of FGM; rather, the age at cutting fell, as girls were cut much earlier, possibly to circumvent the law (Camilotti, 2016; P, OBS, ↑). Cetorelli et al. (2020) used nationally representative data to compare trends in FGM in Mauritania, where a law that prohibits FGM was passed in 2005, with Mali, where there was no such law. They found that the trends in FGM were similar across the two countries and that the 2005 law did not have a significant impact on reducing FGM prevalence in Mauritania (Cetorelli et al., 2020; P, QEX[2], ↑).

Introduction of laws against cultural practices such as FGM that are strongly valued by community members can lead to the practice continuing in secret (Buttia, 2015; S, OR, →). In Ethiopia, child protection laws (enacted and enforced without due regard to context and local nuances) have resulted in the transformation rather than the elimination of the practice and may even have increased risk to girls (Boyden, 2012; P, OBS, →). Laws in some instances incited resistance or drove the practice underground, as reported in Senegal (Shell-Duncan et al., 2013; P, OBS, ↑) and Ghana (Ako and Akweongo, 2009; P, OBS, ↑). Legal interventions have sometimes also resulted in increased medicalization of the practice (Rasheed, Abd-Ellah and Yousef, 2011; P, OBS, →), and in changing the type of cut, as exemplified in Somalia where there was a shift from 'pharaonic' (infibulation or FGM type III) to 'sunna' (FGM type I and II) (Vestbøstad and Blystad, 2014; P, OBS, →).

In summary, evidence suggests that legislation works more effectively where there is political will (Ako and Akweongo, 2009; P, OBS; ↑, Nabaneh and Muula, P; OBS →, Baillot et al., 2018; S; OR; ↑), the existence of locally appropriate enforcement mechanisms (Muthumbi et al., 2015; S, SR[3]; →), a combination of other interventions that are acceptable to the target community, sufficient resources for implementation (Mehari et al., 2020; P, OBS ↑), and sensitization (Kandala and Komba, 2015; P, OBS; ↑). Legislation can also be useful in an environment where it will be applied across a geographical jurisdiction, is clear and leaves little room for misinterpretation (Al-Nagar et al., 2017; P, OR, ↑; Baillot et al., 2018; P, OBS, ↑). Evidence suggests that legislation alone is not effective in changing attitudes towards FGM and its prevalence (Brown and Porter, 2016; P, OBS →; Dowuona-Hammond et al., 2020; OR, →; Camilotti, 2016; P, OBS, ↑; Hassanin et al., 2008; P, OBS, →; Hassanin and Shabaan 2008; P, OBS, →; Cetorelli et al., 2020; QEX, ↑). Rather, legislation must be implemented in tandem with enforcement mechanisms, as well as other interventions, to show impact in knowledge, attitudes and norms driving the practice (Mehari et al., 2020; P, OBS, ↑; Van Bavel, 2020; P, OBS, ↑; Muthumbi et al., 2015; S, SR, →).

2 QEX: quasi-experimental.
3 SR: systematic review.

Table 7. Studies that assessed effectiveness of legislative interventions

Study	County/region	Main objective and outcome	Key finding	Quality	Strength
Cetorelli et al. (2020)	Mali and Mauritania	• Compare FGM trends in Mauritania, where FGM law was passed in 2005, with Mali, where there is no such law<br>• FGM abandonment	The 2005 law did not have a significant impact on reducing FGM prevalence in Mauritania.	↑	IIIa
Shell-Duncan et al. (2013)	Senegal	• Assess community responses to criminalizing FGM<br>• FGM abandonment	Compliance due to fear of prosecution, defiance, secrecy in practising FGM.	↑	IIIa
Mehari et al. (2020)	Ethiopia	• Assess respondents' views about the impact of various interventions they had been exposed to, including legislation<br>• FGM abandonment and change in social norms	Effective with community participation. Low effect where there was no enforcement.	↑	IV
Boyden et al. (2013)	Ethiopia	• Assess effectiveness of child protection laws in ending FGM<br>• FGM abandonment t	Unintended adverse consequences: defiance, secrecy, changes in practices.	→	IV
Vestbøstad and Blystad (2014)	Somaliland	• Review of various interventions including legislation<br>• FGM abandonment	Shift in the type of FGM, from type III to type I and II.	→	IV
Hassanin and Shaaban (2013)	Egypt	• Compare prevalence of FGM before and 5 years after the law<br>• FGM abandonment, attitudes, and medicalization	Little change in FGM prevalence and attitude towards FGM. Reduction in medicalization.	→	IIIa
Ako and Akweongo (2009)	Ghana	• Assess community response to criminalization of FGM<br>• FGM abandonment	A law criminalizing FGM is necessary but not sufficient for elimination purposes.	↑	IV
Dowuona-Hammond et al. (2020)	Ghana	• Desk review of existing law and other documents on women's empowerment<br>• FGM abandonment	Laws alone are insufficient to change negative practices such as FGM.	→	V
Kandala and Komba (2015)	Senegal	• Assess the effect of legislation on FGM prevalence among women and their daughters<br>• FGM abandonment	No change in prevalence in the 5 years between the surveys, with large geographic variations.	↑	IIIb
Nambisia (2014)	Kenya	• Review of various interventions including legislation<br>• FGM abandonment and attitudes	Poor enforcement making the law ineffective.	→	IIIb

Study	County/region	Main objective and outcome	Key finding	Quality	Strength
Brown and Porter (2016)	United Kingdom	• Review of various interventions including legislation<br>• Attitudes	Attitudinal shift towards rejecting FGM and widespread support for ending FGM.	→	IV
Muthumbi et al. (2015)	27 African countries and Yemen	• Systematic review on effectiveness of legislation<br>• FGM prevention and abandonment	Limited impact on attitudes and perceptions of FGM, in some instances counterproductive.	→	IV
Baillot et al. (2018)	Europe	• Scoping review of various interventions including legislation<br>• FGM abandonment	Some promising initiatives to work with diaspora communities in addressing FGM.	↑	IV
Van Bavel et al. (2017)	The United Republic of Tanzania	• Assess the effect of multiple interventions including legislation<br>• FGM abandonment and attitudes	FGM done secretly to avoid prosecution.	↑	IV
Rasheed et al. (2011)	Egypt	• Assess the incidence, experience, knowledge and perceptions of FGM after criminalization<br>• FGM incidence and attitudes	Incidence of FGM still very high despite the law criminalizing the practice. Health-care providers perform most procedures and support FGM.	→	IV
Matanda et al. (2018)	Kenya	• Review of various interventions including legislation<br>• FGM abandonment and social norms	Laws led to changes in FGM practice: secretive, FGM on younger girls and medicalization.	↑	IV
Johnsdotter (2019)	Sweden	• Assess the effect of legislation involving compulsory genital examinations<br>• FGM abandonment	Negative effects including psychological trauma to the girls and parents.	→	V
Crisman et al. (2016)	Burkina Faso	• Assess the impact of legislation on FGM<br>• FGM abandonment	The law reduced FGM, though results varied across regions.	↑	IIIb
Hassanin et al. (2008)	Egypt	• Assess changes in FGM prevalence after enforcement of prohibition law<br>• FGM abandonment	No significant differences in the pattern of FGM over the years.	→	IIIb
Camilotti (2016)	Senegal	• Assess the impact of legislation on FGM<br>• FGM abandonment	The law had limited effect in reducing FGM prevalence; instead, age at cutting fell.	↑	IIIb
Al-Nagar et al. (2017)	the Sudan	• Review of the impact of legislation (Child Act) on FGM<br>• FGM abandonment	The law did not have any practical effect on curbing FGM.	↑	IV

Study	County/region	Main objective and outcome	Key finding	Quality	Strength
Boyden (2012)	Ethiopia	• Assess the impact of child protection laws on harmful traditional practices including FGM<br>• FGM abandonment	Legislation resulted in transformation of FGM rather than its elimination.	↑	IV
Nabaneh and Muula (2019)	Africa	• Review of the legal and ethical landscape in Africa in relation to FGM<br>• FGM abandonment	Criminalization of FGM can be effective if there is a full commitment and political will within the government.	→	V
Wouango et al. (2020)	Burkina Faso and Mali	• A comparative review of changes in FGM in Burkina Faso, where there is FGM legislation, and in Mali, where there is no such legislation<br>• FGM abandonment and attitudes	Efforts by Burkina Faso's government since the enactment of anti-FGM laws have been successful: the projected future prevalence rates for all villages in Burkina Faso are far lower than those of their counterparts in Mali.	↑	IV
Karlsen et al. (2020)	United Kingdom	• Assess the effectiveness of safeguarding services in health care on FGM<br>• Quality of care	Fostered distrust, fear, and disengagement from health services by community members.	↑	IV
Plugge et al. (2019)	England	• Assess effectiveness of FGM prevention measures including legislation<br>• FGM abandonment	Law not sufficient to tackle FGM and might be counterproductive.	↑	IV
Boyle and Corl (2010)	Global	• Review of the intersection between law, culture, and context<br>• FGM abandonment	Elimination of FGM is slow in the global South, and faster in the North where laws are accompanied by enforcement.	↑	V
Meroka-Mutua et al. (2020)	Kenya	• Assess the extent to which people obey the law on FGM<br>• FGM abandonment and attitudes	FGM conducted in secrecy due to fear of the law. Conflict between custom and the law results in non-compliance.	↑	IV
Buttia (2015)	Kenya	• Review of various interventions including legislation against FGM<br>• FGM abandonment and knowledge/awareness	Law effective where it is integrated with other interventions. FGM practice increasingly performed in secret.	→	V
Alkhalaileh et al. (2017)	Egypt	• Assess effectiveness of legislation (law criminalizing FGM passed in Egypt in 2008) on prevalence of FGM<br>• FGM abandonment and attitudes	Reduced prevalence of FGM among all sections of the population except ever-married women	↑	IIIb

B. Community level: Addressing gender inequalities and social norms

This category included interventions implemented in communities with the aim of challenging existing gender inequalities and social norms associated with FGM. It is important to note that studies that met the eligibility criteria tended to focus on social norms, rather than specifically on gender norms. Specific interventions assessed for effectiveness at the community level were health education, comprehensive community engagement, media/social marketing campaigns/communication initiatives, public declarations/statements, working with religious/cultural leaders, and conversion of traditional practitioners.

Health education

Health education interventions were mostly educational campaigns for awareness creation targeted at community members. Several studies described the effects of health education on various aspects of FGM, including awareness, beliefs, and practices (*Table 8*). Most of the studies were of high quality (seven of nine) and strength, with seven out of nine being ranked at Gray IIIa or IIIb.

Health education using the health risk approach can be useful in imparting knowledge related to the physical, psychological, and emotional consequences of FGM (Galukande et al., 2015; P, OBS, ↑). Health education can also trigger and guide discussions among practising communities on the effects of FGM and hence the need for action to eliminate the practice (Diop and Askew, 2009; P, QEX, ↑). Asekun-Olarinmoye and Amusan (2008) conducted a study on health education sessions on FGM and its complications in a rural community in Nigeria. They found that the health education intervention had a positive impact on the attitude of individuals, with a statistically significant increase in the number of men who wanted FGM to be stopped, and a decrease in the number of individuals who intended to have their daughters undergo FGM in the future (Asekun-Olarinmoye and Amusan, 2008; P, OBS, ↑). There were statistically significant increases in the level of knowledge of respondents regarding most of the complications of FGM after intervention except biennia fever, prolonged bleeding, and vulva swelling (Asekun-Olarinmoye and Amusan, 2008; P, OBS, ↑). Another study in Nigeria aimed to provide accurate information to outpatient women at a health-care facility to dispel the traditional myths and beliefs about FGM. The study found statistically significant changes in women's knowledge, beliefs, and attitudes towards FGM (Ekwueme et al., 2010; P, OBS, ↑).

In Iraq, an investigation into the effectiveness of a short-term health education intervention to change the attitudes of parents and religious leaders towards FGM found that the support of Mullahs and Mokhtars for the abandonment of FGM increased by 35 per cent and 41 per cent, respectively (Abdulah et al., 2020; P, OBS, ↑). An intervention in the United Republic of Tanzania that implemented a health education programme over an 18-month period showed moderate success in increasing knowledge

of the health risks and changing attitudes towards FGM (Galukande et al., 2019; P, OBS, ↑). Similarly, in the Sudan, a school-based health education programme targeting girls showed a positive impact on both the knowledge and attitude of female students towards FGM (Mahgoub et al., 2019; P, OBS, ↑).

A systematic review of educational sessions showed that the effects of health education across communities and target groups may not be uniform (Berg, 2013; S, SR, ↑). The review found that while educational sessions did not have any effect on health-care providers, educational sessions about reproductive health, including the dangers of FGM, seemed to improve awareness about the practice among female students. The review also summarized some key findings from relevant studies: for example, an extended communication programme targeting Nigerian villagers improved understanding of FGM in both men and women; and knowledge of harmful consequences of FGM increased among Ethiopian participants, but not among Somali participants from the same environment.

The way health education is packaged may have a negative impact, as shown by Winterbottom et al. (2009). The authors posit that the negative effects of FGM interventions (including health education) in the United Republic of Tanzania were due to framing of the practice as a 'tradition' rooted in a 'primitive' and unchanging culture, leading to resistance from the target group (Winterbottom et al., 2009; S, OR, →).

In summary, while the effects of health education appear promising, literature and findings from multifaceted interventions suggest that health education can be more effective in an environment where context is considered and other interventions are also implemented (Waigwa et al., 2018; S, SR, ↑). It is also suggested that while health education may be effective in changing knowledge, attitudes, and beliefs, an additional intervention may be needed to influence behaviour change (WHO, 2011; S, OR, →).

Table 8. Studies that assessed effectiveness of health education interventions

Study	Country/ region	Main objective and outcome	Key finding	Quality	Strength
Galukande et al. (2015)	The United Republic of Tanzania	• Assess effectiveness of educational campaigns for awareness creation on FGM<br>• FGM abandonment, knowledge, and attitudes	Moderate success in increasing knowledge and changing attitudes. Effectiveness in reducing FGM uncertain. FGM done secretly.	↑	IIIb
Diop and Askew (2009)	Senegal	• Assess effectiveness of multiple interventions including health education on FGM<br>• FGM abandonment, knowledge, and attitudes	Reduction in FGM, improvements in knowledge and attitudes towards FGM	↑	IIIa
Asekun-Olarinmoye and Amusan (2008)	Nigeria	• Assess effectiveness of health talks in vernacular language on FGM<br>• Attitudes	Increase in support to end FGM. Decrease in intention to have daughters mutilated in the future.	↑	IIIb
Abdulah et al. (2020)	Iraq	• Assess effectiveness of community-based health education on FGM<br>• Attitudes	Change from a position of supporting FGM to intention to abandon FGM and not cut daughters in future.	↑	IIIb
Mahgoub et al. (2019)	the Sudan	• Assess effectiveness of school-based health education on FGM<br>• Knowledge and attitudes	Improvement in knowledge and attitude towards FGM	↑	IIIa
Waigwa et al. (2018)	Egypt, Ethiopia, Ghana, Kenya, Morocco, Nigeria and Senegal	• Systematic review of health education interventions to prevent FGM<br>• FGM prevention	Effectiveness depends on sociodemographic factors; traditions and beliefs; intervention strategy, structure, and delivery.	↑	IIIa
Winterbottom et al. (2009)	The United Republic of Tanzania	• Review of interventions that framed FGM as backward and primitive<br>• FGM abandonment	Ineffective and counterproductive	→	V
Ekwueme et al. (2010)	Nigeria	• Assess effectiveness of health education on FGM at a tertiary health institution<br>• Knowledge and attitudes	Increase in knowledge, beliefs, and improvement in attitudes towards FGM	↑	IIIb
WHO (2011)	Global	• Review of various interventions including health education<br>• FGM abandonment	Interventions that only supply information and education not effective in changing behaviour	→	V

Community engagement approaches

Community engagement approaches create a platform for internal community discussions on harmful traditional practices such as FGM. A total of nine studies – three of high quality and Gray rating and six of moderate quality and Gray rating – assessed the impact of community engagement approaches on FGM (*Table 9*). Most of the studies focused on community dialogues or conversations where participants were expected to begin to question the role of some practices that are proven to have negative consequences, and therefore facilitate change.

A systematic review of interventions to end FGM found that an underlying change mechanism was that providing information about FGM would increase knowledge and change attitudes (Berg and Denison, 2013; S, SR, ↑). The evidence suggested that the key to changing FGM-related behaviour was how the information was disseminated (Berg and Denison, 2013; S, SR, ↑). Using a holistic approach, community education and empowerment programmes (CEEP) enable communities to receive formal and informal education through four modules which include human rights, problem-solving, basic hygiene, and women's health. The most well-known CEEP is Tostan, which began in Senegal in the 1980s and has since been implemented in other African countries including Burkina Faso, Ethiopia, Mali and Somalia (Diop and Askew, 2009).

In Senegal, Diop and Askew (2009) used a quasi-experimental study to test the effectiveness of CEEP in changing attitudes towards FGM and the prevalence of the practice. The findings showed that information from the programme was diffused widely within the intervention villages and led to significant improvements in knowledge about and critical attitudes towards FGM among women and men who had or had not participated in the programme, without a corresponding improvement in the comparison villages. There were also significant changes in the prevalence of FGM among daughters over time, as reported by women who were directly and indirectly exposed to the programme, but not among daughters in the comparison villages (Diop and Askew, 2009; P, QEX, ↑).

In Ethiopia, the Tostan CEEP focused on community conversations, religious dialogues, legislation, and community education on the health effects of FGM by utilizing existing community structures such as churches and mosques. This approach increased awareness and understanding of the adverse health effects of FGM. Perpetrators of FGM were punished, which acted as a deterrent to others carrying out the practice FGM (UNICEF, 2010; P, OBS, ↑).

The Tostan Programme in Somalia focused on delivering the four modules (human rights, problem-solving, basic hygiene and women's health) through the establishment of community participation committees and community outreach. Evidence showed that the programme did not have a significant effect on FGM practice: over 70 per cent of the women in the target area still practised FGM. However, there was a change in perceptions and attitudes towards FGM. These findings from Somalia show that a change in attitude does not always translate to FGM abandonment (UNICEF, 2010; P, OBS, ↑).

A global systematic review of interventions to end FGM that included community change and development demonstrated that successful interventions need to involve, be driven by engage with the whole community. The review found that targeting FGM is most effective and well-received when a broader approach, targeting multiple sectors, is used, and the community is supported to resolve other challenges that are not necessarily related to FGM (Johansen et al., 2013; S, OR, →).

A study in the United Kingdom identified a positive shift in attitude towards FGM where community-based preventive work is taking place (Brown, 2013; P, OBS, →). The study also found that the most effective approach to FGM prevention requires multiple stakeholders at a local level, mainstreaming FGM in other sectors and allowing community groups to play a role in prevention (Brown, 2013; P, OBS, →). In Somalia, a study on the effects of community dialogues on FGM and child marriage found that the dialogues raised awareness of the negative consequences of FGM, which led to medicalization of the practice and a change in the prevalent type of FGM, but not support for total abandonment (Kipchumba et al., 2019; P, OBS, ↑). Although interventions should target total abandonment rather than changes in the type of FGM and medicalization of the practice, these shifts could represent the initial stages in the continuum of change towards abandonment, based on the recognition that change in the practice of FGM is complex and does not happen instantaneously, but occurs gradually.

In summary, community engagement approaches that use a holistic approach and seek to empower community members have been effective in changing attitudes towards FGM and in some cases changing behaviour (Diop and Askew, 2009; P, QEX, ↑; UNICEF, 2010; P, OBS, ↑). Evidence also shows that the use of tailored, contextually appropriate and locally generated interventions where the community is fully engaged can yield positive results in changing behaviour (Barrett et al., 2020; P, OBS, →; Johansen et al., 2013; S, OR, →).

Table 9. Studies that assessed effectiveness of community engagement interventions

Study	County/region	Main objective and outcome	Key finding	Quality	Strength
Diop and Askew (2009)	Senegal	• Assess effectiveness of innovative pedagogical techniques inspired by African traditions and local knowledge with a strong human rights approach in ending FGM<br>• FGM abandonment, knowledge, and attitudes	Improvements in knowledge and attitudes towards FGM; decrease in prevalence of FGM among daughters	↑	IIIa
Berg and Denison (2013)	Burkina Faso, Ethiopia, Kenya, Mali, Nigeria, Senegal	• Systematic review of various interventions including community engagement approaches<br>• FGM abandonment, knowledge, and attitudes	Providing information about FGM increased knowledge and improved attitudes	↑	IIIa
Cislaghi et al. (2019)	Mali, Nepal and Nigeria	• Assess effectiveness of community dialogue in changing social norms associated with FGM<br>• Social norm change	Community dialogue was effective in changing social norms	→	IIIb
Barrett et al. (2020)	Europe	• Assess behaviour change through community engagement using the REPLACE approach<br>• Social norm change and attitudes	Changes in norms and attitudes associated with FGM	→	IV
UNICEF Innocenti Insight (2010)	Egypt, Ethiopia, Kenya, Senegal and the Sudan	• Review of various interventions including community conversations/ engagement<br>• FGM abandonment and attitudes	Some success in using community conversations to change attitudes towards FGM in Ethiopia and the Sudan	→	IV
Ogalleh (2014)	Somaliland	• Assess effectiveness of community education on FGM prevention<br>• Knowledge and attitudes	Not effective due to disjointed intervention approaches	→	V
Brown and Hemmings (2013)	United Kingdom	• Assess multiple interventions including community-based projects<br>• FGM abandonment and attitudes	Growing opposition to FGM and support for a more interventionist stance to be taken by the United Kingdom government in safeguarding all women and girls from FGM	→	V
Johansen et al. (2013)	Global	• Review of various interventions including community change and development interventions<br>• FGM abandonment, knowledge, and attitudes	Broader approaches that assist communities in addressing other challenges than FGM are effective	→	IIIa
Kipchumba et al. (2019)	Somalia	• Review of various interventions including community conversations<br>• FGM abandonment, awareness, and attitudes	Community dialogues raised awareness of negative consequences of FGM. However, FGM prevalence remained almost universal and increase in medicalization.	↑	IIIb

Media/social marketing campaigns/communication

Eight high and eleven moderate quality studies assessed the impact of media, social marketing campaigns and communication on FGM abandonment. Ten studies were categorized as Gray IIIa or b, two as level IV and seven as Gray V (*Table 10*). Most of the interventions focused on using various media platforms to disseminate messages on negative health consequences of FGM.

Research has shown that media can be an effective tool in the push towards FGM abandonment (Kaunga, 2014; P, OBS, →). Mainstream newspapers, television reports, SMS messaging, social media, theatre productions, television and radio melodramas can all shape conversations about FGM and accelerate the shift in social norms towards FGM abandonment (UNFPA, 2017; S, OR, →). In Egypt, women exposed to two or more FGM media messages were 1.6 times more likely than unexposed women to support discontinuing the practice (Suzuki and Meekers, 2008; P, OBS, →). For this to be effective, in the Egyptian context, communication needed to be clear and include religious rulings and medical statements on FGM (Hussein and Ghattass, 2019; P, OBS, →).

Positive shifts in attitudes can be achieved when considerable knowledge of risks is disseminated, enabling the target population to become a source of information and agent of change (Ahmed, 2012; P, OBS, →). Effective sensitization campaigns have empowered girls to refuse FGM and to report to relevant authorities when at risk (Buttia, 2015; S, SR, →). There also appears to be a substantially increased awareness not just of the illegality of FGM, but of the consequences of breaking the law (Brown, 2013; P; OBS; →) and of the negative impacts of infibulations (Mehari et al., 2020, P, OBS, ↑).

Evans et al. (2019) evaluated the effectiveness of the Saleema campaign, which aimed to reduce pro-FGM social norms in the Sudan. The Saleema initiative involved community dialogue on the existence of FGM, its role in society, and the need for abandonment, alongside a public pledge to abandon the practice and wearing Saleema colours as a sign of support. Using a quasi-experimental design controlled for 'dosage' of campaign messages delivered across the 18 states in the Sudan, Evans and colleagues found that, according to self-reported exposure to the social marketing strategy, the Saleema campaign was effective in reducing pro-FGM social norms. Additionally, higher doses of Saleema marketing, measured through an exogenous indicator of campaign event exposure from an independent monitoring system, was significantly associated with reduced pro-FGM social norms (Evans et al., 2019; P, QEX, ↑).

Sensitization and training have also resulted in increased awareness and knowledge of the consequences and dangers of FGM, thereby contributing to abandonment of the practice (Nielssen and Coulibaly, 2014; P, OBS, →). A study by UNFPA showed that awareness campaigns in 17 programme countries resulted in increased disapproval of the practice (UNFPA, 2017; S, SR, →). Awareness campaigns have also led to school-

girls and boys recognizing the harmful effects of FGM, resulting in a higher number of uncut girls than before (Abathun et al., 2018; P, OBS, ↑).

According to Berg and Denison (2013), the driving force for changing FGM-related behaviour lies in the dissemination of information. There was evidence of a shift in perspective regarding FGM through the provision of knowledge and the actions of some which spread to others through social networks (Berg and Denison, 2013; S, SR, ↑). In addition, the use of social media has the potential to change attitudes towards FGM. Hussein and Ghattas (2019) found that a greater proportion of young men and women who reported social media use were supportive of abandonment, compared with their counterparts who were not using social media. Based on a secondary analysis of DHS data, the study found a reduction in the prevalence of FGM among girls aged 15–17 years, from 74 per cent in 2008 to 61 per cent in 2014 (Hussein and Ghattass, 2019; P, OBS, →).

Theatre has also been found to be an effective strategy in fostering change in behaviour and social norms (Ugwu and Ashaver, 2014; P, OBS, →). Vogt and colleagues (2016) developed an implicit association test that used movies to unobtrusively measure attitudes towards cutting in the Sudan. The results showed that movies that consider local diversity in terms of both values and eligibility for marriage can change attitudes to favour the abandonment of FGM (Vogt et al., 2016; P, EXP, ↑). The experiment demonstrated that dramatizing discordant views on cutting within a family can improve attitudes about uncut girls.

The UNFPA–UNICEF Joint Programme argues that young people can use social media to raise awareness about FGM and gender equality. Social media can be used to reach large audiences, organize meetings and engage peers in debate. For example, in Nigeria young people produced a film series on FGM, titled ‘Sandra’s Cross’, that reached 3,370,672 people through Facebook, Twitter and YouTube. In Senegal, the social media campaign #TouchePasAmaSoeur (‘Do not touch my sister’) reached more than 5 million people. The promotion of FGM abandonment across social networks has given communities an opportunity to see the possibility of rapid, widespread change (UNFPA–UNICEF, 2018; P, OBS, ↑)

In summary, based on the strength of the evidence, social media/marketing efforts are effective in changing social norms and attitudes towards abandoning FGM, and, in some cases, reducing the practice. However, evidence shows that interventions which only supply information, education and campaigns to increase FGM awareness are not sufficient to change behaviour (WHO, 2011; P, OBS, →). This is corroborated by findings from a study by the Population Reference Bureau (2013), which found that anti-FGM campaigns focusing exclusively on the negative health consequences of FGM may have inadvertently bolstered the medicalization of the practice (PRB, 2013; P, OBS, →). FGM is an entrenched generational practice and eradicating it in a community requires concerted efforts over an extended period. Advocacy and awareness-raising efforts that take a holistic multisectoral approach constitute best practices that should be sustained in order to maintain their impact for future generations (Barsoum et al., 2011; P, QEX, ↑).

Table 10. Studies that assessed effectiveness of media / social marketing campaigns / communication interventions

Study	Country/ region	Main objective and outcome	Key finding	Quality	Strength
Kaunga (2014)	Kenya	• Assess effectiveness of media strategies and communicating information on FGM<br>• Knowledge/awareness	Use of media and local language was effective in influencing effective communication on FGM	→	IIIb
UNFPA (2017)	Global	• Review of various interventions including mass communicationFGM abandonment and attitudes	Reduced incidences of FGM	→	V
Suzuki and Meekers (2008)	Egypt	• Assess effectiveness of anti-FGM communication messages<br>• FGM prevention and attitudes	Reduced support for FGM after exposure to messages	→	IIIb
Ahmed (2012)	The Sudan	• Assess effectiveness of messaging using multiple platforms on FGM<br>• Knowledge/awareness and attitudes	Increased number of women and men with no intention to cut their daughters. Men less likely to have preference for future partner to be cut	→	IIIb
Buttia (2015)	Kenya	• Review of various interventions including communication interventions<br>• FGM abandonment and knowledge/ awareness	Increased awareness of consequences and reduced prevalence of FGM	→	V
Evans et al. (2019)	The Sudan	• Assess effectiveness of the Saleema social marketing intervention (awareness campaign, social dialogue, public declarations and use of role models)<br>• Social norm change	The social marketing strategy was effective in reducing pro-FGM social norms	↑	IIIa
Hussein and Ghattass (2019)	Egypt	• Assess effectiveness of anti-FGM communication messages through social marketing<br>• FGM abandonment, knowledge, and attitudes	Prevalence of FGM among girls aged 15–17 years fell	→	IIIb
Nielssen and Coulibaly (2014)	Mali	• Assess effectiveness of multiple interventions including communication on FGM<br>• FGM abandonment, knowledge/ awareness, and attitudes	The taboo against speaking about FGM had been broken. Contributed to FGM abandonment	→	V
Ugwu and Ashaver (2014)	Nigeria	• Asses the use of theatre as a medium of communicating the consequences of FGM<br>• Knowledge/awareness and attitudes	People became aware of and ready to take actions on FGM. Theatre helped to reshape community worldview about FGM	→	V
Vogt et al. (2016)	The Sudan	• Assess effectiveness of awareness creation through dramatization on FGM<br>• Attitudes	Significantly improved attitudes towards girls who remain uncut	↑	IIIb
Abathun et al. (2018)	Ethiopia	• Assess effectiveness of school-based awareness campaign<br>• Attitudes	Change in attitude in terms of support for abandonment of FGM	↑	IIIa

Study	Country/ region	Main objective and outcome	Key finding	Quality	Strength
WHO (2011)	Global	• Review of various interventions including advocacy and awareness creation<br>• FGM abandonment	Accurate media effective in creating awareness and behaviour change	→	V
PRB (2013)	Global	• Review of various interventions including awareness campaigns<br>• FGM abandonment	Campaigns focusing exclusively on the negative health consequences of FGM inadvertently bolstered medicalization	→	V
Cloward (2014)	Kenya	• Assess effectiveness of transnational activism/campaigns led by international actors<br>• FGM abandonment, awareness and attitudes	Campaigns can lead to concealment of real behaviour and changes in attitudes	↑	IIIa
Berg and Denison (2013)	Burkina Faso, Ethiopia, Kenya, Mali, Nigeria, Senegal	• Systematic review of various interventions including media communication, outreach, and advocacy<br>• FGM abandonment, knowledge and attitudes	Change in attitude regarding FGM through knowledge acquisition.	↑	IIIa
UNFPA–UNICEF (2018)	Global	• Review of various interventions including communication, mass media, and social marketing campaigns<br>• FGM abandonment and attitudes	Radio shows on the rights of women and girls have a positive impact on norms related to FGM.<br><br>The prevalence of FGM among girls aged 15–19 years declined in 10 of the 17 countries.	↑	IV
Barsoum et al. (2011)	Egypt	• Assess multiple interventions including communication interventions on FGM<br>• FGM abandonment, knowledge and attitudes	Changed views and attitudes towards FGM. Girls in the intervention group less likely to be cut than those in the control group.	↑	IIIa
Brown (2013)	United Kingdom	• Assessment of the FGM Initiative: PEER Research Endline Phase 1<br>• FGM abandonment and attitudes	Where community-based preventive work is taking place, attitudes towards FGM are changing; there is growing opposition to the practice.	→	V
Mehari et al. (2020)	Ethiopia	• Explore community perspectives on changes in social norms and practices associated with FGM<br>• FGM abandonment and social norms	In West Arsi – changes in norms leading to FGM abandonment. In Fafan – no change in norms associated with FGM and no abandonment.	↑	IV

Public declaration/statements

Compared with other types of community-level intervention, there were fewer studies that assessed the effectiveness of public declarations/statements in facilitating FGM abandonment. Four were identified: two of high quality (rated Gray IIIb and IV) and two of moderate quality (Gray rating of IV and V) (*Table 11*). Public declarations by community, religious and political leaders or other influential people in a community was the most common approach used to encourage FGM abandonment.

In Ethiopia, according to a report by UNICEF (2012), due to public declarations there was a consensus that FGM was no longer practiced in the open and that it was gradually being abandoned. However, respondents interviewed indicated that the practice had gone underground in some places, especially in remote rural areas (UNICEF, 2012; P, OBS, ↑). Public declarations of FGM abandonment are critical, as they may signal a commitment and readiness to abandon the practice. Mass and social media and other forms of communication have played a central role in amplifying these public declarations and in encouraging other communities to abandon FGM. In Burkina Faso, an evaluation covering almost a decade of social norm interventions involving public declarations of FGM abandonment found that community members responded positively to the approach. It also found that public declarations, when supported by post-declaration follow-up and support, were highly effective in preventing further cases of FGM. Burkina Faso is making progress towards complete abandonment of the practice (UNFPA–UNICEF, 2018; P, OBS, ↑).

In a classical example of a public declaration, the week before the International Day of Zero Tolerance for FGM in 2016, the President of Guinea, His Excellency Professor Alpha Conde, publicly denounced FGM for the first time. As a result, the taboo against discussing FGM publicly was broken in Guinea, and religious, community and political leaders, as well as young people, started speaking out against the practice (UNFPA, 2017; S, SR, →). Ruiz (2017) found that declarations represent a continuous, albeit gradual, rise in the awareness of the population around the problems caused by FGM (Ruiz, 2016; P, OBS, →).

In summary, regular and repeated awareness-raising interventions addressed at all sections of the society and that stress the detrimental effects of FGM, in addition to encouraging communities to make a declaration of abandonment, may be a first step towards changing attitudes and practices among community members in FGM-prevalent settings (UNICEF, 2012; P, OBS, ↑).

Table 11. Studies that assessed effectiveness of public declaration/statements

Study	Country/ region	Main objective and outcome	Key finding	Quality	Strength
UNICEF (2012)	Ethiopia	• Review of various interventions including public declaration<br>• FGM abandonment	Decrease in the number of girls cut compared with adult women after the declaration	↑	IIIb
UNFPA (2017)	Global	• Review of various interventions including public declaration<br>• FGM abandonment and attitudes	Reduced incidences of FGM – change is occurring although it appears to be slow	→	V
Ruiz et al. (2017)	Chad, Djibouti, Ghana, Mali, Morocco, the Niger and Senegal	• Assess effectiveness of public declarations in ending FGM<br>• Attitudes and awareness/knowledge	Gradual rise in awareness of consequences of FGM	→	IV
UNFPA–UNICEF (2018)	Global	• Review of various interventions including public declarations<br>• FGM abandonment and attitudes	When supported by post-declaration follow-up and support, were highly effective in preventing further FGM cases	↑	IV

Religious/cultural leaders

There were seven studies that assessed the effectiveness of using religious or other community leaders as an intervention to end FGM (*Table 12*). Five of the seven studies were of high quality, with three of these rating as Gray IIIb in strength. Four of the studies discuss the potential usefulness of religious leaders, while three provide examples of the involvement of religious leaders in declaring edicts or publicly stating their stand on FGM.

Islamic religious leaders have previously issued edicts (Fatwa) against FGM, as was the case in Egypt (Barsoum et al., 2011; P, OBS, ↑) and Mauritania (Johansen, 2013; S, OR, →). While the independent effects of such edicts are yet to be quantified, such strong positions may have some effect on behaviour and practices in communities that accord high levels of respect to religious leaders. Conversely, reluctance by religious leaders can hinder progress towards elimination of FGM (Mehari et al., 2020; P, OBS, ↑; Al-Nagar et al., 2017; S, OR, ↑).

FGM is often associated with religious and cultural obligations. Changing religion and culture to support abandonment of FGM is therefore a viable approach to contributing towards elimination of FGM (Population Reference Bureau [PRB], 2013; S; OR; ↑). In many places where FGM is practiced, traditional and religious leaders sometimes wield more power and influence than the government (Al-Nagar et al., 2017; S, OR, ↑). In such instances, religious leaders can play an important role in the elimination of FGM at the community level (Kipchumba et al., 2019; P, OBS,; PRB, 2013; S, OR, →).

In summary, religious and cultural leaders can effectively pass on messages to the community, especially in communities that are ready for change (UNICEF, 2012; P, OBS, ↑). They can be at the forefront of questioning the religious underpinnings of the practice and in publicly declaring opposition to the practice (Abdi and Askew, 2009; P; OBS; →).

Table 12. Studies that assessed effectiveness of using religious/cultural leaders

Study	County/ region	Main objective and outcome	Key finding	Quality	Strength
PRB (2013)	Global	• Review of various interventions including engagement of religious and cultural leaders to support abandonment of FGM<br>• FGM abandonment	Potentially effective approach in contributing towards elimination of FGM	→	V
Al-Nagar et al. (2017)	the Sudan	• Assess effectiveness of legislation and engaging religious and community leaders to end FGM<br>• FGM abandonment	Religious and community leaders can facilitate or hinder the fight against FGM	↑	IV
Kipchumba et al. (2019)	Somalia	• Assess effectiveness of various interventions including engaging community and religious leaders<br>• FGM prevalence, awareness and attitudes	Religious leaders have the potential to influence the outcomes of FGM interventions	↑	IIIb
UNICEF (2012)	Ethiopia	• Assess effectiveness of engaging religious leaders to end FGM<br>• FGM abandonment	Religious leaders were instrumental in supporting abandonment of FGM	↑	IIIb
Abdi and Askew (2009)	Kenya	• Assess the effect of engaging religious leaders in discussion topics on FGM<br>• Knowledge/awareness and attitudes	Some religious scholars and community members openly declared their opposition to FGM	→	V
Barsoum et al. (2011)	Egypt	• Assess effectiveness of FGM-Free Village Model that includes engaging religious leaders<br>• FGM abandonment, knowledge, and attitudes	Religious leaders were instrumental by declaring Fatwas to keep the villages FGM free	↑	IIIb
Mehari et al. (2020)	Ethiopia	• Assess community members' perspectives about the various interventions they had been exposed to, including religious approaches<br>• FGM abandonment and social norm change	The impact of religious approaches in changing norms and practices associated with FGM varies by setting	↑	IV

Conversion of traditional practitioners

Table 13 summarizes the five studies that assessed the effectiveness of using traditional practitioners to end FGM. Three studies were of moderate quality and two of high quality, although with a lower strength (Gray IV and V). Intervention activities involved either working with former traditional practitioners who had abandoned performing FGM or providing an alternative income to active traditional practitioners to encourage them to stop carrying out FGM. Evidence shows that there have been efforts to convert and provide traditional practitioners with alternative sources of income, but such efforts have not been successful. In most cases, these efforts resulted in increased medicalization of FGM (Vestbøstad and Blystad, 2014; P, OBS, →) and secrecy in conducting the practice (Buttia, 2015; P, OR, →; Van Bavel, 2020; P, OBS, ↑). Given the demand for FGM services, some communities have taken their girls to other areas or to other practitioners who can offer FGM services (Ako and Akweongo, 2009; P, OBS, ↑).

In summary, evidence suggests that while the role of traditional practitioners in the elimination of FGM cannot be underestimated, efforts to provide traditional practitioners with an alternative income have been largely ineffective (Vestbøstad and Blystad, 2014; P, OBS, →; Buttia, 2015; P, OR, →; Van Bavel, 2020; P, OBS, ↑; Ako and Akweongo, 2009; P, OBS, ↑), These efforts may need to be recalibrated if they are to be optimally used in the prevention of FGM at the community level (Johansen et al., 2013; S, OR, →).

Table 13. Studies that assessed effectiveness of conversion of traditional practitioners

Study	County/ region	Main objective and outcome	Key finding	Quality	Strength
Vestbøstad and Blystad (2014)	Somaliland	• Assess effectiveness of alternative income for traditional practitioners in ending FGM<br>• FGM abandonment and attitudes	Limited abandonment but more of changing the type of cut and medicalization	→	IV
Buttia (2015)	Kenya	• Review of various interventions including engaging former traditional practitioners<br>• FGM abandonment and knowledge/awareness	FGM performed in secret, where traditional practitioners have publicly declared support for abandonment	→	V
Van Bavel (2020)	Kenya	• Review of various interventions including engaging traditional practitioners<br>• FGM abandonment and social norm change	FGM conducted in secret, where traditional practitioners have been reached with interventions	↑	IV
Ako and Akweongo (2009)	Ghana	• Assessment of the impact of legislation, community beliefs, and engagement of traditional practitioners in ending FGM<br>• FGM abandonment	The contest between legislation and strong community beliefs have made traditional practitioners perform FGM in secret.	↑	IV
Johansen et al. (2013)	Global	• Review of various interventions including conversion of traditional practitioners<br>• FGM abandonment, knowledge, and attitudes	Limited impact in engaging traditional practitioners in the fight against FGM	→	IIIa

C. Individual level: Empowering girls and women

Interventions at the individual level include those that aim to empower girls and women to make their own informed decisions regarding their sexual and reproductive rights. Interventions assessed for effectiveness under this category were formal education for women in terms of educational attainment, and approaches that used alternative rites of passage to encourage abandonment.

Formal education

Thirteen studies evaluated the effect of formal education on the practice of FGM, seven of which were of high quality and six of moderate quality, with nine at Gray IIIa or b strength and four at IV or V (*Table 14*). Most of the studies used education as a proxy measure; however, no direct educational intervention on FGM was implemented and assessed.

Formal education may prove to be essential in reducing FGM prevalence. It has been suggested that reducing FGM requires increasing education levels among women (Ameyaw et al., 2020; P, OBS, →). Research has shown that formal education exposes girls to new information, including the health risks/consequences and illegality of FGM and can therefore play a significant role in the abandonment of the practice by empowering women and girls to reject FGM (Buttia, 2015; P, OBS, →; Berg and Denison, 2013; S, SR, ↑; Nambisia, 2014; P, OBS, →; Equality Now, 2011; S, OR, →; Van Bavel et al., 2017; P, OBS, ↑; Bø Nesje, 2014; P, OBS, ↑).

Studies have linked low levels of education to an increased likelihood of supporting and/or practising FGM. Evidence suggests that FGM is highly prevalent among girls who did not pursue formal education compared with those in school (Ameyaw et al., 2020; P, OBS, →). If a mother has higher education, her daughter is less likely to undergo FGM. Research in Kenya has shown that secondary education is associated with a fourfold increase in disapproval of FGM (Buttia, 2015; S, OR, →). In Egypt, a primary quantitative study investigated the beliefs and attitudes towards the discontinuation of FGM among ever-married (i.e., married at least once) women via an analysis of the association between different demographic characteristics of women and their attitudes towards discontinuation of FGM. The study found that a higher level of education was a strong predictor of the view that FGM should be discontinued (Afifi, 2010; P, OB, ↑). There is also a positive association between the education of the mother and a reduced risk of her daughter undergoing FGM (Modrek and Liu, 2013; P, OBS, →). However, another study in Egypt found no significant difference in attitudes towards FGM between more- and less-educated families (Hassanin and Shaaban, 2013; P; OBS; →).

A study conducted among college students in Northern and Southern Sierra Leone found that the educational attainment of college students did not have a significant influence on their hegemonic attitudes or their attitude towards FGM (Small et al., 2019; P, OBS, ↑). However, parental educational

attainment was associated with the attitudes of both male and female students towards FGM (Small et al., 2019; P, OBS, ↑).

One review found a paucity of high-quality evidence regarding the effectiveness of interventions to prevent FGM and concluded that the evidence base was insufficient to draw solid conclusions (Denison et al., 2009; S, SR, ↑). However, the evidence suggests that educating female students may have led to a small increase in knowledge/awareness about FGM. The authors note that the low quality of the body of evidence affected the interpretation of results and raised doubts about the validity of the findings (Denison et al., 2009; S, SR, ↑).

In summary, evidence shows that formal education is effective in reducing FGM prevalence (Ameyaw et al., 2020; P, OBS, →). Education plays a significant role in empowering women and girls to demand their rights and to challenge existing gender and social inequalities such as FGM (Buttia, 2015; P, OBS, →; Berg and Denison, 2013; S, SR, ↑; Nambisia, 2014; P, OBS, →; Rawat, 2017; P, OBS, ↑).

Table 14. Studies that assessed effectiveness of formal education

Study	Country/ region	Main objective and outcome	Key finding	Quality	Strength
Equality Now (2011)	Kenya and the United Republic of Tanzania	• Review of various interventions including promotion of girls' education<br>• FGM abandonment	Girls empowered to say no to FGM	→	IV
Hassanin and Shaaban (2013)	Egypt	• Assess effectiveness of legislation and formal education on FGM<br>• FGM abandonment, attitudes and medicalization	Little change in attitude among educated families	→	IIIa
Rawat (2017)	Burkina Faso, Chad, Guinea, Mali, Sierra Leone, and Somalia	• Assess the impact of education and economic development on FGM<br>• FGM abandonment	Higher levels of education for women were associated with lower levels of FGM	↑	IV
Berg and Denison (2013)	Burkina Faso, Ethiopia, Kenya, Mali, Nigeria, Senegal	• Systematic review of various interventions including formal education<br>• FGM abandonment, knowledge and attitudes	Education of female students increased students' knowledge of the likely complications of FGM	↑	IIIa
Van Bavel et al. (2017)	The United Republic of Tanzania	• Assess effectiveness of multiple interventions including formal education<br>• FGM abandonment and attitudes	Changing attitudes towards FGM among the younger generation due to education	↑	V
Denison et al. (2009)	Global	• Systematic review of various interventions including formal education<br>• FGM abandonment, knowledge/ awareness, and attitudes/beliefs	Educating female students may have led to a small increase in knowledge/ awareness about FGM	↑	IIIa

Study	Country/ region	Main objective and outcome	Key finding	Quality	Strength
Nambisia (2014)	Kenya	• Review of various interventions including promotion of girl child education<br>• FGM abandonment and attitudes	Girl-child education had significantly contributed to the eradication of FGM among the Maasai.	→	IIIb
Small et al. (2020)	Sierra Leone	• Assess the influence of students' and parents' formal education on attitudes towards FGM<br>• Attitudes	Education did not change the atti-tudes of students towards FGM. However, parental education had an influence	↑	IIIb
Ameyaw et al. (2020)	Sierra Leone	• Assess the effect of education among women and intention to cut daughters<br>• Attitudes	Women who had no formal educa-tion were more likely to intend to cut their daughters	→	IIIb
Buttia (2015)	Kenya	• Review of various interventions including promotion of education of girls<br>• FGM abandonment and knowl-edge/awareness	Successful where interventions were integrated. FGM was also increas-ingly performed in secret	→	V
Modrek and Liu (2013)	Egypt	• Assessment of multiple inter-ventions including maternal education on FGM<br>• FGM abandonment	Increases in wom-en's education may be causally related to the reduction in FGM prevalence	→	IIIb
Bø Nesje (2014)	Kenya	• Assess the effect of educating mothers on the prevalence of FGM of their eldest daughters<br>• FGM abandonment	Additional year of schooling led to a decrease in the likelihood of FGM among daughters	↑	IIIb
Afifi (2009)	Egypt	• Assess the influence of women's empowerment and intention to continue practising FGM<br>• Attitudes	Women with high levels of em-powerment and education were less likely to intend to perpetuate FGM for their daughters	↑	IIIb

Alternative rites of passage

Four high- and six moderate-quality studies assessed the effectiveness of alternative rites of passage (ARP) as a strategy to end FGM (the strength of these studies was relatively weak, with seven rating as Gray IV or V and only three as IIIa or b) (*Table 15*). Alternative rites of passage allow girls to undergo training and to graduate to womanhood without being subjected to FGM. During this process, girls are also educated on different topics, such as human rights and the adverse effects of FGM, and are encouraged to abandon the practice (Buttia, 2015; S, SR, →). Among groups that had dedicated training on the harmful effects of FGM, ARP increased reproductive health knowledge. In some instances, while the training did not necessarily lead to the abandonment of FGM, changes in the practice were reported – from severe to less severe cuts, for example (Mepukori, 2016; P, OBS, ↑).

Nambisia (2014) found that most respondents supported public ceremonies where girls were celebrated as they entered womanhood without necessarily going through FGM (Nambisia, 2014; P, OBS, →). The detaching of FGM from the ceremony offered community members willing to continue the practice the opportunity to do so without being prosecuted, while at the same time providing those unwilling to undergo or perform the practice the opportunity to evade FGM without being socially condemned (Van Bavel et al., 2017; P, OBS, ↑).

Alternative rites facilitate community ownership and support, as they maintain key cultural practices, increase knowledge and empowerment of girls, and increase publicity about change through community celebrations (Mepukori, 2016; P, OBS, ↑). However, this approach is only viable in communities where FGM is part of a rite of passage (Johansen et al., 2013; S, OR, →). A study by UNICEF (2010) revealed a limited impact of ARP in Kenya. Here, the success of ARP has been curtailed by a trend in some communities where FGM is increasingly being performed at younger ages and with less ceremony and ritual. Generally, ARP adopted in isolation does not address the underlying social values associated with FGM. Therefore, the social stigma of not undergoing FGM remains and girls continue to be pressured to undergo the practice (UNICEF Innocenti Insight, 2010; P, OBS, →).

Mepukori (2016) examined the effectiveness of ARP as a single intervention among the Samburu community in Kenya and found no reduction in FGM when ARP was implemented in isolation (Mepukori, 2016; P, OBS, ↑). One of the recommendations from the study was that for ARP to be effective, it should be implemented in combination with other intervention approaches such as community awareness-raising initiatives. Oloo et al. (2011) similarly contend that ARP that involves intensive community sensitization about FGM, combined with a public declaration ceremony and fully integrated into a girls' empowerment programme, can be effective in encouraging abandonment of FGM in rural communities (Oloo et al., 2011; P, OBS, ↑).

In summary, while some components of ARP, such as educational programmes, can be effective in changing attitudes (Abreu and Abreu, 2014; P, OBS, →), the risk of exclusion, perceived loss of cultural identity, changing meanings ascribed to cultural practices, lack of precise knowledge about subjective sexual experience and negative stereotyping limit the success of such programmes (Graamans et al., 2019; P, OBS; →). Mwendwa and colleagues (2020) argue that effective ARP will require a combination of behaviour change support at the community level, law enforcement and monitoring, and open and persistent advocacy by diverse representatives of communities (Mwendwa et al., 2020; P, OBS, ↑).

Table 15. Studies that assessed effectiveness of alternative rites of passage

Study	Country/region	Main objective and outcome	Key finding	Quality	Strength
Buttia (2015)	Kenya	• Review of various interventions including alternative rites of passage (ARP)<br>• FGM abandonment and knowledge/awareness	Reduced prevalence of FGM. ARP considered effective in influencing girls to reject FGM	→	V
Mepukori (2016)	Kenya	• Assess encouragement/training of communities to maintain the cultural ceremonies and rites surrounding female initiation whilst getting rid of FGM<br>• FGM abandonment, knowledge and attitudes	Some change in attitudes. Community disagreements with some of the approaches	↑	V
Abreu and Abreu (2014)	Portugal	• Assess education programme as a form of alternative ritual to FGM<br>• FGM abandonment	Accepted by the population and contributed to the reduction of FGM	→	V
Oloo et al. (2011)	Kenya	• Assess effectiveness of ARP focusing on community sensitization, training on family life education, and public graduation ceremony<br>• FGM abandonment	Reduction in FGM prevalence but context specific: positive changes in Kisii but not in Kuria	↑	IIIb
Graamans et al. (2019)	Kenya	• Assess Amref Health Africa's efforts to end FGM through ARP<br>• FGM abandonment	Limited effectiveness. Risk of exclusion, perceived loss of cultural identity, changing meanings ascribed to cultural practices, lack of precise knowledge about subjective (sexual) experience and negative stereotyping	→	V
Van Bavel et al. (2017)	the United Republic of Tanzania	• Assess effectiveness of ARP through education<br>• FGM abandonment and attitudes	Changed attitudes towards FGM among the younger generation. The practice is more often done secretly.	↑	V
Nambisia (2014)	Kenya	• Review various interventions including alternative rites of passage<br>• FGM abandonment and attitudes	ARP considered a vital FGM eradication measure. Public ceremonies held to celebrate girl's entry into womanhood without FGM	→	IIIb
Mwendwa et al. (2020)	Kenya	• Assess perceived effectiveness of FGM interventions including ARP<br>• FGM abandonment and attitudes	Substantial shift in attitude towards FGM with decrease in FGM prevalence	↑	IV
UNICEF Innocenti Insight (2010)	Egypt, Ethiopia, Kenya, Senegal and the Sudan	• Review various interventions including ARP in Kenya<br>• FGM abandonment and attitudes	Limited impact. Curtailed by communities cutting girls at younger ages with less ceremony and ritual	→	IV
Johansen et al. (2013)	Burkina Faso, Egypt, the Gambia, Guinea-Bissau, Indonesia, Kenya, Mali, Mauritania, Senegal, Sierra Leone, Somalia and the Sudan	• Review various interventions including ARP<br>• FGM abandonment, knowledge, and attitudes	Increased knowledge and change in attitude among girls. Change in attitude among parents towards FGM, reduced intention to subject daughters to FGM, increased knowledge about consequences of FGM.	→	IIIa

D. Service level: Services for FGM prevention, protection and care

Service-level interventions aim to protect girls and women at risk of FGM, prevent FGM, and provide care to women and girls who have undergone FGM. Interventions assessed for effectiveness in this review include training health-care providers and capacity-building of the health system, and the use of rescue centres at the community level.

Training health-care providers/capacity-building of the health-care system

Six studies assessed the effect of training providers/enhancing the health-care system, four of which were of high quality and two of moderate quality, with four at Gray rating of IIIa or b and two at Gray IV (*Table 16*). Most of the interventions focused on imparting knowledge and skills to health-care providers, either to act as agents of change in the prevention of FGM or to offer better services to clients seeking health services post-FGM. Evidence shows that training can be effective in imparting knowledge to health-care workers (Kimani et al., 2018; P, OBS, →). Kimani and colleagues (2018) found that comprehensive knowledge and competencies on FGM among providers was critical for rejecting/preventing the practice. This was experienced after the training of health-care providers using an online UNFPA training module (Kimani et al., 2018; P, OBS, →). In contrast, systematic reviews by Berg (2013) and Denison et al. (2009) suggested that training health personnel did not change their knowledge and beliefs/attitudes regarding FGM (Berg, 2013; S, SR, ↑; Denison et al., 2009; S, SR, ↑). The authors suggest that such training may need to be accompanied by additional interventions aimed at offering comprehensive competencies to health-care providers. In Mali, even after training, fewer providers wished to play a role in educating clients about the practice and a sense of advocacy among participating health personnel appeared weak (Berg, 2013; S, SR, ↑; Denison et al., 2009; S, SR, ↑).

A study conducted in the United Kingdom showed that training of health-care providers can enhance their skills in managing complications related to FGM in health-care settings (McCracken, 2017; P, OBS, ↑). Similar findings have been observed in Kenya, where training changed the attitude of health-care workers towards FGM and individuals who experienced FGM (Kimani et al., 2018; P, OBS, →). Training in certain circumstances can also assist providers in evaluating the risk of exposure to FGM (Balfour et al., 2016; S, OR, →).

In summary, although there is limited evidence on the effectiveness of interventions at the service level, available evidence shows that training health-care providers can improve their knowledge and skills to act as agents of change in the prevention of FGM and offer quality services to clients seeking care post-FGM (Kimani et al., 2018; P, OBS, →; McCracken, 2017; P, OBS, ↑). An improved health-care system should therefore have the capacity to manage and provide optimal services to clients who have undergone FGM and prevent the practice from occurring among those at risk (Kimani and Okondo, 2020; P, OBS, ↑).

Table 16. Studies that assessed effectiveness of training of health-care providers/capacity-building of the health-care system

Study	Country/ region	Main objective and outcome	Key finding	Quality	Strength
McCracken (2017)	England	• Assess the quality of prevention, care and support services for women that have undergone FGM<br>• Quality of care and attitudes	Change in attitude towards FGM; increase in specialized care and support; increased open discussion on FGM; and improved services and service delivery	↑	IIIb
Kimani et al. (2018)	Kenya	• Assess effectiveness of training nurse-midwives using an electronic tool derived from a paper-based quiz on FGM<br>• Knowledge and quality of care	Substantial FGM-related knowledge demonstrated by nurse-midwives. Challenges in preventing medicalization with knowledge gaps concerning sexual and social complications.	→	IIIb
Kimani and Okondo (2020)	Kenya	• Assess the health system's capacity to handle FGM<br>• FGM prevention and quality of care	Although the legal and policy instruments are well understood by policy actors at the national and county level, few health care providers are aware of these instruments. The limited awareness has a negative impact on the quality of care received by women and girls.	↑	IV
Balfour et al. (2016)	Global	• Systematic review of interventions aimed at improving health-care providers' capacities to prevent and treat FGM-related complications<br>• Knowledge, attitudes and quality of care	Two studies reported improvement of health-care professionals' knowledge and attitude towards FGM and confidence in clinical management. Noted weak evidence making it difficult to assess effectiveness of training on providers.	↑	IV
Berg and Denison (2013)	Burkina Faso, Ethiopia, Kenya, Mali, Nigeria, Senegal	• Review of various interventions including training of health personnel<br>• FGM abandonment, knowledge, and attitudes	No significant difference between the intervention and comparison groups regarding any outcome	↑	IIIa
Denison et al. (2009)	Burkina Faso, Ethiopia, Kenya, Mali, Nigeria, Senegal	• Review of various interventions including training of health personnel<br>• FGM abandonment, knowledge/ awareness, and attitudes/beliefs	Training health personnel likely produced no effects in knowledge or beliefs/ attitudes about FGM. Notes weak evidence base.	↑	IIIa

Rescue centres

Five studies assessed the effectiveness of rescue centres as an approach to end FGM. Four of these were of moderate quality and one of high quality, with one rated as Gray IIIb and the remainder Gray IV or V (*Table 17*). Notably, most of the studies included rescue centres among other interventions, with limited information on the assessment of rescue centres as an independent intervention.

Rescue centres or safe houses aim to provide protection and refuge for girls who are at risk of FGM during the cutting period. Apart from providing shelter to girls running away from FGM, rescue centres also educate girls on the health risks and illegality of FGM, and its violation of human rights (Nambisia, 2014; P, OBS, →). Research has shown that rescue centres face challenges such as limited resources and lack of recognition and buy-in of the intervention by the community and there is therefore limited evidence on their effectiveness (Buttia, 2015; S, OR, →).

In Kenya and the United Republic of Tanzania, the evaluation of a project that used a mix of interventions, including safe houses/rescue centres, to protect girls from FGM found that their sensitization campaigns were successful because they empowered girls to say no to the practice, with many seeking refuge in safe houses or reporting attempts to be cut to the police (Equality Now, 2011; P, OBS, →). A publication on lessons learned from a decade of progress in eradicating FGM suggested that the use of safe houses can be problematic, as this approach may merely remove the girl or woman from the situation rather than address the social norms and pressures driving the practice (PRB, 2013; P, OBS, →). A review of interventions including safe houses among the Maasai, Kisii and Kuria communities in Kenya suggested that rescue centres can be successful if integrated with other interventions to eradicate FGM (Buttia, 2015; S, OR, →).

In summary, there is limited evidence on the effectiveness of rescue centres in ending FGM. The few available studies show that rescue centres can provide short-term refuge for girls at risk of FGM (Nambisia, 2014; P, OBS, →) but are limited in providing long-term solutions to ending the practice (Buttia, 2015; S, OR, →; PRB, 2013; P, OBS, →).

Table 17. Studies that assessed effectiveness of rescue centres

Study	County/ region	Main objective and outcome	Key finding	Quality	Strength
Equality Now (2011)	Kenya and the United Republic of Tanzania	• Review various interventions including rescue centres<br>• FGM abandonment	Girls have been empowered to say no to FGM	→	IV
Population Reference Bureau (PRB) (2013)	Global	• Review various interventions including safe houses<br>• FGM abandonment	Safe houses may merely remove the girl or woman from the situation rather than address the social norms and pressures driving the practice	→	V
Van Bavel (2020)	Kenya	• Reviewed various interventions including rescue centres<br>• FGM abandonment and social norm change	Limited effectiveness of rescue centres	↑	IV
Nambisia (2014)	Kenya	• Assess effectiveness of multiple interventions including rescue centres<br>• FGM abandonment and attitudes	Challenges with reconciliatory efforts between the rescued girls and their parents	→	IIIb
Buttia (2015)	Kenya	• Review of various interventions including rescue camps<br>• FGM abandonment and knowledge/awareness	Limited impact when implemented in isolation	→	V

What works in FGM prevention and response?

Combining the Gray rating of the moderate and high-quality studies with the geographical spread of the interventions allows for analysis of successful programming towards abandoning FGM. This evidence review has demonstrated that there are some interventions that could lead to the abandonment of FGM. However, given the limited evidence across countries and regions overall, it is difficult to make strong claims about interventions that may be said to 'work,' particularly in varying cultural contexts. Nonetheless, a review of the evidence using the following categorization elicits several promising interventions with sufficient indications for additional action:

Successful interventions with supporting evidence:

Four or more studies that are Gray IIIb or higher (e.g., IIIa/b, II, I; *see Table 5 for a description of Gray categories*) and have evidence from more than one country.

Promising interventions that need further evidence:

Three or fewer studies of any Gray level or two or more studies at Gray IIIb or higher but from only one country.

Interventions that do not work:

Four or more studies that are Gray IIIb or higher (i.e., IIIa/b, II, I) and have evidence from more than one country that the intervention does not work.

See Table 18 for a summary of the interventions in each of the three categories.

Successful interventions with supporting evidence

While there is insufficient evidence to identify successful programming at the system level, a body of evidence exists on intermediate outcomes to behavioural change at the community and individual levels. Given the collective strength of evidence, this data on intermediate outcomes may be used to suggest interventions worthy of implementation on a wider scale.

At the community level, a body of evidence demonstrates that health education and community dialogues with parents and religious leaders can change attitudes about FGM; this is an important step in the continuum of change towards abandonment of FGM. Evidence for this intervention is based on 10 studies (Diop and Askew, 2009; Johansen et al., 2013; Abdulah et al., 2020; Asekun-Olarinmoye and Amusan, 2008; Ekwueme et al., 2010; Galukande et al., 2019; Kipchumba et al., 2019; Barrett et al., 2020; UNICEF, 2010; Brown, 2013), of which 7 are rated Gray IIIa or b, 2 are Gray IV and 3 are Gray V (*see Table 5 for the full description of Gray categories*).

Media and social marketing can be effective in changing social norms and attitudes towards abandoning FGM, and, in some cases, reducing FGM. This is based on 13 studies (Abathun et al., 2018; Berg and Denison, 2013; Evans et al., 2019; Ahmed, 2012; Hussein and Ghattass, 2019; Kaunga, 2014; Suzuki and Meekers; 2008; Vogt et al., 2016; Mehari et al., 2020; Brown, 2013; Buttia, 2015; Nielssen and Coulibaly, 2014; UNFPA, 2017), 8 of which were a Gray IIIa or b, 1 Gray IV and 4 Gray V. Collectively, these studies show that media and social marketing can be useful tools in addressing norms around FGM.

At the individual level, educating mothers may reduce the numbers of girls undergoing FGM: the higher the level of formal education of a mother, the less likely her daughter was to undergo the procedure. Five studies in this review – four Gray IIIb and one Gray IV – show that a mother's formal education has a positive effect on her daughter (Afifi, 2010; Ameyaw et al., 2020; Bø Nesje, 2014; Modrek and Liu, 2013; Rawat, 2017).

Four further studies – three Gray IIIa/b and one Gray IV – showed that educating girls leads to improved knowledge and changing attitudes, an important step in the continuum of change towards abandonment of FGM (Denison et al., 2009; Berg and Denison, 2013; Nambisia, 2014; Van Bavel et al., 2017).

Together, these interventions have a strong enough body of evidence to justify wider implementation as part of comprehensive efforts to abandon FGM.

Promising interventions that need further evidence

Some interventions are promising despite a supporting body of evidence with more limited strength. These require additional, more rigorous studies before they could be considered 'successful' (in the same way as the interventions described above). Nevertheless, these studies suggest promising interventions at the system, community and service levels. For example, at the system level, seven studies – one Gray IIIb, five Gray IV and one Gray V – suggest that legislation accompanied by political will, in combination with additional interventions such as sensitization and locally appropriate enforcement mechanisms, hold promise for reducing FGM (Kandala and Komba, 2015; Ako and Akweongo, 2009; Al-Nagar et al., 2017; Baillot et al., 2018; Mehari et al., 2020; Muthumbi et al., 2015; Nabaneh and Muula, 2019).

At the community level, four studies – one Gray IIIb, two Gray IV and one Gray V – show that creating FGM-free communities via public declarations, particularly when accompanied by post-declaration follow-up, may change attitudes and potentially reduce FGM (UNICEF, 2012; Ruiz et al., 2017; UNFPA–UNICEF, 2018; UNFPA, 2017). Another seven studies – three Gray IIIb, two Gray IV, and two Gray V – found that public statements of opposition to FGM by religious leaders may help to change attitudes towards abandoning FGM (Barsoum et al., 2011; Kipchumba et al., 2019; UNICEF, 2012; Al-Nagar et al., 2017; Mehari et al., 2020; Abdi and Askew, 2009; PRB, 2013).

At the service level, two Gray IIIb studies suggest that training health-care providers can improve capacity for prevention and treatment of FGM (Kimani et al., 2018; McCracken, 2017).

These promising interventions, along with the 'successful' interventions above with a stronger evidence base, provide eight potential interventions – with supporting evidence – that could guide policy and programming towards the abandonment of FGM.

Interventions lacking evidence

It is critically important to identify interventions that research shows to be ineffective and/or harmful to avoid or discontinue allocating resources towards these efforts. This review finds that the current body of evidence does not show effectiveness of these interventions at the system, community or individual levels.

At the system level, while legislation can be effective when combined with other interventions, 11 studies – 6 Gray IIIa/b, 4 Gray IV and 1 Gray V – show that legislation may take a long time to end FGM as countries usually pass laws as a precursor to enforcement (Cetorelli et al., 2020; Hassanin and Shaaban, 2013; Camilotti, 2016; Hassanin et al., 2008; Kandala and Komba, 2015; Nambisia, 2014; Al-Nagar et al., 2017; Brown and Porter, 2016; Meroka-Mutua et al., 2020; Wouango et al., 2020; Dowuona-Hammond et al., 2020). An additional six studies – one Gray IIIa, three Gray IV and two Gray V – find that criminalization may drive FGM underground, thus further entrenching the practice (Shell-Duncan et al., 2013; Ako and Akweongo, 2009; Boyden, 2012; Plugge et al., 2019; Buttia, 2015; Johnsdotter, 2019).

At the community level, five studies – one Gray IIIa, three Gray IV and one Gray V – find that efforts to convert and/or provide traditional practitioners with alternative sources of income have not been effective in eliminating FGM (Johansen et al., 2013; Ako and Akweongo, 2009; Van Bavel, 2020; Vestbøstad and Blystad, 2014; Buttia, 2015).

A further five studies at the individual level – one Gray IIIb, two Gray IV and two Gray V – suggest that alternative rites of passage with a focus on the public ceremonial passage of girls is not effective in reducing or eliminating FGM (Oloo et al., 2011; Mwendwa et al., 2020; UNICEF, 2010; Mepukori, 2016; Graamans et al., 2019).

The collective strength of these moderate- and high-quality studies identifies successful and promising interventions, as well as interventions where evidence on their effectiveness is lacking, and thus provides ideas for guiding potential programming and policymaking.

Successful interventions with supporting evidence

Table 18. Summary of interventions that are successful, promising, or lack evidence

Level	Intervention/evidence
Community	Health education and community dialogues with parents and religious leaders can change attitudes about FGM: an important step in the continuum of change towards abandonment of FGM (Diop and Askew, 2009, Gray IIIa; Johansen et al., 2013, Gray IIIa–IV; Abdulah et al., 2020, Gray IIIb; Asekun-Olarinmoye and Amusan, 2008, Gray IIIb; Ekwueme et al., 2010, Gray IIIb; Galukande et al., 2019, Gray IIIb; Kipchumba et al., 2019, Gray IIIb; Barrett et al., 2020, Gray IV; UNICEF, 2010, Gray IV; Brown, 2013, Gray V).<br><br>Media and social marketing efforts are effective in changing social norms and attitudes towards abandoning FGM, and, in some cases, reducing FGM (Abathun et al., 2018, Gray IIIa; Berg and Denison, 2013, Gray IIIa–IV; Evans et al., 2019, Gray IIIa; Ahmed, 2012, Gray IIIb; Hussein and Ghattass, 2019, Gray IIIb; Kaunga, 2014, Gray IIIb; Suzuki and Meekers, 2008, Gray IIIb; Vogt et al., 2016, Gray IIIb; Mehari et al., 2020, Gray IV; Brown, 2013, Gray V; Buttia, 2015, Gray V; Nielssen and Coulibaly 2014, Gray V; UNFPA, 2017, Gray V).
Individual	Educating mothers can reduce the numbers of girls undergoing FGM. The higher the level of formal education of a mother, the less likely her daughter is to undergo FGM (Afifi, 2010, Gray IIIb; Ameyaw et al., 2020, Gray IIIb; Bø Nesje, 2014, Gray IIIb; Modrek and Liu, 2013, Gray IIIb; Rawat, 2017, Gray IV).<br><br>Educating girls leads to improved knowledge and changing attitudes: an important step in the continuum of change towards abandonment of FGM (Denison et al., 2009, Gray IIIa; Mahgoub et al., 2019, IIIa; Berg and Denison 2013, Gray IIIa-IV; Nambisia, 2014, Gray IIIb; Van Bavel et al., 2017, Gray V).

Promising interventions that need further evidence

Level	Intervention/evidence
System	Legislation accompanied by political will, in combination with additional interventions such as sensitization and locally appropriate enforcement mechanisms are promising practices in reducing FGM (Kandala and Komba, 2015, Gray IIIb; Ako and Akweongo, 2009, Gray IV; Al-Nagar et al., 2017, Gray IV; Baillot et al.,2018, Gray IV; Mehari et al., 2020, Gray IV; Muthumbi et al., 2015, Gray IV; Nabaneh and Muula, 2019, Gray V).
Community	Creating FGM-free communities via public declarations, particularly when accompanied by post-declaration follow up, may change attitudes and potentially reduce FGM (UNICEF, 2012, Gray IIIb; Ruiz et al., 2017, Gray IV; UNFPA–UNICEF, 2018, Gray IV; UNFPA, 2017, Gray V).<br><br>Public statements of opposition to FGM by religious leaders may help change attitudes towards abandoning FGM (Barsoum et al., 2011, Gray IIIb; Kipchumba et al., 2019, Gray IIIb; UNICEF, 2012, Gray IIIb; Al-Nagar et al., 2017, Gray IV; Mehari et al., 2020, Gray IV; Abdi and Askew, 2009, Gray V; PRB, 2013, Gray V).
Service	Health-care provider training can improve capacity for prevention and treatment of FGM. Further information is needed on the type of training and the best ways to address the gaps (Kimani et al., 2018, Gray IIIb; McCracken, 2017, Gray IIIb).

Interventions lacking evidence

Level	Intervention/evidence
**System**	Legislation may take a long time to end FGM (Cetorelli et al., 2020, Gray IIIa; Hassanin and Shaaban, 2013, Gray IIIa; Camilotti, 2016, Gray IIIb; Hassanin et al., 2008, Gray IIIb; Kandala and Komba, 2015, Gray IIIb; Nambisia, 2014, Gray IIIb; Al-Nagar et al., 2017, Gray IV; Brown and Porter, 2016, Gray IV; Meroka-Mutua et al., 2020, Gray IV; Wouango et al., 2020, Gray IV; Dowuona-Hammond et al., 2020, Gray V); additionally, criminalization may drive the practice underground (Shell-Duncan et al., 2013, Gray IIIa; Ako and Akweongo, 2009, Gray IV; Boyden, 2012, Gray IV; Plugge et al., 2019, Gray IV; Buttia, 2015, Gray V; Johnsdotter, 2019, Gray V).
**Community**	Efforts to convert and/or provide traditional practitioners with alternative sources of income have not been effective in eliminating FGM (Johansen et al., 2013, Gray IIIa; Ako and Akweongo, 2009, Gray IV; Van Bavel, 2020, Gray IV; Vestbøstad and Blystad, 2014, Gray IV; Buttia, 2015, Gray V).
**Individual**	Alternative rites of passage with a focus on the public ceremonial passage of girls is not effective in reducing or eliminating FGM (Oloo et al., 2011, Gray IIIb; Mwendwa et al., 2020, Gray IV; UNICEF, 2010, Gray IV; Mepukori, 2016, Gray V; Graamans et al., 2019, Gray V).

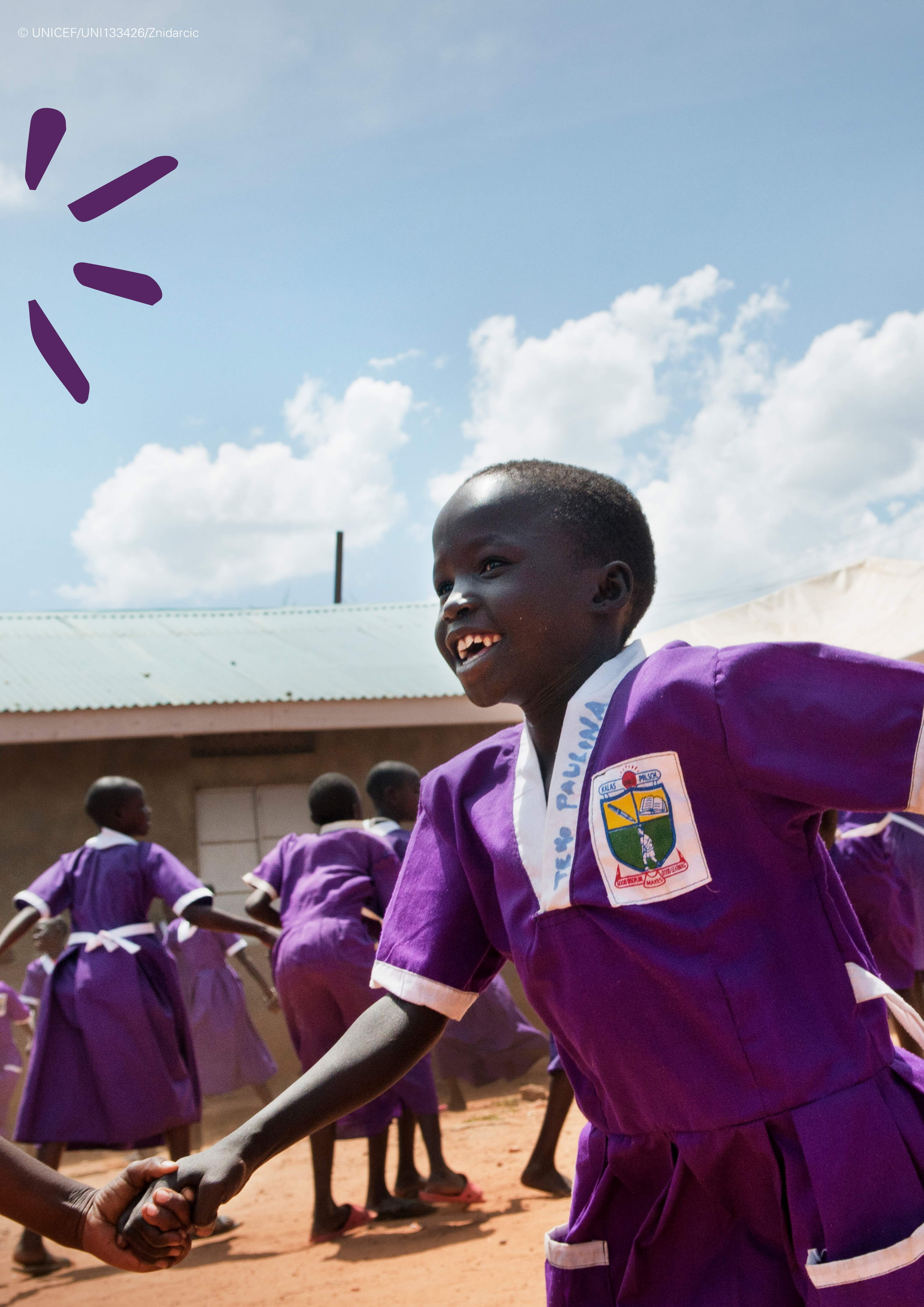

© UNICEF/UNI133426/Znidarcic

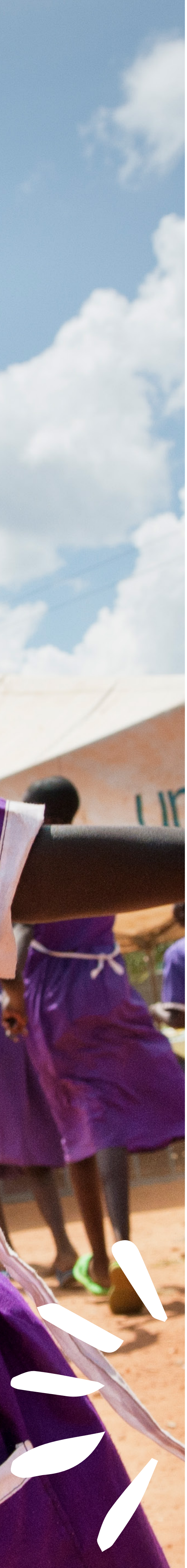

DISCUSSION

System level: Enabling environment for ending FGM
Community level: Addressing gender inequalities and social norms
Individual level: Empowering girls and women
Service level: Services for FGM prevention, protection and care
A holistic and multisectoral approach to FGM interventions
Areas for further research
Gaps and study limitations

This review assessed the quality and strength of evidence of studies that evaluated interventions for the prevention of and response to FGM. The review also aimed to determine the most promising interventions for the prevention of and response to FGM, based on the existing evidence. There has been an increase in the number of interventions that have been assessed for their effectiveness in the prevention of and response to FGM between 2008 and 2020. This trend signals an increase in investments towards the elimination of FGM, especially in Africa, where most of the reviewed interventions were implemented, and an increased commitment to generating evidence on what works. Despite this notable increase, however, most of the studies were observational; few experimental studies have assessed the effectiveness of FGM interventions. This highlights the need for the application of robust study designs and analytical techniques that can clearly demonstrate the effectiveness (cause and effect) of FGM interventions on FGM abandonment and intermediate outcomes.

The discussion below reflects on the key findings from the review based on the four broad thematic areas around which the results were organized: system, community, individual and service levels.

System level: Enabling environment for ending FGM

The system-level studies focused on assessing the effectiveness of national anti-FGM legislation in ending the practice. Findings from the review suggest that, while laws against FGM legitimize other programmatic interventions, its success in ending FGM may take longer. Evidence showed that enforcement of legislation without consideration of the contextual factors that drive FGM can be counterproductive. In high income countries, the law has sometimes alienated the intended beneficiaries seeking care in health-care facilities and compromised reporting. Women or girls may also not seek health care for fear of criminalization or stigma. In Africa, enforcement of laws sometimes led to modifications in how FGM is practised, such as the reduction in the age of undergoing FGM, medicalization and carrying out the practice in secret. Nevertheless, laws criminalizing FGM are important for creating an enabling environment for programmes implementing interventions to end the practice and in offering support to those who are contemplating or have already abandoned the practice and can also act as a deterrent.

At the system level, the key message is that the way anti-FGM laws are enforced and the existing context play a significant role in determining success. For example, when anti-FGM laws are introduced in contexts where social norms associated with FGM are stronger, community members are likely to weigh social and legal consequences, with the result being the continuation of the practice in secret to avoid prosecution (Meroka-Mutua et al., 2020; Wouango et al., 2020). It is therefore vital that anti-FGM laws are developed, introduced and implemented in ways that embrace the engagement of community members in the transformation of social norms. Findings from this review suggest that for anti-FGM legislation to provide a conducive environment for effective implementation of interventions, political will, the existence of locally appropriate and sufficiently resourced enforcement mechanisms, and proper sensitization about the law are paramount. As laws are punitive in nature, their imple-

mentation should be accompanied by other interventions that target the drivers of FGM, including changing attitudes and norms that support the continuation of the practice.

This review shows that studies at the system level solely focused on national FGM legislation. However, other interventions at the system level exist, such as mainstreaming FGM in school curricula, social protection programmes targeting girls and women, and provision of legal, social and health services for prevention and response to FGM. These interventions must be multifaceted to be effective.

The review also shows that there is a research gap for other kinds of system interventions. There is limited evidence on how policies and legislation related to FGM have been integrated with other relevant policies and legislation, such as child protection and health services. For example, integrated legislation would allow for coordinated action through child protection and sexual and reproductive health services; make it easy to identify, report, refer and support girls and women at risk of undergoing FGM or those seeking care; and ensure that there is effective linkage among the different stakeholders, especially law enforcement agencies, programme implementers and community members (UNFPA–UNICEF, 2017).

Community level: Addressing gender inequalities and social norms

At the community level, community engagement using a holistic approach of education and empowerment programmes was the most promising intervention approach in encouraging FGM abandonment. Although the approach has shown mixed results across the different settings where it has been tested, it signifies the importance of context when designing FGM interventions. It also highlights the need for meaningful involvement of the community in designing FGM interventions and for taking a broader approach that addresses the wider challenges affecting the community. Health education approaches and the use of media/social marketing campaigns/communication initiatives to end FGM showed positive effects in imparting knowledge related to the physical, psychological and emotional consequences of FGM and changing beliefs and attitudes towards the practice. Nonetheless, there was no evidence found on the impact of these two interventions in changing behaviour.

Evidence on the pathway of change from community members being knowledgeable and against FGM to abandonment of the practice was limited. Few studies assessed the effectiveness of public declarations/statements and the use of religious/cultural leaders to facilitate change towards FGM abandonment. Nonetheless, the few available studies describe these approaches as potentially effective in influencing beliefs and attitudes towards FGM as a precursor to change. However, evidence on the use of traditional practitioners as change agents or providing traditional practitioners with alternative income so that they can stop providing FGM services showed that such approaches do not work.

At the heart of community-level interventions is the appreciation that FGM is driven by gender and social norms that involve social pressure on

community members to conform. The need to be socially accepted by community members and the fear of the consequences of not conforming are strong motivations that can sustain FGM (Mackie and LeJeune, 2009; Shell-Duncan et al., 2011). This is salient in communities where FGM is almost universally practised. In such a context, interventions to shift existing gender and social norms are likely to be successful in changing behaviour if they target social groups and networks (Cislaghi and Heise, 2018a; Shell-Duncan et al., 2018).

This study highlights the need for more emphasis on attitudinal and collective behavioural change approaches when implementing FGM interventions. The findings also reveal a lack of explicitness in the studies regarding how the issue of gender was covered. Some stakeholders may be unaware of the gender and social norms approaches, demonstrating the need for capacity-building and working with multiple stakeholders within a community as gatekeepers of social norms and gender equality (UNFPA–UNICEF, 2017).

Individual level: Empowering girls and women

At the individual level, studies investigated the effectiveness of women's educational attainment and the use of alternative rites of passage (ARP) in ending FGM. These interventions aimed to empower girls and women to make their own informed decisions regarding their sexual and reproductive rights, including resisting FGM. Evidence from this review suggests that formal education for women/girls is effective in protecting girls from FGM by keeping them in school and empowering them with information to reject FGM. Maternal educational attainment is also effective in influencing mothers' attitudes towards FGM abandonment and decisions not to subject their daughters to the practice. However, FGM is complex and relying solely on education for women and girls may not result in complete abandonment as quickly as needed. Formal education should therefore be complemented with comprehensive community-led strategies that challenge existing gender and social norms that propagate FGM. While girls' formal education programmes are implemented in the long-term by government and private institutions and not necessarily as a direct intervention in ending FGM, there is a greater benefit for programmes to partner with and support interventions that ensure girls remain in school and are empowered to act as agents of change.

This review shows that while the activities associated with ARP, such as training on the harmful effects of FGM, may lead to increased knowledge, there is limited evidence on the effectiveness of ARP in preventing girls from undergoing FGM. The approach is only relevant in communities that consider FGM to be a rite of passage. Another significant limitation of the ARP approach is the observed changes in how FGM is being practised: increasingly, FGM is being performed at younger ages with less ceremony. This implies that ARP may not achieve the desired outcome of promoting the cultural ceremony of girls coming of age without undergoing FGM, as the procedure could have happened discreetly at a much younger age. Importantly, evidence from Kenya has shown that ARP that focus on public ceremonies of passage of girls do not address the underlying social values associated with FGM. However, a public declaration ceremony could be

integrated into other programmatic interventions that seek to empower women and girls to encourage abandonment.

Given the discriminatory nature of FGM, the focus on the agency and empowerment of girls and women in efforts to end FGM is critical. However, interventions that focus on girls' and women's agency and empowerment are few and their implementation process is poorly documented and evaluated (UNFPA–UNICEF, 2017). There is a need to strengthen the focus of such interventions as a core model in FGM programming.

While this review has highlighted two approaches at the individual level that focus on girls and women, it also highlights the gap in evidence on men's and boys' engagement in changing social and gender norms. Research has shown that to sustainably shift existing social and gender norms, men and boys need to be actively engaged in questioning and challenging power dynamics in their settings (Axelsson and Strid, 2020; Jewkes et al., 2015; Strid and Axelsson, 2020) engagement of all men and boys in action to prevent violence against women and girls is essential. We discuss why this engagement approach is theoretically important and how prevention interventions have developed from treating men simply as perpetrators of violence against women and girls or as allies of women in its prevention, to approaches that seek to transform the relations, social norms, and systems that sustain gender inequality and violence. We review evidence of intervention effectiveness in the reduction of violence or its risk factors, features commonly seen in more effective interventions, and how strong evidence-based interventions can be developed with more robust use of theory. Future interventions should emphasise work with both men and boys and women and girls to change social norms on gender relations, and need to appropriately accommodate the differences between men and women in the design of programmes. Designing and evaluating contextually appropriate dialogues around power relations, sexuality, and harmful practices such as FGM are key.

The studies in the current review did not cover economic empowerment. In certain settings, gender inequality and harmful traditional practices such as FGM are associated with low levels of economic development and poverty (Refaei et al., 2016). Tackling FGM is even more difficult in situations where women are economically dependent or risk losing traditional access to economic gain and its associated power if they challenge existing normative behaviour such as FGM. Addressing gender inequality therefore starts with tackling the root causes of all forms of violence against women and girls, including FGM. More research is needed to assess the effectiveness of economic empowerment initiatives such as gainful employment, engagement in income generating activities and integration with other development programmes.

Service level: Services for FGM prevention, protection and care

Interventions at the service level aimed to protect women and girls from harmful practices such as FGM, prevent the occurrence of FGM and provide care to women and girls who have undergone the practice. Studies under this category assessed the effectiveness of training health-care

providers, capacity-building of the health system, and the use of rescue centres in ending FGM.

There are mixed results on the effectiveness of training health-care providers in ending FGM. A critical look at the contrasting findings shows that training that is competency-based and supported with a functional health system is likely to be effective in assisting health-care providers to offer preventative and therapeutic services to women and girls seeking care. There were fewer studies that assessed the effectiveness of rescue centres in ending FGM. However, the limited evidence suggests that while this approach offers a short-term solution in housing girls/women running away from FGM, it is limited as a viable strategy for ending the practice. The fact that rescue centres remove girls/women from the community – in most circumstances without the consent of family members – foments hostility between community members and those running the rescue centres, which works against the principle of encouraging community members to willingly abandon the practice. Importantly, rescue centres that are solely implemented as safe houses are unlikely to be effective, as they remove the girl or woman from the community but do not address the root causes of FGM.

Although there were few studies of interventions at the service level, this review highlights the challenge associated with accessing services for girls and women either at risk of FGM or affected by the practice. It is important to note that while this review includes a few studies on quality of care, another body of literature on quality-of-care research exists (and would require an expanded search). Nonetheless, findings from the few studies considered by this review show that evidence on the effectiveness of building the capacity of the health-care system, including the integration of FGM into health services, is needed. This should include the provision of physical, sexual and psychosocial care, including referrals for psychosocial, legal or other social services, and in- and out-of-school support and referrals (UNFPA–UNICEF, 2017).

The review highlighted a gap in the range of services that focus on prevention, protection and care. Only medical services and rescue centres featured in the review, underscoring the need to consider other interventions at the service level that address gaps in FGM prevention, protection of women and girls, and provision of care to those affected by the practice. This includes interventions that build and strengthen capacities of social and legal service providers to address FGM; efforts to mainstream FGM in school curricula and social protection programmes targeting girls and women; and provision of legal, social and health services for prevention and response to FGM.

A holistic and multisectoral approach to FGM interventions

Most of the studies reviewed consistently advocated for the implementation of holistic and multisectoral interventions to end FGM. The multisectoral approach envisages a scenario where: laws and policies are in place and enacted, and budgets and coordinated systems are in place; community members, including men and boys and religious leaders, deliberate new norms and are equipped with the skills to motivate others to abandon

FGM; girls and women are empowered to defend their rights and access education, social, health and legal services; and FGM is mainstreamed in social development and services for women and girls. While the holistic and multisectoral approach is based on existing evidence on the pathways towards change, it is acknowledged that behaviour change processes are not linear; pathways of change are sometimes interdependent or cross-cutting (Cislaghi and Heise, 2018b; Davis et al., 2015; Heise, 1998). Synergies across the various levels (system, community, individual and service) are expected to enable FGM elimination and the advancement of gender equality. Additionally, it may not be practically possible for a programme to intervene fully in all the suggested domains. Instead, the scale of an FGM programme, existing partners and the local context should be considered in determining the most strategic combination of interventions (UNFPA–UNICEF, 2017, 2020).

Areas for further research

Adequately addressing FGM requires a holistic approach that brings together interventions that are sensitive to the complexity of FGM. This review has provided some insight into the effectiveness of interventions that have so far been implemented and assessed, but there remain several interventions for which there is insufficient evidence to determine their effectiveness in preventing and responding to FGM. Possible areas for further research are highlighted in Appendix 2.

Gaps and study limitations

This review is limited in its ability to reach a strong conclusion on the extent to which the assessed interventions led to a reduction in FGM prevalence, as most of the outcomes are related to knowledge and attitudinal changes. There is a need for study designs that assess the effectiveness of singular interventions and the complementarity of each intervention in complex interventions. Importantly, causality was not ascertained as part of this review, as most of the interventions assessed did not use rigorous study designs such as randomized controlled trials, which are the gold standard for determining effects of interventions on intended outcomes.

Given the complexity around shifting social norms and changing cultural practices such as FGM, it is expected that eradication of these practices will require the implementation of interventions for a longer period. Most of the interventions reviewed were implemented for a short time that may have been insufficient to observe changes in the practice. Equally importantly, this review only assessed evidence from existing evaluations, yet there may be many promising programmes being implemented that have not been evaluated.

The challenges of measuring social change range from the description of how change occurs during and after implementation of the various interventions, to the measurement of changes in FGM practice or attitudes. Measuring change requires standardized indicators that can be compared over time and across settings. It is noteworthy that a recent list of indicators developed by the UNFPA–UNICEF Joint Programme on FGM provides

an opportunity to measure the effectiveness of FGM interventions over time and across settings (UNFPA–UNICEF, 2020).

This review identified a general gap in monitoring, evaluation, and learning across the various interventions. Most of the monitoring and evaluation frameworks tended to focus more on process and intermediate outcomes, and less on demonstrating how interventions were effective in reducing FGM prevalence. The recently launched FGM measurement indicators by the UNFPA-UNICEF Joint Programme and the global framework for measuring changes in social norms related to FGM will help FGM programmes to develop robust and comprehensive monitoring and evaluation frameworks to facilitate adaptive programming (i.e., continuous improvements in decisions, policies and practices by testing what works and what does not) and learning. Equally relevant is an upcoming monitoring and evaluation framework for health systems addressing FGM (UNJP) and guidance on FGM surveillance models (WHO). These resources will assist implementing partners and donors in defining how to work adaptively, what adaptive approaches look like, and how adaptive approaches use evidence and data to make decisions.

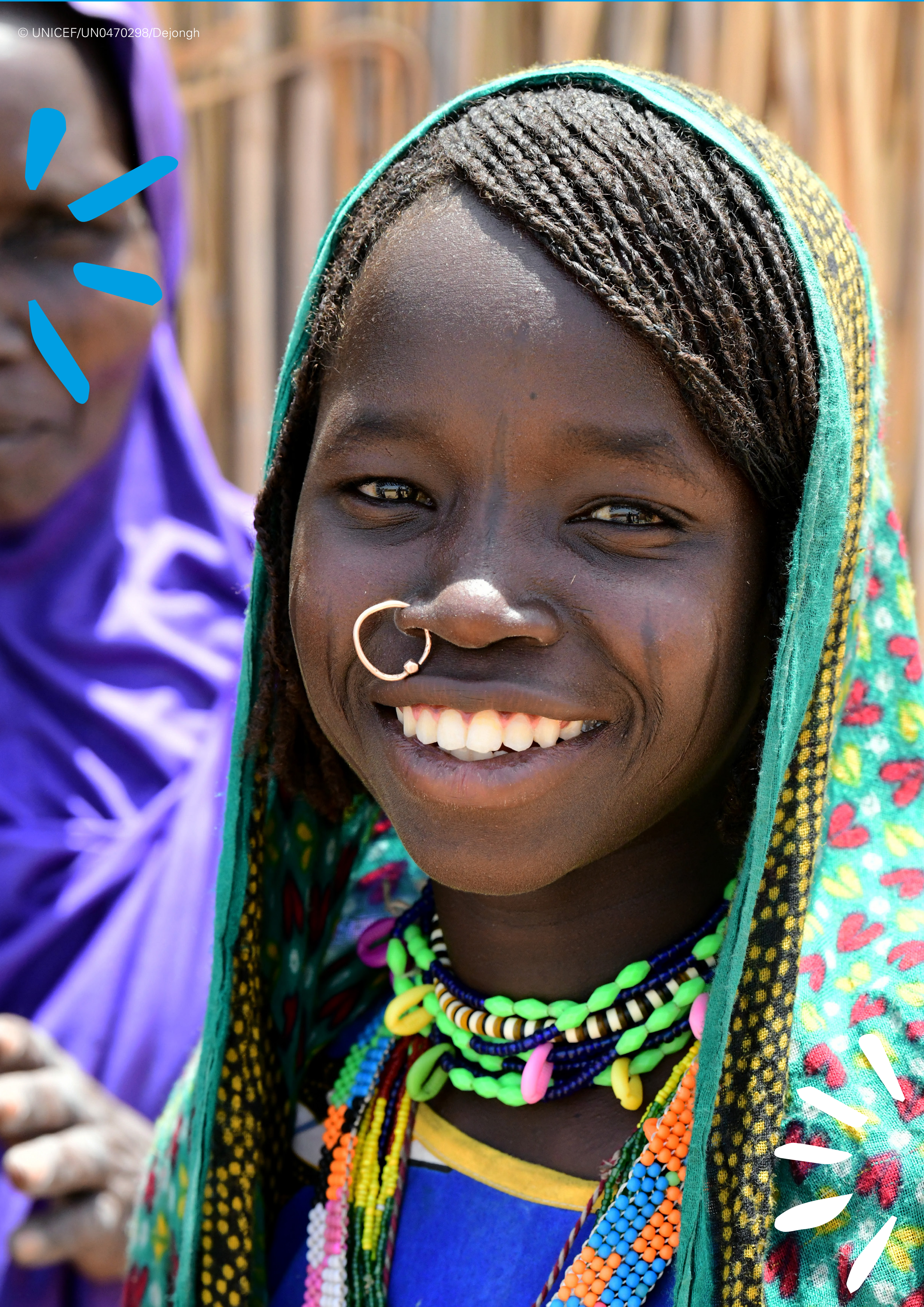
© UNICEF/UN0470298/Dejongh

CONCLUSIONS

Female genital mutilation is a practice with deep social and cultural underpinnings. This review has shown that at the system level, legislation may take a long time to change attitudes towards FGM and reduce the prevalence of the practice. However, a combination of legislation, enforcement and other interventions is likely to accelerate the reduction of FGM prevalence through changing the drivers of FGM. Community-level interventions are effective for changing attitudes towards FGM, but more must be done to innovate with these interventions so that they move beyond influencing attitudes to changing behaviour. At the individual level, formal education is effective in reducing FGM prevalence among girls. However, the returns from formal education in ending FGM may take many years to be realized. Interventions targeting intermediate outcomes, such as improvement in knowledge and change in attitudes and beliefs towards FGM, are equally important at the individual level. More research is needed on service-level interventions, especially on how the health system can effectively prevent and respond to FGM.

© UNICEF/UNI1394631/Dejongh

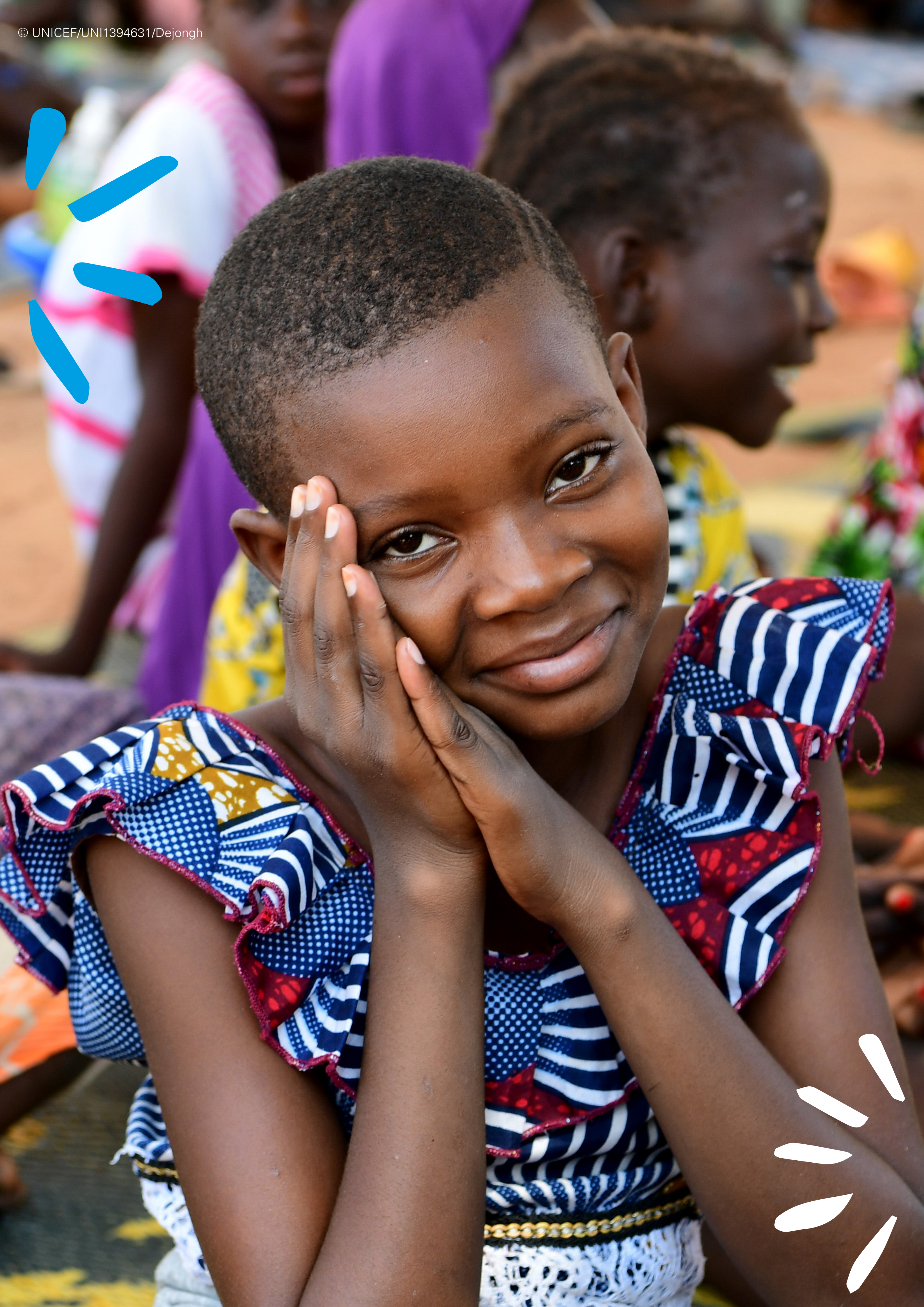

REFERENCES

Axelsson, Tobias K., and Sofia Strid, **Minority Migrant Men's Attitudes Towards Female Genital Mutilation: Developing Strategies to Engage Men**, *Health Care for Women International, vol. 41(6), 2020, pp. 709–726, <https://doi.org/10.1080/07399332.2019.1687707>, accessed 14 May 2021.*

Baillot, Helen, Nina Murray, Elaine Connelly and Natasha Howard, '**Addressing Female Genital Mutilation in Europe: A Scoping Review of Approaches to Participation, Prevention, Protection, and Provision of Services**', *International Journal for Equity in Health, vol. 17(1), article 21, 2018, <https://doi.org/10.1186/s12939-017-0713-9>, accessed 14 May 2021.*

Berg, Rigmor C., and Eva M. Denison, '**Effectiveness of Interventions Designed to Prevent Female Genital Mutilation/Cutting: A Systematic Review**', *Studies in Family Planning, vol. 43(2), 2012a, pp. 135–146, <https://doi.org/10.1111/j.1728-4465.2012.00311.x>, accessed 14 May 2021.*

Berg, Rigmor C., and Eva M. Denison, '**Interventions to Reduce the Prevalence of Female Genital Mutilation/Cutting in African Countries**', *Campbell Systematic Reviews, vol. 8(1), 2012b, pp. 1–155, <https://doi.org/10.4073/csr.2012.9>, accessed 14 May 2021.*

Berg, Rigmor C., and Eva M. Denison, '**A Realist Synthesis of Controlled Studies to Determine the Effectiveness of Interventions to Prevent Genital Cutting of Girls**', *Paediatrics and International Child Health, vol. 33(4), pp. 322–333, <https://doi.org/10.1179/2046905513Y.0000000086>, accessed 14 May 2021.*

Cislaghi, Beniamino, and Lori Heise, '**Using Social Norms Theory for Health Promotion in Low-Income Countries**', *Health Promotion International, vol. 34(5), 2018a, pp. 616–623, <https://doi.org/10.1093/heapro/day017>, accessed 14 May 2021.*

Cislaghi, Beniamino, and Lori Heise, '**Theory and Practice of Social Norms Interventions: Eight Common Pitfalls**', *Globalization and Health, vol. 14(1), 83, 2018b, <https://doi.org/10.1186/s12992-018-0398-x>, accessed 14 May 2021.*

Covidence, '**Covidence Helps the World Create Knowledge**', *2021, <www.covidence.org>, accessed 14 May 2021.*

Davis, Rachel, *et al.*, '**Theories of Behaviour and Behaviour Change Across the Social and Behavioural Sciences: A Scoping Review**', *Health Psychology Review, vol. 9(3), 2015, pp. 323–344, <https://doi.org/10.1080/17437199.2014.941722>, accessed 14 May 2021.*

Denison, Eva, Rigmor Berg, Simon Lewin and Atle Fretheim, '**Effectiveness of Interventions Designed to Reduce the Prevalence of Female Genital Mutilation/Cutting**', *Norwegian Knowledge Centre for the Health Services (NOKC), No. 25–2009, 2009.*

Department for International Development, '**How to Note: Assessing the Strength of Evidence**', *Department for International Development, 2014, <www.gov.uk/government/publications/how-to-note-assessing-the-strength-of-evidence>, accessed 14 May 2021.*

Esho, Tammary, Jamlick Karumbi and Carolyne Njue, '**Rapid Evidence Assessment: Quality of studies Assessing Interventions to Support FGM/C Abandonment**,' *Population Council, New York, 2017, <https://knowledgecommons.popcouncil.org/departments_sbsr-rh/611/>, accessed 14 May 2021.*

Gay, Jill, Melanie Groce-Galis and Karen Hardee, '**What Works for Women and Girls: Evidence for HIV/AIDS Interventions**' *Population Council, The Evidence Project and What Works Association, Inc., New York, 2016*

Gray, J. A., and Chambers, L. W., '**Evidence-based Health Care: How to Make Health Policy and Management Decisions**', *Canadian Medical Association Journal, vol. 157(11), 997, p. 1598.*

Gray, J. A. M., **Evidence-Based Health Care and Public Health: How to Make Decisions about Health Services and Public Health**, *3rd ed., Elsevier Health Sciences, Oxford, 2009.*

Harari, Michael B., Heather R. Parola, Christopher J. Hartwell, and Amy Riegelman, '**Literature Searches in Systematic Reviews and Meta-Analyses: A Review, Evalua-**

tion, and Recommendations', *Journal of Vocational Behaviour, vol. 118, April 2020, 103377, <https://doi.org/10.1016/j.jvb.2020.103377>, accessed 14 May 2021.*

Heise, Lori, '**Violence Against Women: An Integrated, Ecological Framework**', *Violence Against Women, vol. 4(3), 1998, pp. 262–290, <https://doi.org/10.1177/1077801298004003002>, accessed 14 May 2021.*

Huber, Douglas, et al., '**Postabortion Care: 20 Years of Strong Evidence on Emergency Treatment, Family Planning, and Other Programming Components**', *Global Health: Science and Practice, vol. 4(3), 2016, pp. 481–494, <https://doi.org/10.9745/GHSP-D-16-00052>, accessed 14 May 2021.*

Jewkes, Rachel, Michael Flood and James Lang, '**From Work with Men and Boys to Changes of Social Norms and Reduction of Inequities in Gender Relations: A Conceptual Shift in Prevention of Violence Against Women and Girls**', *The Lancet, vol. 385(9977), 2015, pp. 1580–1589, <www.sciencedirect.com/science/article/abs/pii/S0140673614616834>, accessed 14 May 2021.*

Johansen, R. Elise B., Nafissatou J. Diop, Glenn Laverack, and Els Leye, '**What Works and What Does Not: A Discussion of Popular Approaches for the Abandonment of Female Genital Mutilation**', *Obstetrics and Gynaecology International, 2013, 348248.*

Mackie, Gerry, and John LeJeune, '**Social Dynamics of Abandonment of Harmful Practices: A New Look at the Theory**', *Special Series on Social Norms and Harmful Practices, Innocenti Working Paper, 6, 2009–06.*

Meroka-Mutua, Agnes, Daniel Mwanga, and Charles O. Olungah, '**Assessing When and How Law is Effective in Reducing the Practice of FGM/C in Kenya**', *Evidence to End FGM/C: Research to Help Girls and Women Thrive, Population Council, New York, 2020. OHCHR, UNAIDS, UNDP, UNECA, UNESCO, UNFPA, UNHRC, UNICEF, UNIFEM, and WHO, Eliminating Female Genital Mutilation: An Inter-Agency Statement, 2008*

Population Reference Bureau, '**Ending Female Genital Mutilation/Cutting: Lessons from a Decade of Progress**', *Population Reference Bureau, Washington, DC, 2013 <www.prb.org/progress-ending-fgm/>, accessed 14 May 2021.*

Refaei, Mansoureh, Soodabeh Aghababaei, Abolghasem Pourreza, and Seyedeh Z. Masoumi, '**Socioeconomic and Reproductive Health Outcomes of Female Genital Mutilation**', *Archives of Iranian Medicine, vol. 19(11), 2016, pp. 805–811.*

Shell-Duncan, Bettina, Amadou Moreau, Katherine Wander, and Sarah Smith, '**The Role of Older Women in Contesting Norms Associated with Female Genital Mutilation/Cutting in Senegambia: A Factorial Focus Group Analysis**', *PLOS ONE, vol. 13(7), 2018, e0199217, <https://doi.org/10.1371/journal.pone.0199217>, accessed 14 May 2021.*

Shell-Duncan, Bettina, Reshma Naik, and Charlotte Feldman-Jacobs, '**A State-of-Art-Synthesis of Female Genital Mutilation/Cutting: What Do We Know Now?**' *Population Council, New York, 2016, <www.popcouncil.org/uploads/pdfs/SOTA_Synthesis_2016_FINAL.pdf>, accessed 14 May 2021..*

Shell-Duncan, Bettina, Katherine Wander, Ylva Hernlund, and Amadou Moreau, '**Dynamics of Change in the Practice of Female Genital Cutting in Senegambia: Testing Predictions of Social Convention Theory**', *Social Science and Medicine, vol. 73(8), 2011, pp. 1275–1283, <https://doi.org/10.1016/j.socscimed.2011.07.022>, accessed 14 May 2021.*

Strid, Sofia, and Tobias K. Axelsson, '**Involving Men: The Multiple Meanings of Female Genital Mutilation in a Minority Migrant Context**', *NORA – Nordic Journal of Feminist and Gender Research, vol. 28(4), 2020, pp. 287–301, <https://doi.org/10.1080/08038740.2020.1786164>, accessed 14 May 2021.*

United Nations, **Declaration on the Elimination of Violence Against Women**, *Proclaimed by General Assembly resolution 48/104 of 20 December 1993, United Nations Human Rights Office of the High Commissioner, 1993, <www.ohchr.org/en/professionalinterest/pages/violenceagainstwomen.aspx>, accessed 14 May 2021.*

United Nations, Beijing Declaration and Platform for Action, United Nations, 1995, <www.un-.org/en/events/pastevents/pdfs/Beijing_Declaration_and_Platform_for_Action.pdf>, accessed 14 May 2021.

United Nations, 'The Sustainable Development Goals (SDGs)', *United Nations, 2015, <https://sustainabledevelopment.un.org/sdg5>, accessed 14 May 2021.*

UNESCO, Early and unintended pregnancy and the education sector: Evidence review and recommendations, *UNESCO Paris, 2017, <http://bit.ly/2stzBxO>, accessed 14 May 2021.*

UNFPA, 'Female Genital Mutilation', *United Nations Population Fund, 2019, <www.unfpa.org/female-genital-mutilation>, accessed 14 May 2021.*

UNFPA, 'Impact of the COVID-19 Pandemic on Family Planning and Ending Gender-based Violence, Female Genital Mutilation and Child Marriage', *Interim Technical Note. UNFPA, 2020, <www.unfpa.org/sites/default/files/resource-pdf/COVID-19_impact_brief_for_UNFPA_24_April_2020_1.pdf>, accessed 14 May 2021.*

UNFPA–UNICEF Joint Programme, 'Proposal for Phase III of the UNFPA–UNICEF Joint Programme Elimination of Female Genital Mutilation: Accelerating Change', *UNFPA and UNICEF, 2017, <www.unicef.org/media/71786/file/JP-FGM-Phase-III-Proposal.pdf>, accessed 14 May 2021.*

UNFPA–UNICEF Joint Programme, 'Measuring Effectiveness of Female Genital Mutilation Elimination: A Compendium of Indicators', *UNFPA and UNICEF, 2020, <www.unfpa.org/sites/default/files/pub-pdf/026_UF_CompendiumOfIndicatorsFGM_21-online_F.pdf>, accessed 14 May 2021.*

UNICEF, 'Female Genital Mutilation (FGM) Statistics', *UNICEF Data, February 2020, <https://data.unicef.org/topic/child-protection/female-genital-mutilation/>, accessed 14 May 2021.*

Varker, Tracey, et al., 'Rapid Evidence Assessment: Increasing the Transparency of an Emerging Methodology', *Journal of Evaluation in Clinical Practice, vol. 21(6), 2015, pp. 1199–1204, <https://doi.org/10.1111/jep.12405>, accessed 14 May 2021.*

WHO, 'Female Genital Mutilation Programmes To Date: What Works and What Doesn't', *Policy Brief, World Health Organization, 2011, <www.who.int/reproductivehealth/publications/fgm/rhr_11_36/en/>, accessed 14 May 2021.*

Wouango, Josephine, Ostermann, Susan L., and Daniel Mwanga, 'When and How Does Law Effectively Reduce the Practice of Female Genital Mutilation/Cutting?', *Evidence to End FGM/C: Research to Help Girls and Women Thrive, Population Council, New York, 2020.*

Zotero, 'Zotero is a free, easy-to-use tool to help you collect, organize, cite, and share research', *Corporation for Digital Scholarship, 2021, <www.zotero.org>, accessed 14 May 2021.*

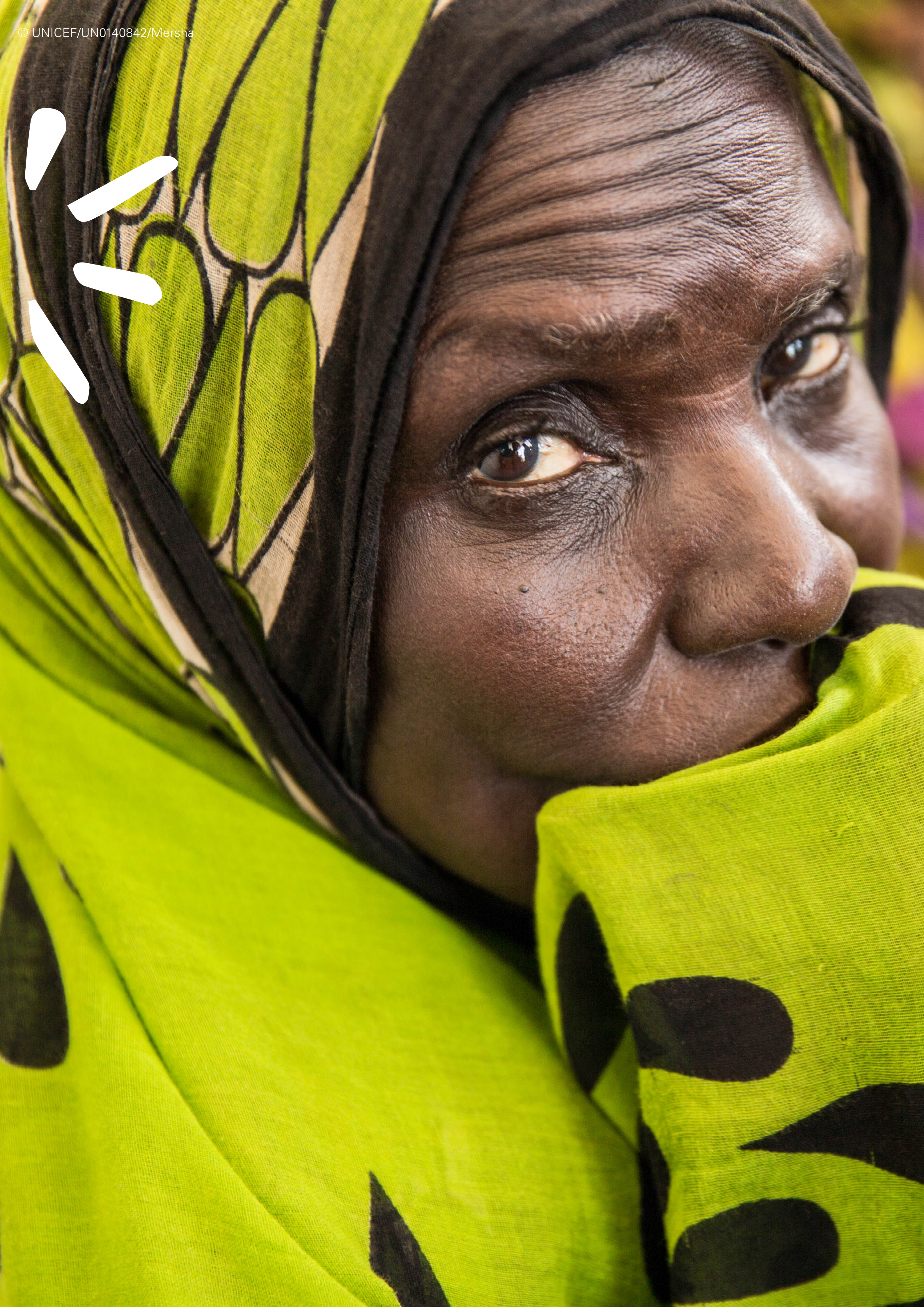
© UNICEF/UN0140842/Mersha

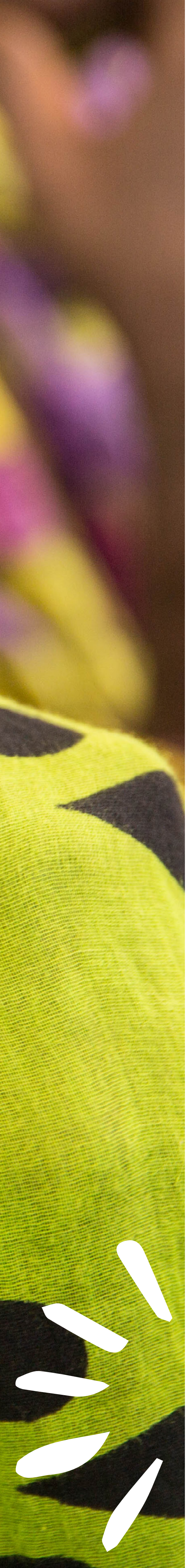

APPENDIX

Appendix 1. Summary of moderate- and high-quality studies

Appendix 2. Areas for further research

Type	Strength of evidence
I	Systematic review of multiple well-designed, randomized controlled trials.
II	Well-designed, randomized controlled trial of sufficient size.
IIIa	Well-designed trial/study without randomization that includes a control group (e.g., quasi-experimental, matched case-control studies, pre-post with control group).
IIIb	Well-designed trial/study without randomization that does not include a control group (e.g., single group pre-post, cohort, time series/interrupted time series, repeated cross-sectional studies).
IV	Well-designed, non-experimental study from more than one centre or research group, qualitative studies, and/or analysis of routine data.
V	Opinions of respected authorities, based on clinical evidence, descriptive studies, or reports of expert committees.

No.	Authors/year/title/publisher	Intervention classification and description	Study type and design	Key findings	Study quality	Strength of evidence/ Gray Scale
1	*Kipchumba, E, Korir, J, Abdirahman, N, Mwai, C. 2019. Accelerating Change towards Zero Tolerance to Female Genital Mutilation/ Cutting: Effects of Community Dialogues on FGM/C and Child Marriage. Norwegian Church Aid and Save the Children.*	Various interventions implemented. Mostly awareness creation through community conversations with different community groups, use of media, engaging religious and community leaders, engaging men and boys, capacity-building (training) of various groups and networks, advocacy on development and enactment of laws and policies.	Primary; Observational: Mixed methods (qualitative, quantitative, participatory). Panel survey was used to compare between the Midterm and baseline findings (pre-post). Focus group discussions (FGDs) and key informant interviews (KIIs) were used to collect in-depth information to explain and support the survey.	FGM prevalence remained almost universal. Increase in medicalization in Garbaharey and Belethawa districts in Gedo, Jubaland and Bosaso, Qardo, Garowe and Eyl districts in Puntland, Somalia. Community dialogues raised awareness on negative consequences of FGM which led to a change in FGM type practised and medicalization, but not support for total abandonment.	↑	IIIb
2	*Plugge, E, Adam, S, El Hindi, L, Gitau, J, Shodunke, N, Mohamed-Ahmed, O. 2019. The prevention of female genital mutilation in England: what can be done? Journal of Public Health, vol. 41(3), e261–e266.*	Legislation	Primary; Observational: Qualitative. FGDs and in-depth interviews (IDIs) to understand the communities' beliefs about how best to prevent FGM.	Legislation was counterproductive by alienating communities through its perceived imposition. Participants believed that the current UK legislation alone was not sufficient to tackle FGM.	↑	IV
3	*Van Raemdonck A. 2019. Paradoxes of awareness raising in development: gender and sexual morality in anti-FGC campaigning in Egypt. Culture, Health and Sexuality, vol. 21(10), pp. 1177–1191.*	Awareness creation campaign, health education through seminars/meetings	Primary; Observational: Qualitative (ethnographic).Researcher attended a series of awareness-raising seminars funded, organized and coordinated by the Coalition of NGOs against FGM. Also conducted IDIs with participants.	While the abandonment of FGM is encouraged, nationalist-modernist processes and dominant gender and sexual moralities are also reinforced. This means that certain aspects of development discourse are easily transmitted while others are subverted. Hegemonic discourses take centre stage while women's actual lives and experiences are de-centred.	→	V
4	*WHO. 2011. Female Genital Mutilation programmes to date: what works and what doesn't. Policy brief, World Health Organization.*	Various interventions reviewed: legislation, advocacy, awareness creation, capacity-building, alternative source of income for traditional practitioners, alternative rites of passage, communication, and youth involvement.	Secondary; Other review: literature review in which all anti-FGM programme documents were reviewed. Complemented with quantitative survey where a questionnaire was mailed to 365 national and international organizations.	Interventions which only supply information, education and campaigns (IEC) are not effective in changing behaviour. FGM legislation can be counterproductive. The community decision-making or consensus-building approach has significant potential for rural communities where collective decision-making is strongly valued. Using accurate media is an effective anti-FGM tool to bring about both awareness and behavioural change. Alternative income for traditional practitioners should not be the major strategy for change.	→	V
5	*Population Reference Bureau. 2013. Ending female genital mutilation/cutting lessons from a decade of progress. Population Reference Bureau, Washington DC.*	Various interventions reviewed: awareness campaigns, alternative incomes for traditional practitioners, alternative rites of passage, positive deviance, and safe houses.	Secondary; Other review: Desk review of evaluations studies. Systematic reviews, and donor and project reports. Supplemented with in-depth interviews with recognized experts in the field, including researchers and representatives of donor organizations.	Alternative incomes for traditional practitioners were not effective. Alternative rites of passage: success depended on local understanding and acceptance of the concept. Positive deviance: number of girls who did not undergo FGM increased during the programme. Safe houses: problematic as this approach may merely remove the girl or woman from the situation rather than address the social norms and pressures driving the practice; Legal measures: can have a negative impact in some communities, and must be complemented with comprehensive community-level interventions.	→	V
6	*Njue, C, Karumbi, J, Esho, T, Varol, N, Dawson, A. 2019. Preventing female genital mutilation in high income countries: a systematic review of the evidence. BMC Reproductive Health, vol. 16, article 113.*	Various interventions reviewed: education programmes to support affected women who may have undergone de-infibulation, legislation to protect girls, and community awareness to prevent FGM.	Secondary; Systematic review: searched databases and websites. Identified publications were screened against selection criteria, following the PRISMA guidelines.	Evidence of the effectiveness of interventions on reducing the prevalence of FGM is limited. All the evaluations measured short term outcomes, which showed improvements in knowledge but did not find any evidence on long term behaviour changes.	↑	IV

No.	Authors/year/title/publisher	Intervention classification and description	Study type and design	Key findings	Study quality	Strength of evidence/ Gray Scale
7	*Abdi, M, and Askew, I. 2009. A religious oriented approach to addressing female genital mutilation/cutting among the Somali community of Wajir, Kenya. Population Council, Washington DC.*	Awareness campaign /religious oriented approach: engaged religious scholars and educated the community about FGM with the aim of encouraging them to question why the practice is sustained and move towards abandonment.	Primary; Observational; Qualitative.	The approach led to some religious scholars and community members openly declaring their opposition to the practice, and many more privately opposing FGM.	→	V
8	*Ellsberg, M, Arango, DJ, Morton, M, Gennari, F, Kiplesund, S, Contreras, M, Watts, C. 2014. Prevention of violence against women and girls: what does the evidence say? Lancet, vol. 385(9977), pp. 1555–1566.*	Women empowerment: women-centred advocacy, group training for men and women, and economic empowerment.	Secondary; Systematic review: Reviewed evidence for interventions to reduce the prevalence and incidence of violence against women and girls.	The Tostan educational programme in Senegal led to a significant reduction in FGM among girls aged 0–10 years. Effective programmes such as Tostan are commonly participatory, engage multiple stakeholders, support critical discussion about gender relationships and the acceptability of violence, and support greater communication and shared decision making among family members.	→	IIIa
9	*Cetorelli, V, Wilson, B, Batyra, E, Coast, E. 2020. Female Genital Mutilation/Cutting in Mali and Mauritania: Understanding Trends and Evaluating Policies. Studies in Family Planning, vol. 51(1), pp. 51–69.*	Legislation: laws and policies	Primary; Quasi-experimental; Quantitative: A law banning FGM was introduced in Mauritania in 2005; in Mali, there is no legal ban on FGM. Used nationally representative survey data and a difference-in-difference method to evaluate the impact of the 2005 law.	The law did not have a significant impact on reducing FGM prevalence. Legislation on its own does not reduce prevalence of FGM.	↑	IIIa
10	*Cloward, K. 2014. False Commitments: Local Misrepresentation and the International Norms Against Female Genital Mutilation and Early Marriage. International Organization, vol. 68(3), pp. 495–526.*	Transnational activism /campaigns led by international actors.	Primary; Observational; Mixed methods: randomized field experiment and qualitative interviews.	Campaigns can lead to concealment of real behaviour and changes in attitudes. Transnational campaigns significantly influenced respondents in misrepresenting their behaviour and intentions related to FGM.	↑	IIIa
11	*Mehari, G, Molla, A, Mamo, A, Matanda, D. 2020. Exploring changes in female genital mutilation /cutting: Shifting norms and practices among communities in Fafan and West Arsi zones, Ethiopia. Population Council, Washington DC.*	Assessed the views of study respondents about the various interventions they had been exposed to: community conversation, legal intervention, religious and health-risks approaches.	Primary; Observational; Qualitative: In-depth interviews and focus group discussions.	In West Arsi: Changes in norms leading to FGM abandonment. In Fafan: no change in norms associated with FGM and no abandonment. Community conversation, legal intervention, religious and health-risks approaches can have varying impacts on different communities.	↑	IV
12	*Boyden, J, Pankhurst, A, Tafere, Y. 2012. Child protection and harmful traditional practices: female early marriage and genital modification in Ethiopia. Development in practice, vol. 22(4).*	Legislation - child protection laws	Primary; Observational; Longitudinal qualitative: three rounds of data gathered from a sub-sample of 50 boys and girls in 5 of the 20 Young Lives sites in Ethiopia, as well as with their peers, caregivers, and community representatives. Focus group discussions were held with boys and girls and adults. Interviews with a range of community and religious leaders, elders, and service providers.	The perceived efficacy of female early marriage and genital modification is manifested in continued resistance to reform, and unintended deleterious consequences in some cases. This suggests that there has been insufficient regard to the socio-cultural and economic context and to the rationale underlying these practices. The appropriateness and effectiveness of measures that focus on specific practices in isolation from wider social processes and relations is doubtful; there is also a need to consider reproductive health services, and measures to promote women's education and employment.	→	IV
13	*Shell-Duncan, B, Hernlund, Y, Wander, K, Moreau, A. 2013. Legislating Change? Responses to Criminalizing Female Genital Cutting in Senegal. Law and Society Review, vol. 47(4), pp. 803–835.*	Legislation	Primary; Observational; Mixed methods: Qualitative: 98 in-depth interviews and six focus group discussions were conducted. Quantitative: multistage sampling was used and villages stratified into Tostan and non-Tostan villages. Survey data were obtained from 265 women and 82 husbands.	While some people viewed the ban as a reason to reluctantly abandon FGM, others defied the ban and continued the practice underground. Among supporters of FGM, legal norms ran counter to social norms, and did little to deter the practice, and in some instances incited reactance or drove the practice underground. Where FGM was being contested, legislation served to strengthen the stance of those contemplating or favouring abandonment.	↑	IIIa

No.	Authors/year/title/publisher	Intervention classification and description	Study type and design	Key findings	Study quality	Strength of evidence/ Gray Scale
14	*Baillot, H, Murray, N, Connelly, E, Howard, N. 2018. Addressing female genital mutilation in Europe: a scoping review of approaches to participation, prevention, protection, and provision of services. International Journal for Equity in Health, vol. 17, article 21.*	Various interventions reviewed: awareness creation, legislation, and preventative child protection measures.	Secondary; Other review: Scoping review of literature supplemented with qualitative interviews (16 individual and 3 group interviews).	Several countries have developed promising interventions supporting FGM prevention and recovery. Gaps remain, including community participation, professional knowledge and linkages, and evaluation of approaches. Some countries, e.g., France have led with strong criminal justice responses, while others, e.g., the Netherlands have focused on preventative child protection measures.	↑	IV
15	*Vestbøstad, E, Blystad, A. 2014. Reflections on female circumcision discourse in Hargeysa, Somaliland: purified or mutilated? African Journal of Reproductive Health, vol. 18(2).*	Various interventions implemented: awareness campaigns, legislation, education – FGM topic included in revised national curriculum for nursing education.	Primary; Observational; Qualitative: Qualitative interviews and informal conversations with nursing/health science teachers and office workers at the hospital (10); nursing/health science students (40); women employed in the governmental and non-governmental offices working on FGM (3); women who had lived in exile but had returned to Hargeysa (5).	Limited abandonment but more of changing the type of cut and medicalization of the practice. Open discussions about the practice and change in attitudes. The change is expressed as one going from 'pharaoni' (infibulation) to sunna operations, but also from sunna to an abandonment of the practice.	→	IV
16	*Abathun, AD, Sundby, J, Gele, AA. 2018. Pupil's perspectives on female genital cutting abandonment in Harari and Somali regions of Ethiopia. BMC Women's Health, vol. 18, article 167.*	Awareness creation: school based awareness campaign	Primary; Observational; Quantitative: A school-based cross-sectional study conducted in the Somali and the Harari Regional States of eastern Ethiopia. 480 respondents participated in the study.	Change in attitude in terms of support for abandonment of FGM. Participants who received information through multiple information channels were more likely to support the abandonment of FGM than those who received information from a single source. Similarly, school-based awareness campaigns and TV-based media communications were the main sources of information that influenced a high proportion of young people to support the abandonment of the practice.	↑	IIIa
17	*Hassanin, IM, and Shaaban, OM. 2013. Impact of the complete ban on female genital cutting on the attitude of educated women from Upper Egypt toward the practice. International Journal of Gynecology and Obstetrics, vol. 120(3), pp. 275–278.*	Legislation and formal education	Primary; Observational; Quantitative: Cross-sectional survey was carried out at the outpatient clinics of two Upper Egypt hospitals.	Small but statistically significant reduction in FGM among daughters and reduction in medicalization. Little change in attitude among educated families in Upper Egypt. The law contributed to the reduction in FGM among daughters and medicalization of the practice.	→	IIIa
18	*Ako, MA, and Akweongo, P. 2009. The limited effectiveness of legislation against female genital mutilation and the role of community beliefs in Upper East Region, Ghana. Reproductive health matters, vol. 17(34), pp. 47–54.*	Legislation	Primary; Observational: Qualitative: In-depth interviews with six state officials, a circumciser, the president of a women's advocacy organization, and semi-structured interviews with 32 community members.	While having a law criminalising FGM is necessary, it is not sufficient for elimination purposes. Although FGM has been criminalised, political support to ensure that the law is effectively implemented has been lacking. The law led to very few arrests as families protected the circumcisers. It also drove the practice underground.	↑	IV
19	*Dowuona-Hammond, C, Atuguba, RA, Tuokuu, FXD. 2020. Women's survival in Ghana: What has law got to do with it? SAGE Open.*	Legislation	Secondary; Other review: Desk review of existing articles, books, government reports, policy documents and parliamentary proceedings, law reports, and other documentation on women empowerment.	The law has not been effective due to traditional belief systems related to FGM, lack of awareness of the law, and economic benefits to traditional practitioners. There has been a low level of enforcement. Laws alone are insufficient to change negative practices such as FGM and advance gender equality.	→	V
20	*Diop, NJ, Askew, I. 2009. The effectiveness of a community-based education program on abandoning female genital mutilation/cutting in Senegal. Studies in Family Planning, vol. 40(4), pp. 307–318.*	Education empowerment program: TOSTAN promotes an integrated approach to learning that offers a comprehensive curriculum and improving life skills and the socioeconomic conditions of participants with a strong human rights approach.	Primary; Quasi-experimental: Pre- and post-intervention longitudinal design with a comparison group.	Significant improvements in knowledge and attitudes toward FGM among women and men who had, and had not participated in the programme, without corresponding improvement in the comparison villages. The prevalence of FGM among daughters decreased significantly over time as reported by women who were directly and indirectly exposed to the programme, but not among daughters in the comparison villages.	↑	IIIa

No.	Authors/year/title/publisher	Intervention classification and description	Study type and design	Key findings	Study quality	Strength of evidence/ Gray Scale
21	*Rawat, R. 2017. The association between economic development, education and FGM in six selected African countries. African Journal of Midwifery and Women's Health, vol. 11(3).*	Education and economic development	Primary; Observational; Quantitative: Used demographic and health Survey and Multiple Indicator Cluster Survey datasets (2010–2013)	Higher levels of education for women were associated with lower levels of FGM. Education was statistically significant in changing the percentage of FGM practices in the selected countries. The economic status of women was directly associated with FGM, with FGM less likely to be found among more highly educated women.	↑	IV
22	*Kimani, S, Okondo, C. 2020. A diagnostic assessment of the health system's response to female genital mutilation/cutting management and prevention in Kenya. Population Council, Washington DC.*	Health system's response to FGM	Primary; Observational; Mixed methods: KIIs with FGM policy actors and service providers; FGDs with service providers; health facility assessments; observations of client–provider interactions, client exit interviews and service data abstraction.	The Kenyan health sector's capacity is inadequate in responding to FGM management and prevention. There were variations in awareness or knowledge of laws and policies that address FGM-related prevention and management; there was limited or inconsistent documentation of FGM-related cases and complications in the service delivery points; only minimal unstructured FGM prevention interventions were implemented; many women and girls were referred to higher-level facilities because providers lacked the capacity to address these complications; and, although women/girls were noted to present with FGM-related complications, there were no specific interventions prescribed in the guidelines and protocols to address them.	↑	IV
23	*Van Bavel, H. 2020. At the intersection of place, gender, and ethnicity: changes in female circumcision among Kenyan Maasai. Gender, Place & Culture, vol. 27(8), pp. 1071–1092.*	Various interventions: awareness creation, legislation, safe houses. The non-governmental organisation SAFE Maa developed an approach to challenge the social norm on FGM. The four key elements of the approach were: non-judgemental, community-led, intersectional, and showcasing wider change.	Primary; Observational; Qualitative: ethnographic approach that combines participant observation and semi-structured in-depth interviews (35).	There were changes in norms that drive FGM due to interventions implemented by the SAFE Maa project; approaches that empower people to have their own debates and make their own decisions about the future they want for their community have transformative potential.	↑	IV
24	*Galukande, M, Kamara, J, Ndabwire, V, Leistey, E, Valla, C, Luboga, S. 2015. Eradicating female genital mutilation and cutting in Tanzania: an observational study. BMC Public Health, vol. 15, article 1147.*	Education campaign (awareness creation): 5 school club visits; 8 community dialogue sessions; 10 training sessions on health risks of FGM involving children, local leaders and FGM practitioners; training on income generating activities; 5 training sessions involving the youth and Masai warriors; distribution of goats and chickens to ex- FGM practitioners; weekly school club activities, ex-FGM club meetings and quarterly local village advocacy subcommittee meetings; public declaration denouncing FGM and celebrating uncut girls.	Primary; Observational; Mixed methods: household survey (891 interviews); 8 KIIs (health worker, member of village health team, project coordinator, community leader, representatives from local women and men's groups); 11 FGDs with active and ex- FGM practitioners and community members; and 4 site observation visits. Baseline and endline assessments were conducted.	Multifaceted educational campaign achieved moderate success in increasing knowledge of the health risks and changing attitudes. However, its effectiveness in reducing FGM prevalence was uncertain – the practice is currently performed secretly.	↑	IIIb
25	*Kandala, NB, and Komba, PN. 2015. Geographic variation of female genital mutilation and legal enforcement in sub-Saharan Africa: a case study of Senegal. The American Journal of Tropical Medicine and Hygiene, vol. 92(4), 838–847.*	Legislation	Primary; Observational: Quantitative: used the 2010–2011 Senegal Demographic Health Survey and Multiple Indicators Cluster Survey (SDHS-MICS) covering 14,228 women and their daughters. For the enforceability of the anti-FGM law, desk research was used.	The analysis showed both advantages and vulnerabilities of the anti-FGM law in relation to the issue of enforcement. The law falls short of offering adequate protection to potential victims. Data showed a zero change in prevalence in the 5 years separating the surveys. However, there were large geographic variations within the country by region. The prevalence of FGM was still high, even after Senegal signed the Maputo Protocol in 2006.	↑	IIIb

No.	Authors/year/title/publisher	Intervention classification and description	Study type and design	Key findings	Study quality	Strength of evidence/ Gray Scale
26	*Berg, RC, and Denison, EM. 2013. A realist synthesis of controlled studies to determine the effectiveness of interventions to prevent genital cutting of girls. Paediatrics and International Child Health, vol. 33(4), pp. 322–333.*	Various interventions reviewed: training, formal classroom education, media communication, outreach and advocacy, and informal adult education. Specific: training of health personnel, education of female students, communication programme, outreach, and advocacy, Tostan education programme.	Secondary; Realist Synthesis Approach: identified 8 effectiveness studies and 27 context studies. The review incorporated randomized controlled trials, quasi-randomized trials, controlled before-and-after studies, and interrupted time series designs on the effectiveness of interventions. To identify context factors, the study included cross-sectional quantitative studies, qualitative studies, and mixed methods studies.	The driving force for changing FGM-related behaviour was thought to be the dissemination of information. Training of health personnel: no significant difference between the intervention and comparison groups regarding any outcome; education of female students: increased students' knowledge of the likely complications of FGM; communication programme: shift in perspective regarding FGM; Outreach and advocacy: in an Ethiopian context, it triggered an improvement in knowledge of harmful consequences of FGM, belief that it compromised the human rights of women, and intentions not to perform FGM in the future. Conversely, in a similar context involving Somali refugees, the intervention failed to generate significant change; Tostan education programme: programme resulted in negligible and small positive effects.	↑	IIIa
27	*Van Bavel, H, Coene, G, Leye, E. 2017. Changing practices and shifting meanings of female genital cutting among the Maasai of Arusha and Manyara regions of Tanzania. Culture, Health and Sexuality, vol. 19(12), pp. 1344–1359.*	Alternative rites of passage, formal education, and legislation.	Primary; Observational; Qualitative: IDIs with 21 women and 22 men; KIIs with 5 female and 1 male circumciser, 2 traditional Maasai leaders, 7 teachers, 2 priests, 7 heads of local women's organizations, and 3 nurses.	Changing attitudes towards FGM among the younger generation as the result of education; the law has led to the practice being performed in secret to avoid prosecution and negative judgements by opponents. Conducting FGM in secret has caused a disconnect between the practice and the initiation ceremony. Alternative rites of passage offer those willing to continue the practice the opportunity to do so without being prosecuted, and those unwilling to undergo or perform FGM the opportunity to evade it by faking the cutting without being socially sanctioned for it.	↑	V
28	*Evans, WD, et al. 2019. The Saleema initiative in Sudan to abandon female genital mutilation: Outcomes and dose response effects. PLOS ONE, vol. 14(3).*	Various interventions implemented: awareness campaign, social dialogue and providing role models showing that uncut girls are socially acceptable. Specific activities included publicly pledging to abandon FGM and support the Saleema initiative, wearing Saleema colours as a sign of support, public dialogue on the existence of FGM, its role in society, and the need for abandonment, and pledges not to cut newborn daughters immediately after birth.	Primary; Quasi-experimental design: controlled for dosage of campaign messages delivered across the 18 states in Sudan to measure a dose-response effect. Social norms were operationalized through a 4-item scale.	Saleema's social marketing strategy was effective in reducing pro-FGM social norms.<br><br>Higher levels of exposure to Saleema led to reduced pro-FGM social norms. Self-reported exposure was significantly associated with reduced pro-FGM social norms. Additionally, higher doses of Saleema, measured through an exogenous measure of campaign event exposure from an independent monitoring system, was associated with reduced pro-FGM social norms.	↑	IIIa
29	*Denison, E, Berg, RC, Lewin, S, Fretheim, A. 2009. Effectiveness of Interventions Designed to Reduce the Prevalence of Female Genital Mutilation/Cutting. Norwegian Knowledge Centre for the Health Services.*	Various interventions reviewed: education, advocacy, empowerment, and training health personnel.	Secondary; Systematic review: searched for relevant literature in scientific databases, in databases of international organizations engaged in projects concerning FGM, and in reference lists of relevant reviews and included studies. Analysis included six controlled before-and-after studies. Calculated effect estimates in outcomes for which pre- and post-scores for both intervention and comparison groups were reported.	There is a paucity of high-quality evidence regarding the effectiveness of interventions to prevent FGM and the evidence base is insufficient to draw solid conclusions.<br><br>The effect estimates suggest that (1) training health personnel likely produced no effects in knowledge or beliefs/attitudes about FGM; (2) educating female students may possibly have led to a small increase in knowledge /awareness about FGM; (3) multifaceted community activities may possibly have increased the proportion of participants having favourable cognitions and intentions about FGM; (4) community empowerment through education may possibly have positively affected prevalence of FGM, participants' knowledge about the consequences of FGM, and regrets about having had daughter cut.	↑	IIIa

No.	Authors/year/title/publisher	Intervention classification and description	Study type and design	Key findings	Study quality	Strength of evidence/ Gray Scale
30	*Abreu, W, and Abreu, M. 2014. Community education matters: representations of female genital mutilation in Guineans immigrant women. Procedia – Social and Behavioral Sciences, vol. 171, pp. 620–628.*	Education programme as a form of alternative ritual	Primary; Observational; Qualitative: semi-structured interviews with eight immigrant women from Guinea Bissau living in Portugal.	Educational programs emerged as an alternative to the cutting. The educational programs which involved the community and respected the local culture contributed to the reduction of FGM prevalence.	→	V
31	*Ethiopian Evangelical Church Mekane Yesus Development and Social Service Commission (EECMY-DASSC/WBS-BO). 2012. Final-term Evaluation Report of Sinana Female Genital Mutilation Elimination Project (SFGMEP 2010-2012). NORAD.*	Various interventions implemented: training FGM practitioners, trainers of trainers, male and female children and youths, women and men, religious leaders and project and government staff; establishing and training anti-FGM committees and clubs; organizing experience sharing visits and partners' consultation forum; and supplying IEC/BCC materials.	Primary; Observational; Qualitative: discussion with the project management and staff; reviewed the project document as well as periodical reports of the project; discussion with the project beneficiaries – community members, Kebele administrators, religious leaders, community organisation leaders, students, anti-FGM club members, teachers, circumcisers, and teachers.	FGM practitioners rejected the practice; girls started saying no to FGM; religious leaders took initiatives and checked whether there is or no obligatory attachment of FGM in Koran and Bible; girls married without FGM; both women and men openly discussing about impacts of FGM and other harmful traditional practices which was unthinkable and a taboo before.	→	V
32	*Masas, J. 2009. Evaluation Report for the Anti Female Genital Evaluation Report for the Anti Female Genital Mutilation Maasai (FGM) Project: An FPFK Advocacy programme for the Maasai of Southern Rift Region of Kenya. NORAD.*	Various interventions implemented: human rights, alternative rites of passage, capacity-building and institutional strengthening, communication interventions, and community change and development. Specific: mobilize local churches to advocate for the rights of Maasai girls and women with a view to stop the practice of FGM and subsequent early and forced marriages; campaigns at the village/community level; knowledge dissemination and capacity building; and advocacy and awareness raising.	Primary; Observational; Qualitative: KIIs with staff, board members, and the project's steering committee members; IDIs and group discussions with children; key stakeholders including chiefs, the politicians, community leaders and members, parents, cooperating agencies, church leaders and members, and schoolteachers.	Girls within the church stopped undergoing FGM; parents were knowledgeable about the disadvantages of FGM and discouraged their girls from undergoing FGM; men within the church were supportive of anti-FGM campaigns; entire church community supported the anti-FGM advocacy campaigns. Community members who still practice FGM do it in secret; and some children are taken across the border to Tanzania where they undergo FGM.	→	V
33	*Nambisia, EM. 2014. Measures Influencing Eradication of Female Genital Mutilation Practices Among the Maasai Community in Maparasha Constituency Kajiado County, Kenya. University of Nairobi (UoN) Digital Repository.*	Various interventions assessed: legislation, ARP, advocacy, capacity building through trainings, rescue centres, international regulatory instruments, and girl child education.	Primary; Observational; Mixed methods: a descriptive survey with a population of 666, comprising male village elders (60), women village elders (40), girls from the community who were employed (396) and community members (170). Selection of the sample from each category was done using stratified random sampling. Qualitative interviews were also conducted	Girl-child education played the most important part in FGM eradication among the Maasai. The current constitution protects children from FGM. ARP contributed to the reduction of FGM. Increased awareness on the need to eradicate FGM. The legal framework awareness is in place but implementation is still a challenge. Respondents disagreed that reconciliatory meetings between girls who escaped to rescue centres and parents was always successful and thus questioned the approach's impact in the eradication of FGM.	→	IIIb
34	*Brown, E, Porter, C. 2016. The Tackling FGM Initiative Evaluation of the Second Phase (2013–2016). Options Consultancy Services Limited.*	Various interventions implemented: human rights, legal, health services provision and psychosocial support, health care professionals training, capacity-building and institutional strengthening, communication interventions, and community change and development.	Primary; Observational; Mixed methods: review of the projects' M&E data, survey data on attitudes (people from affected communities and professionals), and KIIs.	Strong evidence from both the survey and qualitative data of an attitudinal shift towards rejecting FGM in the areas where the projects worked, as indicated by the number of respondents who did not wish FGM to continue. In a minority of cases, there was still support for FGM, particularly of forms perceived to be 'milder'. Child protection arguments have gained traction and enabled more widespread support for ending FGM.	→	IV

No.	Authors/year/title/publisher	Intervention classification and description	Study type and design	Key findings	Study quality	Strength of evidence/ Gray Scale
35	*Ugwu, I,and Ashaver, AN. 2014. TFD and community education on female genital mutilation in Igede land of Benue state: Ugengen community experience. Creative Artist: A Journal of Theatre and Media Studies, vol. 8(2).*	Media/social marketing/ communication: the use of theatre (TFD – Theatre for Development) as a medium of communicating the consequences of FGM. The community was involved in the processes of problem analysis, drama formation, rehearsals, performance, and discussion.	Primary; Observational; Qualitative; Cross-sectional design.	People became aware of and ready to take actions on FGM. Theatre may have helped to reshape community worldview about FGM.	→	V
36	*Bedri, P. 2020. Improved Understanding of FGM/C Abandonment among Sudanese Families in Khartoum and Kassala States. Sudan Working Paper, CMI, Norway.*	Positive deviance – to examine the characteristics of abandoning families, their motivations, and challenges.	Primary; Observational; Qualitative: cross-sectional design. In-depth interviews were conducted.	Strong role of men in the decision to abandon FGM. FGM abandonment is a complex process, and potential for relapse still exists within families that have abandoned.	↑	V
37	*McCracken, K. 2017. The Mayor's Office for Policing and Crime Female Genital Mutilation Early Intervention Model: An Evaluation. Opcit Research.*	Support services and community engagement: intervention targeted 3 areas of London (Tri-Borough, Tower Hamlets, and Waltham Forest), and involved prevention care and support for women that have undergone FGM as well as legal aspects.	Primary; Observational; Qualitative: cross-sectional design.	Change in attitude towards FGM among affected women; increase in specialized care and support for affected women; increased open discussion on FGM; overall improved services and service delivery.	→	IIIb
38	*Cislaghi, B, et al., 2019. Changing social norms: the importance of "organized diffusion" for scaling up community health promotion and women empowerment interventions. Prevention Science, vol. 20(6), pp. 936–946.*	Community dialogue: evidence of empowerment and norms change from three case studies – Community Empowerment Program in Mali, Change Starts at Home in Nepal, and Voices for Change in Nigeria.	Secondary; Other reviews	Community dialogue was effective in changing social norms through 'organized diffusion'.	→	IIIb
39	*Mahgoub E, et al., 2019. Effects of school-based health education on attitudes of female students towards female genital mutilation in Sudan. Eastern Mediterranean Health Journal, vol. 25(6), pp. 406–412.*	Health education: the study included three phases; in the pre-intervention phase, data were collected from 154 students using a pre-tested questionnaire, after which students received health education sessions. The same questionnaire was used to re-collect the data in a post-intervention phase 6 weeks later.	Primary; Observational; Quantitative: Pre-post design	School-based health education has a positive impact on both knowledge and attitude of female students towards FGM in Sudan. The means of knowledge and attitude scores significantly increased comparing pre-intervention scores to post-intervention scores	↑	IIIa
40	*Muthumbi J, et al., 2015. Female genital mutilation: a literature review of the current status of legislation and policies in 27 African countries and Yemen. African Journal of Reproductive Health, vol. 19(3), pp. 32–40.*	Legislation: assessing the extent to which legislation, national plans of action and the integration of FGM in health frameworks have been effective in fostering the prevention of FGM and/or in accelerating its abandonment.	Secondary; Literature review that assessed the impact of Female Genital Mutilation (FGM) legislation in 28 countries (27 in Africa and Yemen) where FGM is concentrated.	Legal frameworks have had a limited impact on societal attitudes and perceptions of FGM, with evidence suggesting rigid enforcement of FGM laws has in some instances been counterproductive. Legal approaches should be complemented by measures that address the underlying socio-cultural causes that are the root of FGM.	→	V

No.	Authors/year/title/publisher	Intervention classification and description	Study type and design	Key findings	Study quality	Strength of evidence/ Gray Scale
41	*Berg, RC, and Denison, E. 2012. Interventions to reduce the prevalence of female genital mutilation/cutting in African countries. Campbell Systematic Reviews.*	Various interventions reviewed: these included reviews of studies that have assessed intervention based on human rights frameworks, legal mechanisms, health risks, alternative rites, positive deviance, training health workers as change agents, training and converting circumcisers, and the use of comprehensive social development processes.	Secondary; Systematic review. Analysis included 8 effectiveness studies and 27 context studies.	Interventions were based on a theory that dissemination of information improves cognitions about FGM, but the interventions' success was contingent upon a range of contextual factors. With only eight controlled intervention evaluations meeting the inclusion criteria, all characterised by low methodological quality and no more than two studies synthesised in a meta-analysis for any given outcome, few firm conclusions can be drawn. Nonetheless, results point to possible advantageous developments because of FGM abandonment interventions, including lower prevalence of FGM, and changes in cognitions about FGM, such as beliefs about the practice.	↑	IIIa
42	*Winterbottom, A, Koomen, J, Burford, G. 2009. Female genital cutting: cultural rights and rites of defiance in northern Tanzania. African Studies Review, vol. 52(1), pp. 47–71.*	Multiple interventions assessed: health approach, ARP, and education.	Secondary; Other reviews	Campaigns against FGM using education, health, legal, and human rights–based approaches are at times ineffective and counterproductive when they frame the practice as a 'tradition' rooted in a 'primitive' and unchanging culture.	→	V
43	*Berg, RC, and Denison, E. 2012. Effectiveness of interventions designed to prevent female genital mutilation/cutting: a systematic review. Studies in Family Planning, vol. 43(2), pp. 135–146.*	Various interventions assessed: human rights frameworks, legal mechanisms, health risks, alternative rites, positive deviance, training health workers as change agents, training and converting circumcisers, and the use of comprehensive social development processes.	Secondary; Systematic review. Analysis included 8 effectiveness studies and 27 context studies.	Findings indicate that 19 of 49 outcomes were significantly different at study level, mostly favouring the intervention, but results from 4 meta-analyses showed considerable heterogeneity. The limited effectiveness and weak overall quality of the evidence from the studies appear related to methodological limitations of the studies and shortcomings in the implementation of the interventions.	↑	IIIa
44	*Graamans, EP, et al., 2019. Lessons learned from implementing alternative rites in the fight against female genital mutilation/cutting. The Pan African Medical Journal, vol. 32, p. 59.*	Alternative rites of passage (ARP): Amref Health Africa's efforts to end FGM/C through ARP	Primary; Observational; Qualitative; Cross-sectional design	ARP's success hindered by lack of clarity in implementation/intervention strategy at programme level, and strongly held community perceptions including risk of exclusion; loss of cultural identity; negative stereotyping; and outsider interference.	→	V
45	*Rasheed, SM, Abd-Ellah, AH, Yousef, FM. 2011. Female genital mutilation in Upper Egypt in the new millennium. International Journal of Gynecology and Obstetrics, vol. 114(1), pp. 47–50.*	Legislation: questionnaire administered to all girls and women visiting two hospitals in Egypt, their parents and providers to establish experience, knowledge and perceptions of FGM.	Primary; Observational Quantitative; Cross-sectional design	The incidence of FGM is still very high in Upper Egypt despite the criminalization law. While general practitioners perform most procedures, most nurses are in favour of preserving the practice. In the vast majority of cases, the procedures were performed by general practitioners.	→	IV
46	*Abdulah, DM, Dawson, A, Sedo, BA. 2020. The impact of health education on attitudes of parents and religious leaders towards female genital mutilation. BMJ Sexual and Reproductive Health, vol. 46, pp. 46–58.*	Health education: short-term educational intervention to change the attitudes of parents and religious leaders towards FGM/	Primary; Observational; Quantitative; Pre-post design	The attitudes of Mullahs, Mokhtars and parents substantially changed from a position of supporting female circumcision to expressing a wish to abandon the practice and not cut their future daughters. Study suggests that brief educational interventions can be an effective strategy for changing the attitudes of parents and public leaders towards FGM.	↑	IIIb
47	*Small, E, Sharma, BB, Nikolova, SP, Tonui, BC. 2020. Hegemonic masculinity attitudes toward female genital mutilation/cutting among a sample of college students in northern and southern Sierra Leone. Journal of Transcultural Nursing, vol. 31(5), pp. 468–478.*	Formal education: the influence of education on hegemonic masculinity attitudes between male and female students and whether parental education differentiated the groups.	Primary; Observational; Quantitative; Cross-sectional design	Formal education did not change the attitudes of students toward FGM. However, parental education had an influence on both male and female students' attitudes toward FGM.	↑	IIIb

No.	Authors/year/title/publisher	Intervention classification and description	Study type and design	Key findings	Study quality	Strength of evidence/ Gray Scale
48	*Matanda, D, Okondo, C, Kabiru, CW, Shell-Duncan, B. 2018. Tracing change in female genital mutilation/cutting: Shifting norms and practices among communities in Narok and Kisii counties, Kenya. Population Council, Washington DC.*	Various interventions assessed: alternative rites of passage, legislation, community education.	Primary; Observational; Qualitative; Cross-sectional design	While there have been positive changes in norms and attitudes related to FGM and abandonment increasingly being adopted, the negative changes have been to make FGM secretive due to fear of prosecution, to perform FGM on younger girls in order to reduce the chance of refusal and faster healing and to medicalize the practice as a result of health education.	↑	IV
49	*Ruiz, IJ, Martínez, PA, Giménez, LG. 2017. Eradicating Female Genital Mutilation; a viable reality. Raising awareness in the men involved. Procedia-Social and Behavioral Sciences.*	Public declarations: eradication of FGM via the testimony of men from countries where FGM is performed.	Primary; Observational; Qualitative; Cross-sectional design	Sensitized men who have participated in awareness-raising and health education programmes can change their viewpoint regarding FGM and make declarations. There was gradual rise in the awareness of the male population regarding the problems inherent in FGM and the sexist connotations harboured therein.	→	IV
50	*Afifi, M. 2009. Women's empowerment and the intention to continue the practice of female genital cutting in Egypt. Archives of Iranian Medicine, vol.12(2), pp. 154–160.*	Women empowerment: used national representative sample of 14,393 currently married women in Egypt to measure the level of empowerment and intention to continue practising FGM.	Primary; Observational; Quantitative; Cross-section design using demographic and health survey (DHS)	Women with high levels of empowerment and education were more likely not intending to perpetuate FGM for their daughters than low empowered low-educated women.	↑	IIIb
51	*Mwendwa, P, et al., 2020. "Promote locally led initiatives to fight female genital mutilation/cutting (FGM/C)" lessons from anti-FGM/C advocates in rural Kenya. Reproductive Health, vol. 17(1).*	Various interventions assessed: explored the views of anti-FGM advocates on the barriers and facilitators to tackling FGM within the Meru community.	Primary; Observational; Qualitative; Cross-sectional design.	The initiatives to tackle FGM have demonstrated considerable success and as a result, the shame and disrepute that once was associated with those who had not undergone the procedure is now very much focused on those who still practice, support or enable FGM (change in attitude towards FGM).	↑	IV
52	*Waigwa, S, Doos, L, Bradbury-Jones, C, Taylor, J. 2018. Effectiveness of health education as an intervention designed to prevent female genital mutilation/cutting (FGM/C): a systematic review. Reproductive Health, vol. 15(1), p. 62.*	Health education: a systematic review of health education interventions to prevent FGM in Nigeria, Ghana, Kenya, Morocco, Egypt, Senegal and Ethiopia.	Secondary; Systematic review; 12 studies included in the analysis	Effectiveness of FGM health education interventions depended on factors linked to sociodemographic and socioeconomic factors; traditions and beliefs; and intervention strategy, structure and delivery.	↑	IIIa
53	*Ameyaw, EK, et al., 2020. Female genital mutilation/cutting in Sierra Leone: are educated women intending to circumcise their daughters? BMC International Health and Human Rights, vol. 20, article 19.*	Formal education: assessing the effect of education among women, and intention to circumcise daughters	Primary; Observational; Quantitative; Cross-sectional design using demographic and health survey (DHS) data	Women who had no formal education were more likely to intend to circumcise their daughters than those with formal education.	→	IIIb
54	*Barrett, HR, et al. 2020. Transforming social norms to end FGM in the EU: an evaluation of the REPLACE Approach. Reproductive Health, vol. 17(40).*	Community engagement: evaluated the REPLACE Approach (mainly behaviour change and community engagement intervention) through extensive engagement with eight FGM affected African diaspora communities in five EU countries.	Primary; Observational; Mixed methods; Cross-sectional design	The use of interventions that are contextually appropriate, locally generated and where the community is fully engaged are likely to be successful in changing norms and attitudes. The evaluation demonstrated that the REPLACE Approach has the potential, over time, to bring about changes in norms and attitudes associated with FGM.	→	IV
55	*Johnsdotter, S. 2019. Meaning well while doing harm: compulsory genital examinations in Swedish African girls. Sexual and Reproductive Health Matters, vol. 27(2), pp. 87–99.*	Legislation	Primary; Observational; Quantitative: a review of police records	There appears to be largely negative effects of mandatory genital examination of Swedish African girls, with psychological trauma to the girls and parents being the main (unintended) outcome.	→	V

No.	Authors/year/title/publisher	Intervention classification and description	Study type and design	Key findings	Study quality	Strength of evidence/ Gray Scale
56	*Lien, IL, and Schultz, JH. 2013. Internalizing knowledge and changing attitudes to female genital cutting/ mutilation. Obstetrics and Gynecology International.*	Health education and communication: information from different educational institutions, from seminars and conferences, from work as interpreters in hospitals, and from discussions among families and friends.	Primary; Observational; Qualitative; cross-sectional design	Most of the study participants changed their viewpoints on FGM after attending seminars, conferences, or training courses. Social and attitudinal change can happen slowly or in an instant. Not only verbal information works in an abandonment campaign, but songs, slides, and films addressing a wide variety of topics related to the procedure are also efficient tools. that can reach the innermost level of internalization, where the motivation to change behaviour will be the strongest	→	V
57	*McCracken K; et al., 2017. National FGM Centre: an evaluation. Department for Education, UK.*	Service provision to FGM survivors: capacity-building of health professionals: continuum of intervention that combined work with children's services, other statutory agencies and organizations with community outreach.	Primary; Observational; Qualitative; cross-sectional design	Improved capacity and knowledge of professionals led to increased FGM case management, improved referral pathways, as well as enhanced engagement with potentially affected communities through small scale outreaches.	↑	IIIb
58	*UNICEF Innocenti Insight. 2010. The dynamics of social change towards: The abandonment of female genital mutilation/cutting in five African countries. The UNICEF Innocenti Research Centre.*	Various interventions assessed: legislation; national policies; human rights; community conversations/engagement; and alternative rites of passage.	Primary and Secondary: Combined extensive literature reviews with qualitative and quantitative research of cross-sectional design	High effectiveness of holistic approaches in Senegal; limited effectiveness of ARP in Kenya; some success using community conversation and community dialogue in Ethiopia and Sudan.	→	IV
59	*Smith, MB, and Smith, K. 2012. Final Evaluation Report. Bristol FGM Community Development Project. FORWARD.*	Service provision: evaluation of support services to girls living in Bristol that have undergone FGM in order to enhance their agency and self-efficacy.	Primary; Observational; Qualitative; cross-sectional design	Changes in the confidence, knowledge, awareness and self-determination of the women who were trained to be Community Health Advocates.	→	IV
60	*Ogalleh, SA. 2014. Final Evaluation of Community Education on Female Genital Mutilation (FGM) in Somaliland. International Solidarity Foundation.*	Community engagement/ outreach: evaluation of Candlelight's two phased project on community education on FGM in Somaliland.	Primary; Observational; Qualitative; cross-sectional design	The programme contributed immensely to the change of mindset and behavioural changes, i.e.. change from practising pharaonic to sunna type. The different messages by various stakeholders was confusing and challenging to sell the anti-FGM message for total abandonment.	→	V
61	*Crisman, B, Dykstra, S, Kenny, C, O'Donnell M. 2016. The impact of legislation on the hazard of female genital mutilation/ cutting: regression discontinuity evidence from Burkina Faso. Center for Global Development Working Paper.*	Legislation: the impact of legislation on risk of cutting in Burkina Faso.	Primary; Observational; Quantitative; Cross-sectional design using demographic and health survey (DHS) data	While the law averted FGM for approximately 237,591 girls, the results varied across regions. Legislation was accompanied by other interventions and therefore the law's effect should be viewed with this context in mind.	↑	IIIb
62	*Buttia, C. 2015. Investigation of successful interventions in mitigation of female genital mutilation /cutting (FGM/C) among selected Kenyan communities: Maasai, Kisii and Kuria (Master thesis, Hochschule für angewandte Wissenschaften Hamburg).*	Various interventions reviewed: religious influence, women and girls empowerment, community education, girl education, health risk approach, exposure to other cultures, alternative rite of passage involving a reconciliation process, rescue camps, successful use of legislation, broad-based approach involving the whole community (ex-circumcisers, religious leaders, local animators and law officers) and community sensitization campaigns.	Secondary: other reviews	Pastors, priests and prophets of the three communities have managed to educate people/followers and convince parents that circumcising girls is not a requirement of the religion; education (formal and health education) has been used in the three communities to successfully reduce FGM; Alternative rites of passage has been successful though context matters; rescue centres face challenges such as limited resources and lack of its recognition especially among the Kuria; health risk education as stand-alone activity has faced the challenge of medicalization; Introducing of laws against strongly held practices such as FGM can lead to FGM being done secretly; broad-based approach involving the whole community has contributed to decrease in FGM cases.	→	V

No.	Authors/year/title/publisher	Intervention classification and description	Study type and design	Key findings	Study quality	Strength of evidence/ Gray Scale
63	*Mepukori, DN. 2016. Is alternative rite of passage the key to abandonment of female genital cutting? A case study of the Samburu of Kenya. Duke University.*	Alternative rite of passage (ARP): Amref programme that encourages communities to maintain the cultural ceremonies and rites surrounding female initiation whilst getting rid of FGM.	Primary; Observational; Qualitative; cross-sectional design	Sensitized community members were able to state the consequences of and oppose FGM. However, ARP training in Samburu is not universal, and the majority of the community remains largely unaware of the dangers of FGM and are unchanged in their attitudes.	↑	V
64	*Kaunga, S. 2014. Media strategies and their influence in communicating information on Female Genital Mutilation: a case of Meru community in Tharaka District. Doctoral dissertation, University of Narobi.*	Media/social marketing/ communication: the influence of media strategies in communicating information about female genital mutilation effectively among the Meru in Kenya.	Primary; Observational; Mixed methods Quantitative; cross-sectional design	Media ambassadors and the use of local language were positive and statistically significant in influencing effective communication on FGM. Conversely, the use of sheng (local slang) language and public participation were not statistically significant in influencing effective communication.	→	IIIb
65	*Varol, N, et al. 2015. The role of men in abandonment of female genital mutilation: a systematic review. BMC Public Health, vol. 15, article 1034.*	Role of men: explored men's attitudes, beliefs, and behaviours regarding FGM, as well as their ideas about FGM prevention and abandonment.	Secondary; Systematic review	The level of education of men was the most important indicator for men's support for abandonment of FGM. Social obligation and the lack of dialogue between men and women were acknowledged as barriers to abandonment.	↑	IIIa
66	*Family Support Institute. 2008. Final evaluation of Tostan Community Empowerment Programme (CEP) Report. Family Support Institute.*	Community engagement	Primary; Observational; Mixed methods; pre-post with a control design	Following implementation of the training programme, participants, especially women in the intervention villages, engaged in dialogue, learning, exchange of ideas, problem solving and decision making. The Tostan CEP also established functioning Community Management Committees that were transformative structures that ensured achievement of sustainable development as Tostan strengthened their capacities. This led to increased knowledge, changes in attitudes towards FGM and significant reduction of FGM prevalence in intervention villages.	→	IIIa
67	*Hassanin, IMA et al., 2008. Prevalence of female genital cutting in Upper Egypt: 6 years after enforcement of prohibition law. Reproductive BioMedicine Online, vol. 16, pp. 27–31.*	Legislation: the effect of the prohibition law of FGM in Egypt following a public outcry after a young girl died undergoing FGM in 2006.	Primary; Observational; Quantitative; Cross-sectional design	The practice of FGM in Upper Egypt remained high despite enforcement of the law. Prevalence of FGM was significantly higher in female students from rural areas as compared with urban areas. No significant differences were found in the pattern of distribution of FGM over the years from 2001 to 2006.	→	IIIb
68	*Suzuki, C, and Meekers, D. 2008. Determinants of support for female genital cutting among ever-married women in Egypt. Global Public Health, vol. 3(4), pp. 383–398.*	Anti-FGM communication messages: examined the effect of exposure to communication messages on support for FGM in Egypt.	Primary; Observational; Quantitative; cross-sectional design using demographic and health survey (DHS) data	There was evidence of effect of more media messages on support for discontinuing FGM among ever married women in Egypt. Women exposed to two or more FGM media messages were more likely than unexposed women to support discontinuation of FGM.	→	IIIb
69	*Camilotti, G. 2016. Interventions to Stop Female Genital Cutting and the Evolution of the Custom: Evidence on Age at Cutting in Senegal. Journal of African Economies, vol. 25(1), pp. 133–158.*	Legislation and awareness campaigns in Senegal: the law No. 99–05 of 29th January 1999 condemns whoever is responsible for a mutilation of the female genital organs to be imprisoned for between 6 months to 5 years.	Primary; Observational; Quantitative; Cross-sectional design using demographic and health survey (DHS) data	The law has limited effect in reducing prevalence of FGM. Instead, age at cutting has reduced as girls are cut much earlier. Girls born in a year and a region where the law against FGM has been legally enforced are cut almost one year earlier. Almost 90% of the girls born before 2009 were cut before age 5. Girls born in the year and in a region where the law was enforced are cut 0.74 years earlier than girls born in another year in the same region. No statistically significant effect of the law was found on the prevalence of FGM.	↑	IIIb

No.	Authors/year/title/publisher	Intervention classification and description	Study type and design	Key findings	Study quality	Strength of evidence/ Gray Scale
70	*Asekun-Olarinmoye, EO, and Amusan, OA. 2008. The impact of health education on attitudes towards female genital mutilation (FGM) in a rural Nigerian community. The European Journal of Contraception and Reproductive Health Care, vol. 13(3), pp. 289–297.*	Health education (health talks): information was given in vernacular language on the female genital anatomy, the nature and the types of FGM, the complications associated with the practice, and the beliefs that encourage its perpetuation; illustrations of the normal female external genitalia and the different types of mutilations were shown to demonstrate the extent of the damage inflicted by FGM; question and answer sessions were conducted after each talk session and at times formed the basis for further discussions.	Primary; Observational; Mixed methods; Pre-post design	The health education intervention had a positive impact on the attitude of respondents as there was a statistically significant increase in the number of men who wanted the practice of FGM to be stopped and a decrease in the number of respondents who intended to have their daughters mutilated in the future. A greater proportion of men than women did not want the practice of FGM stopped in the pre-intervention stage; however, there was a statistically significant decrease in the proportion of males who did not want the practice of FGM stopped in the post-intervention stage. Also, there was a statistically significant increase in the proportion of respondents who had no intention to excise future female children in the post-intervention stage.	↑	IIIb
71	*Amusan OA; Asekun-Olarinmoye EO. 2008. Knowledge, Beliefs, and Attitudes to Female Genital Mutilation (FGM) in Shao Community of Kwara State, Nigeria. International Quarterly of Community Health Education, vol. 27(4), pp. 337–349.*	Health education: intervention stage consisted of health education sessions on FGM and its complications.	Primary; Observational; Quantitative; Pre-post design	There was a statistically significant increase in the proportion of respondents who know more complications of FGM and who have no intention of circumcising future female children. Despite a high level of knowledge regarding the complications of FGM and a high level of awareness of the global campaign against it, there still exists a high prevalence of practice of FGM in Shao community, Nigeria.	→	IIIb
72	*Vogt, S et al., 2016. Changing cultural attitudes towards female genital cutting. Nature, vol. 538(7626), pp. 506–509.*	Communication – awareness creation through dramatization: designed experiments on cultural change that exploited the existence of conflicting attitudes within cutting societies. Four entertaining movies that served as experimental treatments in two experiments in Sudan. The movies were shown to people in two fully randomized and controlled experiments.	Primary; Experimental; Quantitative; Randomized Controlled Experiment	Increased debate, opened public discussion of a taboo subject, and the community energized to question the logic and arguments behind the cut and its persistence. Dramatizing discordant views on cutting within a family can improve attitudes about uncut girls. Locally heterogeneous views about cutting offer an opportunity to do this by casting the debate about cutting versus abandonment into a local vernacular.	↑	IIIa
73	*Kimani, S et al., 2018. Female Genital Mutilation/Cutting: Innovative Training Approach for Nurse-Midwives in High Prevalent Settings. Obstetrics and Gynaecology International.*	Healthcare worker training on FGM: training of nurse-midwives using an electronic tool derived from a paper-based quiz on FGM.	Primary; Observational; Quantitative; Pre-post design	Substantial improvement in FGM-related knowledge among nurse-midwives was demonstrated. However, there were challenges in preventing/ rejecting medicalization of FGM and there were knowledge gaps concerning sexual and social complications, as well as the specific roles to be played by nurse-midwives.	→	IIIb
74	*Yount, KM, Cheong, YF, Grose, RG, Hayford, SR. 2020. Community gender systems and a daughter's risk of female genital mutilation/cutting: Multilevel findings from Egypt. PLOS ONE, vol. 15(3), e0229917*	Women empowerment and gender norms: proxy measures of women's opportunities and maternal opposition to FGM.	Primary; Observational; Quantitative; cross-sectional design using demographic and health survey (DHS) data	Community gender norms opposing FGM had significant direct, negative associations with the hazard that a daughter was cut, but women's opportunities outside the family did not. Maternal opposition to FGM was negatively associated with cutting a daughter, and these associations were stronger where community opposition to FGM and opportunities for women were greater.	↑	IIIb
75	*Ruiz, IJ et al., 2016. Key points for abolishing Female Genital Mutilation from the perspective of the men involved. Midwifery, vol. 34, pp. 30–35.*	Health education and awareness campaigns: empowerment and development mechanisms via awareness raising campaigns and educational interventions.	Primary; Observational; Qualitative; Cross-sectional design	Sensitized men who have participated in awareness-raising and health education programmes can change their viewpoint regarding FGM. Awareness-raising, therefore, promotes the recognition of the complications and pathologies associated with performing FGM, and is an excellent tool for gradually eradicating FGM.	→	IV

No.	Authors/year/title/publisher	Intervention classification and description	Study type and design	Key findings	Study quality	Strength of evidence/ Gray Scale
76	*Modrek, S, and Liu JX. 2013. Exploration of pathways related to the decline in female circumcision in Egypt. BMC Public Health, vol. 13, article 921.*	Formal education: maternal educational attainment	Primary; Observational; Quantitative; Cross-sectional design using demographic and health survey (DHS) data	Positive association between education of the mother and reduced risk of daughter's undergoing FGM. Across all communities, socioeconomic status, social media messages, and women's empowerment all have significant independent effects on the risk of FGM. Additional analyses of maternal education suggest that increases in women's education may be causally related to the reduction in FGM prevalence.	→	IIIb
77	*Karlsen S et al. 2020. 'Putting salt on the wound': a qualitative study of the impact of FGM-safeguarding in healthcare settings on people with a British Somali heritage living in Bristol, UK. BMJ Open.*	Safeguarding services in healthcare: evaluation of support services to women that have undergone FGM.	Primary; Observational; Qualitative; cross-sectional design	Approaches to FGM-safeguarding have been found to directly undermine healthcare provision to FGM-affected women and families. Unnecessary, repeated, and insensitive questioning, which assume levels of dishonesty, criminality and risk, foster distrust and fear, and ultimately disengagement from health services.	↑	IV
78	*Balfour, J et al. 2016. Interventions for healthcare providers to improve treatment and prevention of female genital mutilation: a systematic review. BMC Health Services Research, vol. 16, article 409.*	Health-care provider training: interventions aimed at improving health-care providers' capacities of prevention and treatment of FGM complications.	Secondary; Systematic review. Includes two studies.	The review resulted in two studies that reported improvement of healthcare professionals' knowledge and attitude towards FGM and confidence in clinical management pre and post training. Neither evaluated the effects of the training on the quality of the care offered, the clinical outcomes of women attended, the satisfaction of the care received and prevention.	↑	IIIa
79	*Afifi, M. 2010. Egyptian ever-married women's attitude toward discontinuation of female genital cutting. Singapore Medical Journal, vol. 51(1), pp. 15–20.*	Women empowerment – maternal educational attainment: effect of education on women's attitude to discontinue FGM for their daughters.	Primary; Observational; Quantitative; cross-sectional design using demographic and health survey (DHS) data	Women with high levels of empowerment and education were more likely not to intend to perpetuate FGM for their daughters than low-empowered women and those with limited educational attainment.	→	IIIb
80	*Esu, E et al. 2017. Providing information to improve body image and care-seeking behaviour of women and girls living with female genital mutilation: A systematic review and meta-analysis. International Journal of Gynaecology and Obstetrics, vol. 136 (1), pp. 72–78.*	Women's empowerment in form of education: studies that provided education to women and/or girls living with any type of FGM or residing in countries where FGM is predominantly practiced. In all the studies, educational interventions were introduced to the intervention groups in the form of female reproductive health education, human rights, advocacy against FGM, basic hygiene, and problem solving.	Secondary; Systematic review and meta-analysis. Includes five studies with intervention and control groups.	Educational interventions provided to women living with FGM to improve body image and care-seeking behaviour were effective. Educational interventions resulted in fewer women recommending FGM for their daughters and reduced the incidence of FGM cases among daughters of women who received the educational interventions. Providing information to women and girls living with FGM appeared to result in fewer new cases of FGM reported in daughters of women who received the information. The odds of new cases of FGM were significantly lower in intervention women compared with controls.	↑	IIIa
81	*Hussein, SA, and Ghattas, S. 2019. No to circumcision: The road to effective social marketing campaigns in Egypt. Population Council, Washington DC.*	Communication/social marketing: anti-FGM communication messages through social marketing.	Primary; Mixed methods: a comparative, multi-site, mixed methods study comprising a secondary analysis of nationally representative data (the 2014 Ethiopia Demographic and Health Survey and Survey of Young People), a social media analysis of FGM content, and primary qualitative research.	Social marketing campaigns on FGM led to mothers and young women knowing the harms of FGM. Women who reported exposure to FGM information via television and radio, community education and health workers' visits were significantly more likely to support FGM abandonment than those who were not exposed to information via these channels. The association was strongest for those exposed to FGM information via community education. Prevalence of FGM among girls aged 15 to 17 years dropped from 74% to 61% from 2008 to 2014 based on a secondary analysis of DHS data.	→	IIIb

No.	Authors/year/title/publisher	Intervention classification and description	Study type and design	Key findings	Study quality	Strength of evidence/ Gray Scale
82	*UNFPA. 2017. 17 ways to end FGM/C. UNFPA*	Various interventions reviewed: religious approach, engaging communities and young people, and women's empowerment.	Secondary; other review	In Guinea, the taboo against discussing FGM publicly has been broken, and religious, community and political leaders, as well as young people, have begun to speak out against FGM. In Sudan, due to the Saleema project, being uncut is now a source of pride (over a period of just 5 years, disapproval of the practice had increased by about one-third). In Ethiopia, the meblo debates and information communicated via dagu in Afambo Woreda led to declarations of abandonment of both child marriage and FGM among some 250 communities, affecting about a quarter of a million people (133,000 male and 117,000 female). In Egypt, 65 trained community women leaders who conducted awareness-raising activities on abandonment of FGM reached parents in their respective communities. As a result, 1,080 families in Assiut have publicly declared abandonment of FGM, including 132 who had cut one daughter but vow to spare others. In Burkina Faso, there is evidence of a substantial drop in the likelihood of girls being cut since the passage of the law. The scholars estimated that legal measures have prevented nearly a quarter of a million girls and women from being cut in the past 10 years.	→	V
83	*Al-Nagar, S, Tønnessen, L, Bamkar, S. 2017. Weak law forbidding female genital mutilation in Red Sea State, Sudan. CMI.*	Legislation: criminalization of FGM in Red Sea State, a state with one of the highest prevalence rates of FGM in Sudan.	Secondary; other review	The 2011 law only addressed pharaonic circumcision, which sent a symbolic signal that Sunna circumcision was legal and legitimate. Furthermore, since the law does not describe what constitutes pharaonic circumcision, traditional midwives who customarily perform pharaonic circumcision needed only to change the label by which they call it to continue engaging in the practice. The law leaves it to the state minister of health to issue a decree forbidding FGM and does not stipulate any penalty for offenders.	↑	IV
84	*Abdulah, DM, Dawson, A, Sedo, BA. 2020. The impact of health education on attitudes of parents and religious leaders towards female genital mutilation. BMJ Sexual and Reproductive Health, vol. 46, pp. 51–58.*	Health education: 192 Mullahs (religious leaders), 212 Mokhtars (traditional leaders) and 523 parents in rural areas in Iraqi Kurdistan were invited to participate in a pre- and post-test community-based health education study. Three sessions of an FGM education intervention underpinned by the Health Belief Model (HBM) were conducted.	Primary; Observational; Quantitative; Pre-post design	The attitudes of religious leaders and parents substantially changed from a position of supporting FGM to expressing a wish to abandon the practice and not cut their future daughters.	↑	IIIb
85	*Boyden, J, Pankhurst, A, Tafere, Y. 2013. Harmful Traditional Practices and Child Protection: Contested Understandings and Practices of Female Child Marriage and Circumcision in Ethiopia. Young Lives Working Paper.*	Legislation: law enforcement and campaigns against FGM. Longitudinal intervention to tackle beliefs attitudes and knowledge on girls and women in Ethiopia.	Primary; Observational; Mixed methods: longitudinal design	Change in FGM and early marriage was influenced by participation in school, and greater economic opportunities for youth associated with modernisation. Paradoxically, especially in some of the areas where government and non-government advocates have been very active with campaigns and law enforcement, efforts have resulted in counter-reactions (unintended consequences). There has been an overall decline in both practices, with greater change in urban areas, but different regional patterns.	→	IV

No.	Authors/year/title/publisher	Intervention classification and description	Study type and design	Key findings	Study quality	Strength of evidence/ Gray Scale
86	*Ahmed, A. 2012. Evaluation of Norwegian Church Aid's (NCA) support to GBV projects implemented by SNCTP in Mayo Farm (2004–2010). Norwegian Church Aid.*	Various interventions implemented: human rights, legal, communication interventions and community change and development. The main methods used by SNCTP included community dialogues, education via participation, as well as rights-based approach, and later, psychological intervention. Presentation of visual materials such as leaflets was also used by SNCTP.	Primary; Observational; Mixed methods: cross-sectional design	The pedagogical methods used by SNCTP were appropriate to raise awareness amongst the population in the area and to enable broad accessibility for stakeholders, particularly amongst the hard-to-reach groups such as religious leaders and policy makers. An increasing number of women and men have no intention to circumcise their daughters or continue to support circumcision. Men are less likely to have preference for future partner to be circumcised.	→	IIIb
87	*Oloo, H, Wanjiru, M, Newell-Jones, K. 2011. Female genital mutilation practices in Kenya: The role of Female genital mutilation practices in Kenya: The role of alternative rites of passage. A case study of Kisii and Kuria alternative rites of passage. A case study of Kisii and Kuria districts. Population Council, Washington DC.*	Alternative Rites of Passage (ARP): community sensitization about ARP, training on family life education and public graduation ceremony.	Primary; Observational; Qualitative; cross-sectional design	ARP generally considered most appropriate for communities where FGM involves a public celebration, with the intention that the ARP graduation would, over time, replace the cut whilst retaining the traditional celebration. This would suggest that ARP would be more readily accepted among the Kuria, than among the Kisii. ARP is most effective when it takes place at the end of a structured girls empowerment programme and involves a community ceremony and is explicitly recognised as an alternative to undergoing FGM. There were some positive changes in Kisii but not in Kuria: appears that ARP in Kisii was integrated with other programmes, and is also a community affair, while in Kuria there was limited sustained community engagement, with emphasis on rescue centres hence low effect.	↑	IIIb
88	*Brown, E, and Hemmings, J. 2013. The FGM Initiative: Summary of PEER Research Endline Phase 1. Trust for London: Tackling Poverty and Inequality.*	Communication interventions and community change and development through community-based projects.	Primary; Observational; Qualitative; Participatory Ethnographic Evaluation Research (PEER) approach	Where community-based preventive work was taking place, attitudes towards FGM were changing; there was growing opposition to FGM, and growing support for a more interventionist stance to be taken by the UK government in safeguarding all women and girls from FGM. These changes are taking place against a backdrop of heightened media and policy attention on FGM. The most effective approach to FGM prevention requires multiple stakeholders at a local level, mainstreaming FGM under VAWG and/or safeguarding strategies, and community groups playing a role in prevention.	→	V
89	*Barsoum, G et al. 2011. National efforts toward FGM-free villages in Egypt: The evidence National efforts toward FGM-free villages in Egypt: The evidence of impact of impact. Population Council, Washington DC.*	Communication interventions and community change and development: through the FGM-Free Village Model is Egypt's national programme designed to eradicate the practice of FGM. The objective is to eliminate the social pressure on families to perform FGM on their daughters by fostering a sociocultural environment conducive to the abandonment of the practice through messages in the media, supportive policies, and community-based initiatives. The project was initiated in 60 villages in Upper Egypt.	Primary; Quasi-Experimental; Mixed methods (quantitative survey and qualitative: FGDs and IDIs)	More than 78% of women in the intervention group retained the information that FGM has harmful health consequences, whereas only 30% of women in the control group retained the same information. 81% of women in the intervention group stated that the information they received convinced them to re-evaluate their views about FGM, compared with only 17% of women in the control group. 76% of women in the intervention group who received information and whose daughters were uncircumcised said that the information they had received about FGM convinced them not to circumcise their daughters as compared to only 19% of women in the control sample. Only 27% of women in the intervention group believed that FGM should continue as compared to 77% of women in the control group. 25% of women in the intervention group believed that FGM was required by their religion, whereas 59% of the women in the control group held similar beliefs. Women in the control sample were more likely to believe that FGM preserves girls' virginity and protects them from being unfaithful to their husbands when they marry. Women in the intervention group were six times less likely than women in the control group to intend to circumcise their daughters in future.	→	IIIa

No.	Authors/year/title/publisher	Intervention classification and description	Study type and design	Key findings	Study quality	Strength of evidence/ Gray Scale
90	*UNICEF. 2012. Progress in abandoning female genital mutilation/cutting and child marriage in self-declared woredas in Ethiopia. Evaluation report 2012. UNICEF.*	Various interventions implemented: public declaration, legislation, health services provision and psychosocial support, training and conversion of traditional practitioners and communication interventions. The main instrument employed to bring about social change was the creation of sustained conversation on FGM complimented by teaching and/or awareness raising activities from the perspective of health, religion, and legal awareness. Involvement of religious leaders and elders, health extension workers, and law enforcement officials in the actual teaching. Utilized existing community structures to fight FGM.	Primary; Observational; Qualitative; cross-sectional design	Fewer girls were circumcised compared to adult women. FGM did not increase since the declaration of abandonment in most woredas as most respondents perceived a declining trend. There was consensus that FGM was no longer practised in the open and that it was being gradually abandoned. Nonetheless, respondents indicated that FGM had gone underground in some places especially in remote rural areas.	↑	IIIb
91	*Nielssen, H, and Coulibaly, S. 2014. The Development Program of the Region of Mopti (PDRM) Mission Evangélique Luthérienne au Mali (MELM) Final Evaluation. NORAD.*	Women's empowerment: project dedicated to the fight against FGM, accompanied by development projects in the municipality of Konna.	Primary; Observational; Qualitative; cross-sectional design	The Programme succeeded in establishing a discourse on FGM in a setting where the subject had been highly taboo. Furthermore, sensitization and training have resulted in a new awareness and knowledge of the consequences and dangers of the practice. Entire communities have now officially declared the abandonment of the practice of FGM as a consequence of the Programme. Two out of the three villages involved in the Programme had made a collective stand against FGM by signing a declaration witnessed by local authorities.	→	V
92	*Bø Nesje, FH. 2014. Effects of Schooling on Female Genital Cutting: The Case of Kenya. Master thesis, University of Oslo.*	Formal education: the effect of maternal education on the prevalence of FGM amongst their eldest daughters.	Primary; Observational; Quantitative; cross-sectional design using demographic and health survey (DHS) data	Receiving an additional year of schooling led to a 1.4 percentage points decrease in the likelihood that the eldest daughter of respondents complying with the reform was cut. The effect was causal after undertaking robust checks. Mothers with low levels of education have a higher tendency to have their daughters cut.	↑	IIIb
93	*Johansen, REB et al. 2013. What works and what does not: a discussion of popular approaches for the abandonment of female genital mutilation. Obstetrics and Gynecology International.*	Various interventions reviewed: healthcare professionals training, training and conversion of traditional practitioners, alternative rites of passage, capacity-building and institutional strengthening, communication interventions and community change and development.	Secondary; Other review. Evidence focused on six studies.	Targeting FGM is most effective and well received when a broader approach is used and the community are assisted with other challenges.	→	IIIa
94	*Boyden, J. 2012. Why are current efforts to eliminate female circumcision in Ethiopia misplaced? Culture, Health and Sexuality, vol. 14(10), pp. 1111–1123.*	Legislation: criminalization of FGM	Primary; Observational; Mixed methods: cross-sectional design	Legislative intervention resulted in the transformation, rather than the elimination, of FGM, the exchange of one type of risk for another, or even increased risk to girls. There has been a misapplication of the risk concept in the promotion of change (unintended consequences) in Ethiopia.	→	IV
95	*Nabaneh, S, and Muula, AS. 2019. Female genital mutilation/ cutting in Africa: A complex legal and ethical landscape. International Journal of Gynecology and Obstetrics, vol. 45(2).*	Legislation: Criminalization of FGM	Secondary; Other review	The fact that the practice continues despite legislative measures to protect women and girls against FGM raises the question of whether change can be legislated. Criminalization of FGM can be effective if there is a full commitment and political will within the government. In addition to enacting laws, governments must put programmes, structures, and resources in place to intensify sensitization against the practice.	→	V

No.	Authors/year/title/publisher	Intervention classification and description	Study type and design	Key findings	Study quality	Strength of evidence/ Gray Scale
96	*UNFPA–UNICEF. 2018. Performance Analysis for Phase II. UNFPA–UNICEF Joint Programme on Female Genital Mutilation: Accelerating Change. Report. UNFPA–UNICEF Joint Programme on Female Genital Mutilation.*	Various interventions implemented: legislation, trainings and capacity-building, communication/mass media/social marketing campaigns and public declarations.	Primary; Observational; Pre-post design, and secondary analysis of DHS data and other national surveys	While laws alone cannot change social norms, the adoption of criminal laws prohibiting FGM in many countries has demonstrated the positive role legislation can play in advancing the process of social change (enabling environment). Public declarations of FGM abandonment are critical, as they signal a commitment and readiness to abandon FGM. Mass and social media and other forms of communications have played a central role in amplifying public declarations and in turn encouraged other communities to abandon FGM. Community and religious leaders often have the access, power and influence to change social norms in their communities. Awareness-raising campaigns organised by youth groups in schools and universities have played a major role in changing attitudes among young people. And when boys and young men have been involved in these campaigns, change has been more likely, and harmful social norms have been tackled.	↑	IV
97	*Equality Now. 2011. Protecting Girls from Undergoing Female Genital Mutilation: The Experience of Working with the Maasai Communities in Kenya and Tanzania. Equality Now.*	Various interventions assessed: alternative rites of passage, community mobilisation and education, safe houses/rescue centres, promoting girls' education, community outreach, application of the law, empowering the youth - especially young girls, and mass education through public radio and through coalition work.	Primary; Observational; Case studies (i) The Tasaru Ntomonok Initiative (TNI) in Kenya and (ii) Network Against Female Genital Mutilation (NAFGEM) in Tanzania.	TNI's campaigns – along with other initiatives in the area – have made great contributions in the wider campaign to end FGM in the Rift Valley province. According to the KDHS survey 2008/2009, prevalence in the region has reduced from 42% in 2005 to 35% in 2009. Successful NAFGEM sensitization campaigns have empowered girls to reject the practice, with many running away or threatening to report their parents to police for prosecution.	→	IV
98	*Scott, M, de Jersey, S. 2011. Female Genital Mutilation Education Program Evaluation Final Report. West Wood Spice.*	Multiple interventions implemented: provision of education and training to health and other professionals; education and community development with affected communities; resource and information development and dissemination; and advocacy towards the prevention of FGM.	Primary; Observational; Mixed method approach-Quantitative, focus groups, face to-face, telephone and teleconference meetings.	The reach of the impact of the programme has also been international, with reported instances of changes in FGM prevalence in practising countries. Training of providers had resulted in increased knowledge and awareness of FGM related issues and confidence and capacity to respond appropriately with less judgement to women and girls from practising communities.	→	IV
99	*Gillespie, D, and Melching, M. 2010.The Transformative Power of Democracy and Human Rights in Nonformal Education: The Case of Tostan. Adult Education Quarterly.*	Human rights: Tostan's original educational approach created a meaningful context for integrating democracy and human rights into its curriculum, a process that took place from 1995 to 2003.	Primary; Observational; Mixed methods (case study) that included desk reviews (documents on Tostan) and IDIs.	The integration of human rights into the educational programme produced unexpected results, including a participant-led social movement to end harmful practices such as female genital cutting and child and/or forced marriage. The involvement of men expedited the movements to abandon harmful practices.	↑	V
100	*Johansen, REB, et al.. 2018. Health sector involvement in the management of female genital mutilation/ cutting in 30 countries. BMC Health Services Research, vol. 18, article 240.*	Health system: involvement of the health sector in the management of FGM, both in countries where FGM is a traditional practice (countries of origin), and countries where FGM is practiced mainly by migrant populations (countries of migration).	Primary; Observational; Mixed methods: cross-sectional design – quantitative data was collected in 30 countries (11 countries of origin and 19 countries of migration). Qualitative data was used to elucidate the quantitative data.	The level of the health sectors' involvement varied considerably across and within countries. Systematic training of health-care providers (HCP) was more prevalent in countries of origin, whereas involvement of HCP in the prevention of FGM was more prevalent in countries of migration.	↑	V

No.	Authors/year/title/publisher	Intervention classification and description	Study type and design	Key findings	Study quality	Strength of evidence/ Gray Scale
101	*Meroka-Mutua, AK, Mwanga, D, Olungah, CO. 2020. Assessing the role of law in reducing the practise of FGM/C in Kenya. Population Council, Washington DC.*	**Legislation: assessing the effect of legislation in six communities in Kenya, where FGM is criminalized under the 2001 Children's Act and the 2011 Prohibition of FGM Act.**	Primary; Observational; Mixed methods: cross-sectional design – KII with community gatekeepers, community leaders, and government officials; FGDs with women and men; and a quantitative survey of 1,200 respondents.	Fear of criminal sanctions acted as a motivator to obey the law but also led to FGM practised in secrecy. Conflict between custom and the law resulted in some cases in non-compliance. Poor enforcement makes it difficult to comply. 13% of the studied population would still make the decision to cut women and girls in the future.	→	IV
102	*Boyle, EH, and Corl AC. 2010. Law and culture in a global context: Interventions to eradicate female genital cutting. Annual Review of Law and Social Science, vol. 6, pp. 195–215.*	**Legislation: a review to evaluate the effects of the intersection between law, culture and context at the global level.**	Secondary; Other reviews	Change at community level in eliminating FGM is slow in the South, and faster in the North where laws are typically accompanied by enforcement.	↑	V
103	*Wouango, J, Ostermann, SL, Mwanga, D. 2020. When and How Does Law Effectively Reduce the Practice of Female Genital Mutilation/Cutting? Population Council, Washington DC*	**Legislation: in 2018, the government of Burkina Faso revised its FGM law, which is embedded in the Penal Code. In Mali, the Minister of Health, the Elderly and Solidarity instructed all the Regional Directors of Public Health and Directors of the Hospitals of Bamako and Kati, "to take all necessary measures to prevent the practice of FGM in health establishments under their moral and technical responsibility".**	Primary; Observational; Mixed methods: cross-sectional design – quantitative survey and qualitative data using KIIs and FGDs.	There was a widespread belief that FGM is illegal in both countries, even though Mali does not have a law that specifically criminalizes the practice. Progressive attitudes and behaviours in line with the international agenda, motivated by health consequences and other reasons, supported a shift towards FGM abandonment. The innovative list experiment showed that the 20 years of efforts made by Burkina Faso's government have produced results in the three villages included in the study, as the projected future prevalence rates for all villages in Burkina Faso are far lower than those of their counterparts in Mali.	↑	IV
104	*Droy, L, et al. 2018. Alternative Rites of Passage in FGMC Abandonment Campaigns in Africa: A research opportunity. University of Leicester.*	**Alternative rite of passage (ARP): The genesis of ARP in Kenya since 1996, and its significance as a hybridised cultural assemblage that forms part of new cultural and relational processes.**	Secondary; Other reviews	Not conclusive: no study has yet concretely demonstrated the positive impact of ARP interventions on the practice of FGM. The methodological limitations of existing studies mean that it is not possible to conclusively state whether (a) interventions evaluated actually generated changes in the practice of FGM (versus whether such changes would have occurred anyway); and (b) whether any changes that did occur can be attributed to the inclusion of ARP components in the interventions (as opposed to the generic community sensitization and educational activities which also occur in interventions without specific ARP components).	→	V
105	*Alkhalaileh, D, Hayford, SR, Norris, AH, Gallo, MF. 2017. Prevalence and attitudes on female genital mutilation/cutting in Egypt since criminalisation in 2008. Culture, Health and Sexuality. An International Journal for Research, Intervention and Care, vol. 20(2), pp. 173–182.*	**Legislation: criminalization of FGM law passed in Egypt in 2008.**	Primary; Observational; Quantitative; cross-sectional design using demographic and health survey (DHS) data. Analysed data from the 2005, 2008 and 2014 EDHS.	The prevalence of FGM/C among adolescent women significantly decreased, from 94% in 2008 to 88% in 2014. Prevalence of support for the continuation of FGM also significantly decreased, from 62% in 2008 to 58% in 2014. The prevalence of FGM/C among ever-married women aged 15–19 years in Egypt has decreased since its criminalization in 2008 but continues to affect the majority of this subgroup. Likewise, support for FGM continuation has also decreased but continues to be held by a majority of ever-married women of reproductive age.	↑	IIIb
106	*Ekwueme, OC, Ezegwui, HU, Ezeoke, U. 2010. Dispelling the myths and beliefs toward female genital cutting of woman: assessing general outpatient services at a tertiary health institution in Enugu state, Nigeria. East Africa Journal of Public Health, vol. 7(1), pp. 64–67.*	**Health education: education on FGM, its consequences, dispelling myths and beliefs about FGM, and knowledge to change attitudes towards FGM.**	Primary; Observational; Quantitative; Pre-post without control group design – Questionnaires were administered to a sample of 100 women drawn by systematic sampling from the General Outpatient Department of University of Nigeria Teaching Hospital.	The differences in the baseline and post-intervention data on knowledge, beliefs and attitudes of the respondents toward FGM were statistically significant.	↑	IIIb

Appendix 2. Areas for further research

- Training of health-care providers and building the capacity of the health system: how effectively can health-care providers and the health system be utilized in the prevention of FGM and the provision of services to women affected by FGM?
- Rescue centres: do rescue centres merely remove at-risk girls/women from the community, without addressing other main needs? Could rescue centres become a part of a more integrated approach to eliminating FGM?
- Range of interventions at the system and service levels: what additional interventions other than legislation can be integrated at the system level? What additional interventions other than medical services and rescue centres can be integrated at the service level?
- Translating knowledge into action: do changes in knowledge and attitudes translate into behaviour change? Further research is needed to understand how to move from changes in attitudes towards FGM to actual reductions in the number of girls and women who are cut.
- Evaluation of multi-component approaches: How can multi-component interventions involving, for example, formal education, media campaigns, legislative action, and a responsive
- Economic empowerment: how effective are economic empowerment initiatives such as gainful employment and engagement in income-generating activities in ending FGM?
- Prevention of FGM in crisis settings, e.g., COVID-19/humanitarian situations: what intervention approaches are effective in preventing FGM in crisis settings?
- Adopting a gender transformative approach to supporting the elimination of FGM: what programmes addressing gender inequalities should be considered when mainstreaming FGM?
- Engagement of men and boys: how can men and boys be allies for gender equality and for ending FGM?
- Emerging issues such as cross-border FGM: what intervention approaches are effective in preventing FGM across countries that border each other?

Note:

This list of areas for further research is not exhaustive and could be very extensive. Over the years, investment in research has increased, but there is a need for more investments in rigorous evaluations of interventions. Equally important is the need for investments in researching pathways to change and testing various theories of change models that are informed by local contexts. A prioritization process drawing from this evidence review will be central to outlining a research agenda to guide this area.

UNFPA

unicef
for every child

World Health
Organization

POPULATION
COUNCIL
KENYA
Ideas. Evidence. Impact.
