# Supplementary material for: What interventions are effective to prevent or respond to female genital mutilation? A review of existing evidence from 2008–2020
Source: PLOS Glob Public Health. 2023 May 16;3(5):e0001855. doi: 10.1371/journal.pgph.0001855 (PMC10187928; doi:10.1371/journal.pgph.0001855)
Supplement: S1 Table — (DOCX) [file pgph.0001855.s003.docx]

**RAPID EVIDENCE ASSESSMENT (REA) CHECHLIST**

| 1. Have you clearly described the background and context of the REA question | X |
| --- | --- |
| 2. Does the REA address a clearly focused question? Is it clear what the REA will answer? | X |
| 3. Have you clearly defined the inclusion criteria (e.g., population, outcomes of interest, study design)? | X |
| 4. Have you conducted a comprehensive literature search using relevant research databases (i.e., ABI/INFORM, Business Source Premier, PsycINFO, Web of Science, etc.)? | X |
| 5. Is the search systematic and reproducible (e.g., were searched information sources listed, were search terms provided, were search results reported)? | X |
| 6. Have you searched for study findings reported in unpublished or ‘grey’ literature to determine the presence of publication bias? | X |
| 7. Have you selected the studies using explicit inclusion and exclusion criteria and documented the selection process (e.g., using a flowchart to show how many studies you have excluded and why)? | X |
| 8. Did a second reviewer take a random sample from the studies included and independently review them for inclusion criteria and practical relevance? Was the inter-rater reliability assessed? | X |
| 9. Have you clearly described the key features (year of publication, population, sample size, study design, outcome measures, effect sizes, limitations, level of trustworthiness) of all studies included? | X |
| 10. Have you assessed the methodological appropriateness and methodological quality of each study using predetermined quality criteria? | X |
| 11. Did a second reviewer take a random sample from the studies included and independently review their methodological appropriateness and quality? Was the inter-rater reliability assessed? | X |
| 12. Have you provided definitions of the key elements/constructs in the REA question? | X |
| 13. Have you clearly described the assumed causal mechanism? | X |
| 14. Have you provided an overview with the main findings, including their level of trustworthiness and effect size? | X |
| 15. Have you provided an overview with the main moderators and mediators, including their level of trustworthiness and effect size? |  |
| 16. Does the synthesis describe the volume and characteristics of the overall evidence base? | X |
| 17. Does the synthesis combine the separate findings into a coherent summary of the evidence? | X |
| 18. Have you provided a clear, succinct conclusion on the main findings on the REA question? | X |
| 19. Have you clearly described all limitations and discussed how they may impact on the findings of the REA? | X |
| 20. Have you clearly described what the implications for practice are? | X |
