# Supplementary material for: What interventions are effective to prevent or respond to female genital mutilation? A review of existing evidence from 2008–2020
Source: PLOS Glob Public Health. 2023 May 16;3(5):e0001855. doi: 10.1371/journal.pgph.0001855 (PMC10187928; doi:10.1371/journal.pgph.0001855)
Supplement: S2 Table — (DOCX) [file pgph.0001855.s004.docx]

Summary of moderate- and high-quality studies

↑ high quality

→ moderate quality

↓ low quality.

| Type | Strength of evidence |
| --- | --- |
| I | Systematic review of multiple well-designed, randomized controlled trials |
| II | Well-designed, randomized controlled trial of sufficient size |
| IIIa | Well-designed trial/study without randomization that includes a control group (e.g., quasi-experimental, matched case-control studies, pre-post with control group) |
| IIIb | Well-designed trial/study without randomization that does not include a control group (e.g., single group pre-post, cohort, time series/interrupted time series, repeated cross-sectional studies) |
| IV | Well-designed, non-experimental study from more than one centre or research group, qualitative studies, and/or analysis of routine data |
| V | Opinions of respected authorities, based on clinical evidence, descriptive studies, or reports of expert committees. |

| **No.** | **Authors/year/title/publisher** | **Intervention classification and description** | **Study type and design** | **Key findings** | **Study quality** | **Strength of evidence/Gray Scale** |
| --- | --- | --- | --- | --- | --- | --- |
| 1 | Kipchumba, E, Korir, J, Abdirahman, N, Mwai, C. 2019. Accelerating Change towards Zero Tolerance to Female Genital Mutilation/Cutting: Effects of Community Dialogues on FGM/C and Child Marriage. Norwegian Church Aid and Save the Children. | Various interventions implemented. Mostly awareness creation through community conversations with different community groups, use of media, engaging religious and community leaders, engaging men and boys, capacity-building (training) of various groups and networks, advocacy on development and enactment of laws and policies. | Primary; Observational: Mixed methods (qualitative, quantitative, participatory). Panel survey was used to compare between the Mid-term and baseline findings (pre-post). Focus group discussions (FGDs) and key informant interviews (KIIs) were used to collect in-depth information to explain and support the survey. | FGM prevalence remained almost universal. Increase in medicalization in Garbaharey and Belethawa districts in Gedo, Jubaland and Bosaso, Qardo, Garowe and Eyl districts in Puntland, Somalia. Community dialogues raised awareness on negative consequences of FGM which led to a change in FGM type practised and medicalization, but not support for total abandonment. | ↑ | IIIb |
| 2 | Plugge, E, Adam, S, El Hindi, L, Gitau, J, Shodunke, N, Mohamed-Ahmed, O. 2019. The prevention of female genital mutilation in England: what can be done? *Journal of Public Health*, vol. 41(3), e261–e266. | Legislation | Primary; Observational: Qualitative. FGDs and in-depth interviews (IDIs) to understand the communities’ beliefs about how best to prevent FGM. | Legislation was counterproductive by alienating communities through its perceived imposition. Participants believed that the current UK legislation alone was not sufficient to tackle FGM. | ↑ | IV |
| 3 | Van Raemdonck A. 2019. Paradoxes of awareness raising in development: gender and sexual morality in anti-FGC campaigning in Egypt. *Culture, Health and Sexuality*, vol. 21(10), pp. 1177–1191. | Awareness creation campaign, health education through seminars/meetings | Primary; Observational: Qualitative (ethnographic).Researcher attended a series of awareness-raising seminars funded, organized and coordinated by the Coalition of NGOs against FGM. Also conducted IDIs with participants. | While the abandonment of FGM is encouraged, nationalist-modernist processes and dominant gender and sexual moralities are also reinforced. This means that certain aspects of development discourse are easily transmitted while others are subverted. Hegemonic discourses take centre stage while women’s actual lives and experiences are de-centred. | → | V |
| 4 | WHO. 2011. Female Genital Mutilation programmes to date: what works and what doesn’t. Policy brief, World Health Organization. | Various interventions reviewed: legislation, advocacy, awareness creation, capacity-building, alternative source of income for traditional practitioners, alternative rites of passage, communication, and youth involvement. | Secondary; Other review: literature review in which all anti-FGM programme documents were reviewed. Complemented with quantitative survey where a questionnaire was mailed to 365 national and international organizations. | Interventions which only supply information, education and campaigns (IEC) are not effective in changing behaviour. FGM legislation can be counterproductive. The community decision-making or consensus-building approach has significant potential for rural communities where collective decision-making is strongly valued. Using accurate media is an effective anti-FGM tool to bring about both awareness and behavioural change. Alternative income for traditional practitioners should not be the major strategy for change. | → | V |
| 5 | Population Reference Bureau. 2013. Ending female genital mutilation/cutting lessons from a decade of progress. Population Reference Bureau, Washington DC. | Various interventions reviewed: awareness campaigns, alternative incomes for traditional practitioners, alternative rites of passage, positive deviance, and safe houses. | Secondary; Other review: Desk review of evaluations studies. Systematic reviews, and donor and project reports. Supplemented with in-depth interviews with recognized experts in the field, including researchers and representatives of donor organizations. | Alternative incomes for traditional practitioners were not effective. Alternative rites of passage: success depended on local understanding and acceptance of the concept. Positive deviance: number of girls who did not undergo FGM increased during the programme. Safe houses: problematic as this approach may merely remove the girl or woman from the situation rather than address the social norms and pressures driving the practice; Legal measures: can have a negative impact in some communities, and must be complemented with comprehensive community-level interventions. | → | V |
| 6 | Njue, C, Karumbi, J, Esho, T, Varol, N, Dawson, A. 2019. Preventing female genital mutilation in high income countries: a systematic review of the evidence. *BMC Reproductive Health*, vol. 16, article 113. | Various interventions reviewed: education programmes to support affected women who may have undergone de-infibulation, legislation to protect girls, and community awareness to prevent FGM. | Secondary; Systematic review: searched databases and websites. Identified publications were screened against selection criteria, following the PRISMA guidelines. | Evidence of the effectiveness of interventions on reducing the prevalence of FGM is limited. All the evaluations measured short term outcomes, which showed improvements in knowledge but did not find any evidence on long term behaviour changes. | ↑ | IV |
| 7 | Abdi, M, and Askew, I. 2009. A religious oriented approach to addressing female genital mutilation/cutting among the Somali community of Wajir, Kenya. Population Council, Washington DC. | Awareness campaign /religious oriented approach: engaged religious scholars and educated the community about FGM with the aim of encouraging them to question why the practice is sustained and move towards abandonment. | Primary; Observational; Qualitative. | The approach led to some religious scholars and community members openly declaring their opposition to the practice, and many more privately opposing FGM. | → | V |
| 8 | Ellsberg, M, Arango, DJ, Morton, M, Gennari, F, Kiplesund, S, Contreras, M, Watts, C. 2014. Prevention of violence against women and girls: what does the evidence say? *Lancet*, vol. 385(9977), pp. 1555–1566. | Women empowerment: women-centred advocacy, group training for men and women, and economic empowerment. | Secondary; Systematic review: Reviewed evidence for interventions to reduce the prevalence and incidence of violence against women and girls. | The Tostan educational programme in Senegal led to a significant reduction in FGM among girls aged 0–10 years. Effective programmes such as Tostan are commonly participatory, engage multiple stakeholders, support critical discussion about gender relationships and the acceptability of violence, and support greater communication and shared decision making among family members. | → | IIIa |
| 9 | Cetorelli, V, Wilson, B, Batyra, E, Coast, E. 2020. Female Genital Mutilation/Cutting in Mali and Mauritania: Understanding Trends and Evaluating Policies. *Studies in Family Planning*, vol. 51(1), pp. 51–69. | Legislation: laws and policies | Primary; Quasi-experimental; Quantitative: A law banning FGM was introduced in Mauritania in 2005; in Mali, there is no legal ban on FGM. Used nationally representative survey data and a difference‐in‐difference method to evaluate the impact of the 2005 law. | The law did not have a significant impact on reducing FGM prevalence. Legislation on its own does not reduce prevalence of FGM. | ↑ | IIIa |
| 10 | Cloward, K. 2014. False Commitments: Local Misrepresentation and the International Norms Against Female Genital Mutilation and Early Marriage. *International Organization*, vol. 68(3), pp. 495–526. | Transnational activism /campaigns led by international actors. | Primary; Observational; Mixed methods: randomized field experiment and qualitative interviews. | Campaigns can lead to concealment of real behaviour and changes in attitudes. Transnational campaigns significantly influenced respondents in misrepresenting their behaviour and intentions related to FGM. | ↑ | IIIa |
| 11 | Mehari, G, Molla, A, Mamo, A, Matanda, D. 2020. Exploring changes in female genital mutilation /cutting: Shifting norms and practices among communities in Fafan and West Arsi zones, Ethiopia. Population Council, Washington DC. | Assessed the views of study respondents about the various interventions they had been exposed to: community conversation, legal intervention, religious and health-risks approaches. | Primary; Observational; Qualitative: In-depth interviews and focus group discussions. | In West Arsi: Changes in norms leading to FGM abandonment. In Fafan: no change in norms associated with FGM and no abandonment. Community conversation, legal intervention, religious and health-risks approaches can have varying impacts on different communities. | ↑ | IV |
| 12 | Boyden, J, Pankhurst, A, Tafere, Y. 2012. Child protection and harmful traditional practices: female early marriage and genital modification in Ethiopia. *Development in practice*, vol. 22(4). | Legislation - child protection laws | Primary; Observational; Longitudinal qualitative: three rounds of data gathered from a sub-sample of 50 boys and girls in 5 of the 20 Young Lives sites in Ethiopia, as well as with their peers, caregivers, and community representatives. Focus group discussions were held with boys and girls and adults. Interviews with a range of community and religious leaders, elders, and service providers. | The perceived efficacy of female early marriage and genital modification is manifested in continued resistance to reform, and unintended deleterious consequences in some cases. This suggests that there has been insufficient regard to the socio-cultural and economic context and to the rationale underlying these practices. The appropriateness and effectiveness of measures that focus on specific practices in isolation from wider social processes and relations is doubtful; there is also a need to consider reproductive health services, and measures to promote women’s education and employment. | → | IV |
| 13 | Shell-Duncan, B, Hernlund, Y, Wander, K, Moreau, A. 2013. Legislating Change? Responses to Criminalizing Female Genital Cutting in Senegal. *Law and Society Review*, vol. 47(4), pp. 803–835. | Legislation | Primary; Observational; Mixed methods: Qualitative: 98 in-depth interviews and six focus group discussions were conducted. Quantitative: multistage sampling was used and villages stratified into Tostan and non-Tostan villages. Survey data were obtained from 265 women and 82 husbands. | While some people viewed the ban as a reason to reluctantly abandon FGM, others defied the ban and continued the practice underground. Among supporters of FGM, legal norms ran counter to social norms, and did little to deter the practice, and in some instances incited reactance or drove the practice underground. Where FGM was being contested, legislation served to strengthen the stance of those contemplating or favouring abandonment. | ↑ | IIIa |
| 14 | Baillot, H, Murray, N, Connelly, E, Howard, N. 2018. Addressing female genital mutilation in Europe: a scoping review of approaches to participation, prevention, protection, and provision of services. *International Journal for Equity in Health*, vol. 17, article 21. | Various interventions reviewed: awareness creation, legislation, and preventative child protection measures. | Secondary; Other review: Scoping review of literature supplemented with qualitative interviews (16 individual and 3 group interviews). | Several countries have developed promising interventions supporting FGM prevention and recovery. Gaps remain, including community participation, professional knowledge and linkages, and evaluation of approaches. Some countries, e.g., France have led with strong criminal justice responses, while others, e.g., the Netherlands have focused on preventative child protection measures. | ↑ | IV |
| 15 | Vestbøstad, E, Blystad, A. 2014. Reflections on female circumcision discourse in Hargeysa, Somaliland: purified or mutilated? *African Journal of Reproductive Health*, vol. 18(2). | Various interventions implemented: awareness campaigns, legislation, education – FGM topic included in revised national curriculum for nursing education. | Primary; Observational; Qualitative: Qualitative interviews and informal conversations with nursing/health science teachers and office workers at the hospital (10); nursing/health science students (40); women employed in the governmental and non-governmental offices working on FGM (3); women who had lived in exile but had returned to Hargeysa (5). | Limited abandonment but more of changing the type of cut and medicalization of the practice. Open discussions about the practice and change in attitudes. The change is expressed as one going from ‘pharaoni’ (infibulation) to sunna operations, but also from sunna to an abandonment of the practice. | → | IV |
| 16 | Abathun, AD, Sundby, J, Gele, AA. 2018. Pupil's perspectives on female genital cutting abandonment in Harari and Somali regions of Ethiopia. *BMC Women's Health*, vol. 18, article 167. | Awareness creation: school based awareness campaign | Primary; Observational; Quantitative: A school-based cross-sectional study conducted in the Somali and the Harari Regional States of eastern Ethiopia. 480 respondents participated in the study. | Change in attitude in terms of support for abandonment of FGM. Participants who received information through multiple information channels were more likely to support the abandonment of FGM than those who received information from a single source. Similarly, school-based awareness campaigns and TV-based media communications were the main sources of information that influenced a high proportion of young people to support the abandonment of the practice. | ↑ | IIIa |
| 17 | Hassanin, IM, and Shaaban, OM. 2013. Impact of the complete ban on female genital cutting on the attitude of educated women from Upper Egypt toward the practice. *International Journal of Gynecology and Obstetrics*, vol. 120(3), pp. 275–278. | Legislation and formal education | Primary; Observational; Quantitative: Cross-sectional survey was carried out at the outpatient clinics of two Upper Egypt hospitals. | Small but statistically significant reduction in FGM among daughters and reduction in medicalization. Little change in attitude among educated families in Upper Egypt. The law contributed to the reduction in FGM among daughters and medicalization of the practice. | → | IIIa |
| 18 | Ako, MA, and Akweongo, P. 2009. The limited effectiveness of legislation against female genital mutilation and the role of community beliefs in Upper East Region, Ghana. *Reproductive health matters*, vol. 17(34), pp. 47–54. | Legislation | Primary; Observational: Qualitative: In-depth interviews with six state officials, a circumciser, the president of a women's advocacy organization, and semi-structured interviews with 32 community members. | While having a law criminalising FGM is necessary, it is not sufficient for elimination purposes. Although FGM has been criminalised, political support to ensure that the law is effectively implemented has been lacking. The law led to very few arrests as families protected the circumcisers. It also drove the practice underground. | ↑ | IV |
| 19 | Dowuona-Hammond, C, Atuguba, RA, Tuokuu, FXD. 2020. Women's survival in Ghana: What has law got to do with it? *SAGE Open*. | Legislation | Secondary; Other review: Desk review of existing articles, books, government reports, policy documents and parliamentary proceedings, law reports, and other documentation on women empowerment. | The law has not been effective due to traditional belief systems related to FGM, lack of awareness of the law, and economic benefits to traditional practitioners. There has been a low level of enforcement. Laws alone are insufficient to change negative practices such as FGM and advance gender equality. | → | V |
| 20 | Diop, NJ, Askew, I. 2009. The effectiveness of a community-based education program on abandoning female genital mutilation/cutting in Senegal. *Studies in Family Planning*, vol. 40(4), pp. 307–318. | Education empowerment program: TOSTAN promotes an integrated approach to learning that offers a comprehensive curriculum and improving life skills and the socioeconomic conditions of participants with a strong human rights approach. | Primary; Quasi-experimental: Pre- and post-intervention longitudinal design with a comparison group. | Significant improvements in knowledge and attitudes toward FGM among women and men who had, and had not participated in the programme, without corresponding improvement in the comparison villages. The prevalence of FGM among daughters decreased significantly over time as reported by women who were directly and indirectly exposed to the programme, but not among daughters in the comparison villages. | ↑ | IIIa |
| 21 | Rawat, R. 2017. The association between economic development, education and FGM in six selected African countries. *African Journal of Midwifery and Women's Health*, vol. 11(3). | Education and economic development | Primary; Observational; Quantitative: Used demographic and health Survey and Multiple Indicator Cluster Survey datasets (2010–2013) | Higher levels of education for women were associated with lower levels of FGM. Education was statistically significant in changing the percentage of FGM practices in the selected countries. The economic status of women was directly associated with FGM, with FGM less likely to be found among more highly educated women. | ↑ | IV |
| 22 | Kimani, S, Okondo, C. 2020. A diagnostic assessment of the health system’s response to female genital mutilation/cutting management and prevention in Kenya. Population Council, Washington DC. | Health system's response to FGM | Primary; Observational; Mixed methods: KIIs with FGM policy actors and service providers; FGDs with service providers; health facility assessments; observations of client–provider interactions, client exit interviews and service data abstraction. | The Kenyan health sector's capacity is inadequate in responding to FGM management and prevention. There were variations in awareness or knowledge of laws and policies that address FGM-related prevention and management; there was limited or inconsistent documentation of FGM-related cases and complications in the service delivery points; only minimal unstructured FGM prevention interventions were implemented; many women and girls were referred to higher-level facilities because providers lacked the capacity to address these complications; and, although women/girls were noted to present with FGM-related complications, there were no specific interventions prescribed in the guidelines and protocols to address them. | ↑ | IV |
| 23 | Van Bavel, H. 2020. At the intersection of place, gender, and ethnicity: changes in female circumcision among Kenyan Maasai. *Gender, Place & Culture*, vol. 27(8), pp. 1071–1092. | Various interventions: awareness creation, legislation, safe houses. The non-governmental organisation SAFE Maa developed an approach to challenge the social norm on FGM. The four key elements of the approach were: non-judgemental, community-led, intersectional, and showcasing wider change. | Primary; Observational; Qualitative: ethnographic approach that combines participant observation and semi-structured in-depth interviews (35). | There were changes in norms that drive FGM due to interventions implemented by the SAFE Maa project; approaches that empower people to have their own debates and make their own decisions about the future they want for their community have transformative potential. | ↑ | IV |
| 24 | Galukande, M, Kamara, J, Ndabwire, V, Leistey, E, Valla, C, Luboga, S. 2015. Eradicating female genital mutilation and cutting in Tanzania: an observational study. *BMC Public Health*, vol. 15, article 1147. | Education campaign (awareness creation): 5 school club visits; 8 community dialogue sessions; 10 training sessions on health risks of FGM involving children, local leaders and FGM practitioners; training on income generating activities; 5 training sessions involving the youth and Masai warriors; distribution of goats and chickens to ex- FGM practitioners; weekly school club activities, ex-FGM club meetings and quarterly local village advocacy subcommittee meetings; public declaration denouncing FGM and celebrating uncut girls. | Primary; Observational; Mixed methods: household survey (891 interviews); 8 KIIs (health worker, member of village health team, project coordinator, community leader, representatives from local women and men’s groups); 11 FGDs with active and ex- FGM practitioners and community members; and 4 site observation visits. Baseline and endline assessments were conducted. | Multifaceted educational campaign achieved moderate success in increasing knowledge of the health risks and changing attitudes. However, its effectiveness in reducing FGM prevalence was uncertain – the practice is currently performed secretly. | ↑ | IIIb |
| 25 | Kandala, NB, and Komba, PN. 2015. Geographic variation of female genital mutilation and legal enforcement in sub-Saharan Africa: a case study of Senegal. *The American Journal of Tropical Medicine and Hygiene*, vol. 92(4), 838–847. | Legislation | Primary; Observational: Quantitative: used the 2010–2011 Senegal Demographic Health Survey and Multiple Indicators Cluster Survey (SDHS-MICS) covering 14,228 women and their daughters. For the enforceability of the anti-FGM law, desk research was used. | The analysis showed both advantages and vulnerabilities of the anti-FGM law in relation to the issue of enforcement. The law falls short of offering adequate protection to potential victims. Data showed a zero change in prevalence in the 5 years separating the surveys. However, there were large geographic variations within the country by region. The prevalence of FGM was still high, even after Senegal signed the Maputo Protocol in 2006. | ↑ | IIIb |
| 26 | Berg, RC, and Denison, EM. 2013. A realist synthesis of controlled studies to determine the effectiveness of interventions to prevent genital cutting of girls. *Paediatrics and International Child Health*, vol. 33(4), pp. 322–333. | Various interventions reviewed: training, formal classroom education, media communication, outreach and advocacy, and informal adult education. Specific: training of health personnel, education of female students, communication programme, outreach, and advocacy, Tostan education programme. | Secondary; Realist Synthesis Approach: identified 8 effectiveness studies and 27 context studies. The review incorporated randomized controlled trials, quasi-randomized trials, controlled before-and-after studies, and interrupted time series designs on the effectiveness of interventions. To identify context factors, the study included cross-sectional quantitative studies, qualitative studies, and mixed methods studies. | The driving force for changing FGM-related behaviour was thought to be the dissemination of information. Training of health personnel: no significant difference between the intervention and comparison groups regarding any outcome; education of female students: increased students’ knowledge of the likely complications of FGM; communication programme: shift in perspective regarding FGM; Outreach and advocacy: in an Ethiopian context, it triggered an improvement in knowledge of harmful consequences of FGM, belief that it compromised the human rights of women, and intentions not to perform FGM in the future. Conversely, in a similar context involving Somali refugees, the intervention failed to generate significant change; Tostan education programme: programme resulted in negligible and small positive effects. | ↑ | IIIa |
| 27 | Van Bavel, H, Coene, G, Leye, E. 2017. Changing practices and shifting meanings of female genital cutting among the Maasai of Arusha and Manyara regions of Tanzania. *Culture, Health and Sexuality*, vol. 19(12), pp. 1344–1359. | Alternative rites of passage, formal education, and legislation. | Primary; Observational; Qualitative: IDIs with 21 women and 22 men; KIIs with 5 female and 1 male circumciser, 2 traditional Maasai leaders, 7 teachers, 2 priests, 7 heads of local women’s organizations, and 3 nurses. | Changing attitudes towards FGM among the younger generation as the result of education; the law has led to the practice being performed in secret to avoid prosecution and negative judgements by opponents. Conducting FGM in secret has caused a disconnect between the practice and the initiation ceremony. Alternative rites of passage offer those willing to continue the practice the opportunity to do so without being prosecuted, and those unwilling to undergo or perform FGM the opportunity to evade it by faking the cutting without being socially sanctioned for it. | ↑ | V |
| 28 | Evans, WD, et al. 2019. The Saleema initiative in Sudan to abandon female genital mutilation: Outcomes and dose response effects. *PLOS ONE*, vol. 14(3). | Various interventions implemented: awareness campaign, social dialogue and providing role models showing that uncut girls are socially acceptable. Specific activities included publicly pledging to abandon FGM and support the Saleema initiative, wearing Saleema colours as a sign of support, public dialogue on the existence of FGM, its role in society, and the need for abandonment, and pledges not to cut newborn daughters immediately after birth. | Primary; Quasi-experimental design: controlled for dosage of campaign messages delivered across the 18 states in Sudan to measure a dose-response effect. Social norms were operationalized through a 4-item scale. | Saleema’s social marketing strategy was effective in reducing pro-FGM social norms.  Higher levels of exposure to Saleema led to reduced pro-FGM social norms. Self-reported exposure was significantly associated with reduced pro-FGM social norms. Additionally, higher doses of Saleema, measured through an exogenous measure of campaign event exposure from an independent monitoring system, was associated with reduced pro-FGM social norms. | ↑ | IIIa |
| 29 | Denison, E, Berg, RC, Lewin, S, Fretheim, A. 2009. Effectiveness of Interventions Designed to Reduce the Prevalence of Female Genital Mutilation/Cutting. Norwegian Knowledge Centre for the Health Services. | Various interventions reviewed: education, advocacy, empowerment, and training health personnel. | Secondary; Systematic review: searched for relevant literature in scientific databases, in databases of international organizations engaged in projects concerning FGM, and in reference lists of relevant reviews and included studies. Analysis included six controlled before-and-after studies. Calculated effect estimates in outcomes for which pre- and post-scores for both intervention and comparison groups were reported. | There is a paucity of high-quality evidence regarding the effectiveness of interventions to prevent FGM and the evidence base is insufficient to draw solid conclusions.  The effect estimates suggest that (1) training health personnel likely produced no effects in knowledge or beliefs/attitudes about FGM; (2) educating female students may possibly have led to a small increase in knowledge /awareness about FGM; (3) multifaceted community activities may possibly have increased the proportion of participants having favourable cognitions and intentions about FGM; (4) community empowerment through education may possibly have positively affected prevalence of FGM, participants' knowledge about the consequences of FGM, and regrets about having had daughter cut. | ↑ | IIIa |
| 30 | Abreu, W, and Abreu, M. 2014. Community education matters: representations of female genital mutilation in Guineans immigrant women. *Procedia – Social and Behavioral Sciences*, vol. 171, pp. 620–628. | Education programme as a form of alternative ritual | Primary; Observational; Qualitative: semi-structured interviews with eight immigrant women from Guinea Bissau living in Portugal. | Educational programs emerged as an alternative to the cutting. The educational programs which involved the community and respected the local culture contributed to the reduction of FGM prevalence. | → | V |
| 31 | Ethiopian Evangelical Church Mekane Yesus Development and Social Service Commission (EECMY-DASSC/WBS-BO). 2012. Final-term Evaluation Report of Sinana Female Genital Mutilation Elimination Project (SFGMEP 2010-2012). NORAD. | Various interventions implemented: training FGM practitioners, trainers of trainers, male and female children and youths, women and men, religious leaders and project and government staff; establishing and training anti-FGM committees and clubs; organizing experience sharing visits and partners’ consultation forum; and supplying IEC/BCC materials. | Primary; Observational; Qualitative: discussion with the project management and staff; reviewed the project document as well as periodical reports of the project; discussion with the project beneficiaries – community members, Kebele administrators, religious leaders, community organisation leaders, students, anti-FGM club members, teachers, circumcisers, and teachers. | FGM practitioners rejected the practice; girls started saying no to FGM; religious leaders took initiatives and checked whether there is or no obligatory attachment of FGM in Koran and Bible; girls married without FGM; both women and men openly discussing about impacts of FGM and other harmful traditional practices which was unthinkable and a taboo before. | → | V |
| 32 | Masas, J. 2009. Evaluation Report for the Anti Female Genital Evaluation Report for the Anti Female Genital Mutilation Maasai (FGM) Project: An FPFK Advocacy programme for the Maasai of Southern Rift Region of Kenya. NORAD. | Various interventions implemented: human rights, alternative rites of passage, capacity-building and institutional strengthening, communication interventions, and community change and development. Specific: mobilize local churches to advocate for the rights of Maasai girls and women with a view to stop the practice of FGM and subsequent early and forced marriages; campaigns at the village/community level; knowledge dissemination and capacity building; and advocacy and awareness raising. | Primary; Observational; Qualitative: KIIs with staff, board members, and the project’s steering committee members; IDIs and group discussions with children; key stakeholders including chiefs, the politicians, community leaders and members, parents, cooperating agencies, church leaders and members, and schoolteachers. | Girls within the church stopped undergoing FGM; parents were knowledgeable about the disadvantages of FGM and discouraged their girls from undergoing FGM; men within the church were supportive of anti-FGM campaigns; entire church community supported the anti-FGM advocacy campaigns. Community members who still practice FGM do it in secret; and some children are taken across the border to Tanzania where they undergo FGM. | → | V |
| 33 | Nambisia, EM. 2014. Measures Influencing Eradication of Female Genital Mutilation Practices Among the Maasai Community in Maparasha Constituency Kajiado County, Kenya. University of Nairobi (UoN) Digital Repository. | Various interventions assessed: legislation, ARP, advocacy, capacity building through trainings, rescue centres, international regulatory instruments, and girl child education. | Primary; Observational; Mixed methods: a descriptive survey with a population of 666, comprising male village elders (60), women village elders (40), girls from the community who were employed (396) and community members (170). Selection of the sample from each category was done using stratified random sampling. Qualitative interviews were also conducted | Girl-child education played the most important part in FGM eradication among the Maasai. The current constitution protects children from FGM. ARP contributed to the reduction of FGM. Increased awareness on the need to eradicate FGM. The legal framework awareness is in place but implementation is still a challenge. Respondents disagreed that reconciliatory meetings between girls who escaped to rescue centres and parents was always successful and thus questioned the approach’s impact in the eradication of FGM. | → | IIIb |
| 34 | Brown, E, Porter, C. 2016. The Tackling FGM Initiative Evaluation of the Second Phase (2013–2016). Options Consultancy Services Limited. | Various interventions implemented: human rights, legal, health services provision and psychosocial support, health care professionals training, capacity-building and institutional strengthening, communication interventions, and community change and development. | Primary; Observational; Mixed methods: review of the projects’ M&E data, survey data on attitudes (people from affected communities and professionals), and KIIs. | Strong evidence from both the survey and qualitative data of an attitudinal shift towards rejecting FGM in the areas where the projects worked, as indicated by the number of respondents who did not wish FGM to continue. In a minority of cases, there was still support for FGM, particularly of forms perceived to be ‘milder’. Child protection arguments have gained traction and enabled more widespread support for ending FGM. | → | IV |
| 35 | Ugwu, I,and Ashaver, AN. 2014. TFD and community education on female genital mutilation in Igede land of Benue state: Ugengen community experience. *Creative Artist: A Journal of Theatre and Media Studies*, vol. 8(2). | Media/social marketing/ communication: the use of theatre (TFD – Theatre for Development) as a medium of communicating the consequences of FGM. The community was involved in the processes of problem analysis, drama formation, rehearsals, performance, and discussion. | Primary; Observational; Qualitative; Cross-sectional design. | People became aware of and ready to take actions on FGM. Theatre may have helped to reshape community worldview about FGM. | → | V |
| 36 | Bedri, P. 2020. Improved Understanding of FGM/C Abandonment among Sudanese Families in Khartoum and Kassala States. Sudan Working Paper, CMI, Norway. | Positive deviance – to examine the characteristics of abandoning families, their motivations, and challenges. | Primary; Observational; Qualitative: cross-sectional design. In-depth interviews were conducted. | Strong role of men in the decision to abandon FGM. FGM abandonment is a complex process, and potential for relapse still exists within families that have abandoned. | ↑ | V |
| 37 | McCracken, K. 2017. The Mayor’s Office for Policing and Crime Female Genital Mutilation Early Intervention Model: An Evaluation. Opcit Research. | Support services and community engagement: intervention targeted 3 areas of London (Tri-Borough, Tower Hamlets, and Waltham Forest), and involved prevention care and support for women that have undergone FGM as well as legal aspects. | Primary; Observational; Qualitative: cross-sectional design. | Change in attitude towards FGM among affected women; increase in specialized care and support for affected women; increased open discussion on FGM; overall improved services and service delivery. | → | IIIb |
| 38 | Cislaghi, B, et al., 2019. Changing social norms: the importance of “organized diffusion” for scaling up community health promotion and women empowerment interventions. *Prevention Science*, vol. 20(6), pp. 936–946. | Community dialogue: evidence of empowerment and norms change from three case studies – Community Empowerment Program in Mali, Change Starts at Home in Nepal, and Voices for Change in Nigeria. | Secondary; Other reviews | Community dialogue was effective in changing social norms through ‘organized diffusion’. | → | IIIb |
| 39 | Mahgoub E, et al., 2019. Effects of school-based health education on attitudes of female students towards female genital mutilation in Sudan. *Eastern Mediterranean Health Journal*, vol. 25(6), pp. 406–412. | Health education: the study included three phases; in the pre-intervention phase, data were collected from 154 students using a pre-tested questionnaire, after which students received health education sessions. The same questionnaire was used to re-collect the data in a post-intervention phase 6 weeks later. | Primary; Observational; Quantitative: Pre-post design | School-based health education has a positive impact on both knowledge and attitude of female students towards FGM in Sudan. The means of knowledge and attitude scores significantly increased comparing pre-intervention scores to post-intervention scores | ↑ | IIIa |
| 40 | Muthumbi J, et al., 2015. Female genital mutilation: a literature review of the current status of legislation and policies in 27 African countries and Yemen. *African Journal of Reproductive Health*, vol. 19(3), pp. 32–40. | Legislation: assessing the extent to which legislation, national plans of action and the integration of FGM in health frameworks have been effective in fostering the prevention of FGM and/or in accelerating its abandonment. | Secondary; Literature review that assessed the impact of Female Genital Mutilation (FGM) legislation in 28 countries (27 in Africa and Yemen) where FGM is concentrated. | Legal frameworks have had a limited impact on societal attitudes and perceptions of FGM, with evidence suggesting rigid enforcement of FGM laws has in some instances been counterproductive. Legal approaches should be complemented by measures that address the underlying socio-cultural causes that are the root of FGM. | → | V |
| 41 | Berg, RC, and Denison, E. 2012. Interventions to reduce the prevalence of female genital mutilation/cutting in African countries. *Campbell Systematic Reviews*. | Various interventions reviewed: these included reviews of studies that have assessed intervention based on human rights frameworks, legal mechanisms, health risks, alternative rites, positive deviance, training health workers as change agents, training and converting circumcisers, and the use of comprehensive social development processes. | Secondary; Systematic review. Analysis included 8 effectiveness studies and 27 context studies. | Interventions were based on a theory that dissemination of information improves cognitions about FGM, but the interventions’ success was contingent upon a range of contextual factors. With only eight controlled intervention evaluations meeting the inclusion criteria, all characterised by low methodological quality and no more than two studies synthesised in a meta-analysis for any given outcome, few firm conclusions can be drawn. Nonetheless, results point to possible advantageous developments because of FGM abandonment interventions, including lower prevalence of FGM, and changes in cognitions about FGM, such as beliefs about the practice. | ↑ | IIIa |
| 42 | Winterbottom, A, Koomen, J, Burford, G. 2009. Female genital cutting: cultural rights and rites of defiance in northern Tanzania. *African Studies Review*, vol. 52(1), pp. 47–71. | Multiple interventions assessed: health approach, ARP, and education. | Secondary; Other reviews | Campaigns against FGM using education, health, legal, and human rights–based approaches are at times ineffective and counterproductive when they frame the practice as a ‘tradition’ rooted in a ‘primitive’ and unchanging culture. | → | V |
| 43 | Berg, RC, and Denison, E. 2012. Effectiveness of interventions designed to prevent female genital mutilation/cutting: a systematic review. *Studies in Family Planning*, vol. 43(2), pp. 135–146. | Various interventions assessed: human rights frameworks, legal mechanisms, health risks, alternative rites, positive deviance, training health workers as change agents, training and converting circumcisers, and the use of comprehensive social development processes. | Secondary; Systematic review. Analysis included 8 effectiveness studies and 27 context studies. | Findings indicate that 19 of 49 outcomes were significantly different at study level, mostly favouring the intervention, but results from 4 meta-analyses showed considerable heterogeneity. The limited effectiveness and weak overall quality of the evidence from the studies appear related to methodological limitations of the studies and shortcomings in the implementation of the interventions. | ↑ | IIIa |
| 44 | Graamans, EP, et al., 2019. Lessons learned from implementing alternative rites in the fight against female genital mutilation/cutting. *The Pan African Medical Journal*, vol. 32, p. 59. | Alternative rites of passage (ARP): Amref Health Africa's efforts to end FGM/C through ARP | Primary; Observational; Qualitative; Cross-sectional design | ARP’s success hindered by lack of clarity in implementation/intervention strategy at programme level, and strongly held community perceptions including risk of exclusion; loss of cultural identity; negative stereotyping; and outsider interference. | → | V |
| 45 | Rasheed, SM, Abd-Ellah, AH, Yousef, FM. 2011. Female genital mutilation in Upper Egypt in the new millennium*. International Journal of Gynecology and Obstetrics*, vol. 114(1), pp. 47–50. | Legislation: questionnaire administered to all girls and women visiting two hospitals in Egypt, their parents and providers to establish experience, knowledge and perceptions of FGM. | Primary; Observational Quantitative; Cross-sectional design | The incidence of FGM is still very high in Upper Egypt despite the criminalization law. While general practitioners perform most procedures, most nurses are in favour of preserving the practice. In the vast majority of cases, the procedures were performed by general practitioners. | → | IV |
| 46 | Abdulah, DM, Dawson, A, Sedo, BA. 2020. The impact of health education on attitudes of parents and religious leaders towards female genital mutilation. BMJ Sexual and Reproductive Health, vol. 46, pp. 46–58. | Health education: short-term educational intervention to change the attitudes of parents and religious leaders towards FGM/ | Primary; Observational; Quantitative; Pre-post design | The attitudes of Mullahs, Mokhtars and parents substantially changed from a position of supporting female circumcision to expressing a wish to abandon the practice and not cut their future daughters. Study suggests that brief educational interventions can be an effective strategy for changing the attitudes of parents and public leaders towards FGM. | ↑ | IIIb |
| 47 | Small, E, Sharma, BB, Nikolova, SP, Tonui, BC. 2020. Hegemonic masculinity attitudes toward female genital mutilation/cutting among a sample of college students in northern and southern Sierra Leone*. Journal of Transcultural Nursing*, vol. 31(5), pp. 468–478. | Formal education: the influence of education on hegemonic masculinity attitudes between male and female students and whether parental education differentiated the groups. | Primary; Observational; Quantitative; Cross-sectional design | Formal education did not change the attitudes of students toward FGM. However, parental education had an influence on both male and female students’ attitudes toward FGM. | ↑ | IIIb |
| 48 | Matanda, D, Okondo, C, Kabiru, CW, Shell-Duncan, B. 2018. Tracing change in female genital mutilation/cutting: Shifting norms and practices among communities in Narok and Kisii counties, Kenya. Population Council, Washington DC. | Various interventions assessed: alternative rites of passage, legislation, community education. | Primary; Observational; Qualitative; Cross-sectional design | While there have been positive changes in norms and attitudes related to FGM and abandonment increasingly being adopted, the negative changes have been to make FGM secretive due to fear of prosecution, to perform FGM on younger girls in order to reduce the chance of refusal and faster healing and to medicalize the practice as a result of health education. | ↑ | IV |
| 49 | Ruiz, IJ, Martínez, PA, Giménez, LG. 2017. Eradicating Female Genital Mutilation; a viable reality. Raising awareness in the men involved. *Procedia-Social and Behavioral Sciences*. | Public declarations: eradication of FGM via the testimony of men from countries where FGM is performed. | Primary; Observational; Qualitative; Cross-sectional design | Sensitized men who have participated in awareness-raising and health education programmes can change their viewpoint regarding FGM and make declarations. There was gradual rise in the awareness of the male population regarding the problems inherent in FGM and the sexist connotations harboured therein. | → | IV |
| 50 | Afifi, M. 2009. Women's empowerment and the intention to continue the practice of female genital cutting in Egypt. *Archives of Iranian Medicine*, vol.12(2), pp. 154–160. | Women empowerment: used national representative sample of 14,393 currently married women in Egypt to measure the level of empowerment and intention to continue practising FGM. | Primary; Observational; Quantitative; Cross-section design using demographic and health survey (DHS) | Women with high levels of empowerment and education were more likely not intending to perpetuate FGM for their daughters than low empowered low-educated women. | ↑ | IIIb |
| 51 | Mwendwa, P, et al., 2020. “Promote locally led initiatives to fight female genital mutilation/cutting (FGM/C)” lessons from anti-FGM/C advocates in rural Kenya. *Reproductive Health*, vol. 17(1). | Various interventions assessed: explored the views of anti-FGM advocates on the barriers and facilitators to tackling FGM within the Meru community. | Primary; Observational; Qualitative; Cross-sectional design. | The initiatives to tackle FGM have demonstrated considerable success and as a result, the shame and disrepute that once was associated with those who had not undergone the procedure is now very much focused on those who still practice, support or enable FGM (change in attitude towards FGM). | ↑ | IV |
| 52 | Waigwa, S, Doos, L, Bradbury-Jones, C, Taylor, J. 2018. Effectiveness of health education as an intervention designed to prevent female genital mutilation/cutting (FGM/C): a systematic review. *Reproductive Health*, vol. 15(1), p. 62. | Health education: a systematic review of health education interventions to prevent FGM in Nigeria, Ghana, Kenya, Morocco, Egypt, Senegal and Ethiopia. | Secondary; Systematic review; 12 studies included in the analysis | Effectiveness of FGM health education interventions depended on factors linked to sociodemographic and socioeconomic factors; traditions and beliefs; and intervention strategy, structure and delivery. | ↑ | IIIa |
| 53 | Ameyaw, EK, et al., 2020. Female genital mutilation/cutting in Sierra Leone: are educated women intending to circumcise their daughters? *BMC International Health and Human Rights*, vol. 20, article 19. | Formal education: assessing the effect of education among women, and intention to circumcise daughters | Primary; Observational; Quantitative; Cross-sectional design using demographic and health survey (DHS) data | Women who had no formal education were more likely to intend to circumcise their daughters than those with formal education. | → | IIIb |
| 54 | Barrett, HR, et al. 2020. Transforming social norms to end FGM in the EU: an evaluation of the REPLACE Approach. *Reproductive Health*, vol. 17(40). | Community engagement: evaluated the REPLACE Approach (mainly behaviour change and community engagement intervention) through extensive engagement with eight FGM affected African diaspora communities in five EU countries. | Primary; Observational; Mixed methods; Cross-sectional design | The use of interventions that are contextually appropriate, locally generated and where the community is fully engaged are likely to be successful in changing norms and attitudes. The evaluation demonstrated that the REPLACE Approach has the potential, over time, to bring about changes in norms and attitudes associated with FGM. | → | IV |
| 55 | Johnsdotter, S. 2019. Meaning well while doing harm: compulsory genital examinations in Swedish African girls. *Sexual and Reproductive Health Matters*, vol. 27(2), pp. 87–99. | Legislation | Primary; Observational; Quantitative: a review of police records | There appears to be largely negative effects of mandatory genital examination of Swedish African girls, with psychological trauma to the girls and parents being the main (unintended) outcome. | → | V |
| 56 | Lien, IL, and Schultz, JH. 2013. Internalizing knowledge and changing attitudes to female genital cutting/ mutilation. *Obstetrics and Gynecology International*. | Health education and communication: information from different educational institutions, from seminars and conferences, from work as interpreters in hospitals, and from discussions among families and friends. | Primary; Observational; Qualitative; cross-sectional design | Most of the study participants changed their viewpoints on FGM after attending seminars, conferences, or training courses. Social and attitudinal change can happen slowly or in an instant. Not only verbal information works in an abandonment campaign, but songs, slides, and films addressing a wide variety of topics related to the procedure are also efficient tools.  that can reach the innermost level of internalization, where the motivation to change behaviour will be the strongest | → | V |
| 57 | McCracken K; et al., 2017. National FGM Centre: an evaluation. Department for Education, UK. | Service provision to FGM survivors: capacity-building of health professionals: continuum of intervention that combined work with children’s services, other statutory agencies and organizations with community outreach. | Primary; Observational; Qualitative; cross-sectional design | Improved capacity and knowledge of professionals led to increased FGM case management, improved referral pathways, as well as enhanced engagement with potentially affected communities through small scale outreaches. | ↑ | IIIb |
| 58 | UNICEF Innocenti Insight. 2010. The dynamics of social change towards: The abandonment of female genital mutilation/cutting in five African countries. The UNICEF Innocenti Research Centre. | Various interventions assessed: legislation; national policies; human rights; community conversations/engagement; and alternative rites of passage. | Primary and Secondary: Combined extensive literature reviews with qualitative and quantitative research of cross-sectional design | High effectiveness of holistic approaches in Senegal; limited effectiveness of ARP in Kenya; some success using community conversation and community dialogue in Ethiopia and Sudan. | → | IV |
| 59 | Smith, MB, and Smith, K. 2012. Final Evaluation Report. Bristol FGM Community Development Project. FORWARD. | Service provision: evaluation of support services to girls living in Bristol that have undergone FGM in order to enhance their agency and self-efficacy. | Primary; Observational; Qualitative; cross-sectional design | Changes in the confidence, knowledge, awareness and self-determination of the women who were trained to be Community Health Advocates. | → | IV |
| 60 | Ogalleh, SA. 2014. Final Evaluation of Community Education on Female Genital Mutilation (FGM) in Somaliland. International Solidarity Foundation. | Community engagement/ outreach: evaluation of Candlelight’s two phased project on community education on FGM in Somaliland. | Primary; Observational; Qualitative; cross-sectional design | The programme contributed immensely to the change of mindset and behavioural changes, i.e.. change from practising pharaonic to sunna type. The different messages by various stakeholders was confusing and challenging to sell the anti-FGM message for total abandonment. | → | V |
| 61 | Crisman, B, Dykstra, S, Kenny, C, O'Donnell M. 2016. The impact of legislation on the hazard of female genital mutilation/ cutting: regression discontinuity evidence from Burkina Faso. Center for Global Development Working Paper. | Legislation: the impact of legislation on risk of cutting in Burkina Faso. | Primary; Observational; Quantitative; Cross-sectional design using demographic and health survey (DHS) data | While the law averted FGM for approximately 237,591 girls, the results varied across regions. Legislation was accompanied by other interventions and therefore the law’s effect should be viewed with this context in mind. | ↑ | IIIb |
| 62 | Buttia, C. 2015. Investigation of successful interventions in mitigation of female genital mutilation /cutting (FGM/C) among selected Kenyan communities: Maasai, Kisii and Kuria (Master thesis*,* Hochschule für angewandte Wissenschaften Hamburg). | Various interventions reviewed: religious influence, women and girls empowerment, community education, girl education, health risk approach, exposure to other cultures, alternative rite of passage involving a reconciliation process, rescue camps, successful use of legislation, broad-based approach involving the whole community (ex-circumcisers, religious leaders, local animators and law officers) and community sensitization campaigns. | Secondary: other reviews | Pastors, priests and prophets of the three communities have managed to educate people/followers and convince parents that circumcising girls is not a requirement of the religion; education (formal and health education) has been used in the three communities to successfully reduce FGM; Alternative rites of passage has been successful though context matters; rescue centres face challenges such as limited resources and lack of its recognition especially among the Kuria; health risk education as stand-alone activity has faced the challenge of medicalization; Introducing of laws against strongly held practices such as FGM can lead to FGM being done secretly; broad-based approach involving the whole community has contributed to decrease in FGM cases. | → | V |
| 63 | Mepukori, DN. 2016. Is alternative rite of passage the key to abandonment of female genital cutting? A case study of the Samburu of Kenya. Duke University. | Alternative rite of passage (ARP): Amref programme that encourages communities to maintain the cultural ceremonies and rites surrounding female initiation whilst getting rid of FGM. | Primary; Observational; Qualitative; cross-sectional design | Sensitized community members were able to state the consequences of and oppose FGM. However, ARP training in Samburu is not universal, and the majority of the community remains largely unaware of the dangers of FGM and are unchanged in their attitudes. | ↑ | V |
| 64 | Kaunga, S. 2014. Media strategies and their influence in communicating information on Female Genital Mutilation: a case of Meru community in Tharaka District. Doctoral dissertation, University of Narobi. | Media/social marketing/ communication: the influence of media strategies in communicating information about female genital mutilation effectively among the Meru in Kenya. | Primary; Observational; Mixed methods Quantitative; cross-sectional design | Media ambassadors and the use of local language were positive and statistically significant in influencing effective communication on FGM. Conversely, the use of sheng (local slang) language and public participation were not statistically significant in influencing effective communication. | → | IIIb |
| 65 | Varol, N, et al. 2015. The role of men in abandonment of female genital mutilation: a systematic review. *BMC Public Health*, vol. 15, article 1034. | Role of men: explored men’s attitudes, beliefs, and behaviours regarding FGM, as well as their ideas about FGM prevention and abandonment. | Secondary; Systematic review | The level of education of men was the most important indicator for men’s support for abandonment of FGM. Social obligation and the lack of dialogue between men and women were acknowledged as barriers to abandonment. | ↑ | IIIa |
| 66 | Family Support Institute. 2008. Final evaluation of Tostan Community Empowerment Programme (CEP) Report. Family Support Institute. | Community engagement | Primary; Observational; Mixed methods; pre-post with a control design | Following implementation of the training programme, participants, especially women in the intervention villages, engaged in dialogue, learning, exchange of ideas, problem solving and decision making. The Tostan CEP also established functioning Community Management Committees that were transformative structures that ensured achievement of sustainable development as Tostan strengthened their capacities. This led to increased knowledge, changes in attitudes towards FGM and significant reduction of FGM prevalence in intervention villages. | → | IIIa |
| 67 | Hassanin, IMA et al., 2008. Prevalence of female genital cutting in Upper Egypt: 6 years after enforcement of prohibition law. *Reproductive BioMedicine Online*, vol. 16, pp. 27–31. | Legislation: the effect of the prohibition law of FGM in Egypt following a public outcry after a young girl died undergoing FGM in 2006. | Primary; Observational; Quantitative; Cross-sectional design | The practice of FGM in Upper Egypt remained high despite enforcement of the law. Prevalence of FGM was significantly higher in female students from rural areas as compared with urban areas. No significant differences were found in the pattern of distribution of FGM over the years from 2001 to 2006. | → | IIIb |
| 68 | Suzuki, C, and Meekers, D. 2008. Determinants of support for female genital cutting among ever-married women in Egypt. Global Public Health, vol. 3(4), pp. 383–398. | Anti-FGM communication messages: examined the effect of exposure to communication messages on support for FGM in Egypt. | Primary; Observational; Quantitative; cross-sectional design using demographic and health survey (DHS) data | There was evidence of effect of more media messages on support for discontinuing FGM among ever married women in Egypt. Women exposed to two or more FGM media messages were more likely than unexposed women to support discontinuation of FGM. | → | IIIb |
| 69 | Camilotti, G. 2016. Interventions to Stop Female Genital Cutting and the Evolution of the Custom: Evidence on Age at Cutting in Senegal. Journal of African Economies, vol. 25(1), pp. 133–158. | Legislation and awareness campaigns in Senegal: the law No. 99–05 of 29^th^ January 1999 condemns whoever is responsible for a mutilation of the female genital organs to be imprisoned for between 6 months to 5 years. | Primary; Observational; Quantitative; Cross-sectional design using demographic and health survey (DHS) data | The law has limited effect in reducing prevalence of FGM. Instead, age at cutting has reduced as girls are cut much earlier. Girls born in a year and a region where the law against FGM has been legally enforced are cut almost one year earlier. Almost 90% of the girls born before 2009 were cut before age 5. Girls born in the year and in a region where the law was enforced are cut 0.74 years earlier than girls born in another year in the same region. No statistically significant effect of the law was found on the prevalence of FGM. | ↑ | IIIb |
| 70 | Asekun-Olarinmoye, EO, and Amusan, OA. 2008. The impact of health education on attitudes towards female genital mutilation (FGM) in a rural Nigerian community. *The European Journal of Contraception and Reproductive Health Care*, vol. 13(3), pp. 289–297. | Health education (health talks): information was given in vernacular language on the female genital anatomy, the nature and the types of FGM, the complications associated with the practice, and the beliefs that encourage its perpetuation; illustrations of the normal female external genitalia and the different types of mutilations were shown to demonstrate the extent of the damage inflicted by FGM; question and answer sessions were conducted after each talk session and at times formed the basis for further discussions. | Primary; Observational; Mixed methods; Pre-post design | The health education intervention had a positive impact on the attitude of respondents as there was a statistically significant increase in the number of men who wanted the practice of FGM to be stopped and a decrease in the number of respondents who intended to have their daughters mutilated in the future. A greater proportion of men than women did not want the practice of FGM stopped in the pre-intervention stage; however, there was a statistically significant decrease in the proportion of males who did not want the practice of FGM stopped in the post-intervention stage. Also, there was a statistically significant increase in the proportion of respondents who had no intention to excise future female children in the post-intervention stage. | ↑ | IIIb |
| 71 | Amusan OA; Asekun-Olarinmoye EO. 2008. Knowledge, Beliefs, and Attitudes to Female Genital Mutilation (FGM) in Shao Community of Kwara State, Nigeria. *International Quarterly of Community Health Education*, vol. 27(4), pp. 337–349. | Health education: intervention stage consisted of health education sessions on FGM and its complications. | Primary; Observational; Quantitative; Pre-post design | There was a statistically significant increase in the proportion of respondents who know more complications of FGM and who have no intention of circumcising future female children. Despite a high level of knowledge regarding the complications of FGM and a high level of awareness of the global campaign against it, there still exists a high prevalence of practice of FGM in Shao community, Nigeria. | → | IIIb |
| 72 | Vogt, S et al., 2016. Changing cultural attitudes towards female genital cutting. *Nature*, vol. 538(7626), pp. 506–509. | Communication – awareness creation through dramatization: designed experiments on cultural change that exploited the existence of conflicting attitudes within cutting societies. Four entertaining movies that served as experimental treatments in two experiments in Sudan. The movies were shown to people in two fully randomized and controlled experiments. | Primary; Experimental; Quantitative; Randomized Controlled Experiment | Increased debate, opened public discussion of a taboo subject, and the community energized to question the logic and arguments behind the cut and its persistence. Dramatizing discordant views on cutting within a family can improve attitudes about uncut girls. Locally heterogeneous views about cutting offer an opportunity to do this by casting the debate about cutting versus abandonment into a local vernacular. | ↑ | IIIa |
| 73 | Kimani, S et al., 2018. Female Genital Mutilation/Cutting: Innovative Training Approach for Nurse-Midwives in High Prevalent Settings. *Obstetrics and Gynaecology International*. | Healthcare worker training on FGM: training of nurse-midwives using an electronic tool derived from a paper-based quiz on FGM. | Primary; Observational; Quantitative; Pre-post design | Substantial improvement in FGM-related knowledge among nurse-midwives was demonstrated. However, there were challenges in preventing/ rejecting medicalization of FGM and there were knowledge gaps concerning sexual and social complications, as well as the specific roles to be played by nurse-midwives. | → | IIIb |
| 74 | Yount, KM, Cheong, YF, Grose, RG, Hayford, SR. 2020. Community gender systems and a daughter's risk of female genital mutilation/cutting: Multilevel findings from Egypt. *PLOS ONE*, vol. 15(3), e0229917 | Women empowerment and gender norms: proxy measures of women’s opportunities and maternal opposition to FGM. | Primary; Observational; Quantitative; cross-sectional design using demographic and health survey (DHS) data | Community gender norms opposing FGM had significant direct, negative associations with the hazard that a daughter was cut, but women’s opportunities outside the family did not. Maternal opposition to FGM was negatively associated with cutting a daughter, and these associations were stronger where community opposition to FGM and opportunities for women were greater. | ↑ | IIIb |
| 75 | Ruiz, IJ et al., 2016. Key points for abolishing Female Genital Mutilation from the perspective of the men involved. *Midwifery*, vol. 34, pp. 30–35. | Health education and awareness campaigns: empowerment and development mechanisms via awareness raising campaigns and educational interventions. | Primary; Observational; Qualitative; Cross-sectional design | Sensitized men who have participated in awareness-raising and health education programmes can change their viewpoint regarding FGM. Awareness-raising, therefore, promotes the recognition of the complications and pathologies associated with performing FGM, and is an excellent tool for gradually eradicating FGM. | → | IV |
| 76 | Modrek, S, and Liu JX. 2013. Exploration of pathways related to the decline in female circumcision in Egypt. *BMC Public Health*, vol. 13, article 921. | Formal education: maternal educational attainment | Primary; Observational; Quantitative; Cross-sectional design using demographic and health survey (DHS) data | Positive association between education of the mother and reduced risk of daughter's undergoing FGM. Across all communities, socioeconomic status, social media messages, and women’s empowerment all have significant independent effects on the risk of FGM. Additional analyses of maternal education suggest that increases in women’s education may be causally related to the reduction in FGM prevalence. | → | IIIb |
| 77 | Karlsen S et al. 2020. 'Putting salt on the wound': a qualitative study of the impact of FGM-safeguarding in healthcare settings on people with a British Somali heritage living in Bristol, UK. *BMJ Open*. | Safeguarding services in healthcare: evaluation of support services to women that have undergone FGM. | Primary; Observational; Qualitative; cross-sectional design | Approaches to FGM-safeguarding have been found to directly undermine healthcare provision to FGM-affected women and families. Unnecessary, repeated, and insensitive questioning, which assume levels of dishonesty, criminality and risk, foster distrust and fear, and ultimately disengagement from health services. | ↑ | IV |
| 78 | Balfour, J et al. 2016. Interventions for healthcare providers to improve treatment and prevention of female genital mutilation: a systematic review. *BMC Health Services Research*, vol. 16, article 409. | Health-care provider training: interventions aimed at improving health-care providers’ capacities of prevention and treatment of FGM complications. | Secondary; Systematic review. Includes two studies. | The review resulted in two studies that reported improvement of healthcare professionals’ knowledge and attitude towards FGM and confidence in clinical management pre and post training. Neither evaluated the effects of the training on the quality of the care offered, the clinical outcomes of women attended, the satisfaction of the care received and prevention. | ↑ | IIIa |
| 79 | Afifi, M. 2010. Egyptian ever-married women's attitude toward discontinuation of female genital cutting. *Singapore Medical Journal*, vol. 51(1), pp. 15–20. | Women empowerment – maternal educational attainment: effect of education on women’s attitude to discontinue FGM for their daughters. | Primary; Observational; Quantitative; cross-sectional design using demographic and health survey (DHS) data | Women with high levels of empowerment and education were more likely not to intend to perpetuate FGM for their daughters than low-empowered women and those with limited educational attainment. | → | IIIb |
| 80 | Esu, E et al. 2017. Providing information to improve body image and care-seeking behaviour of women and girls living with female genital mutilation: A systematic review and meta-analysis. *International Journal of Gynaecology and Obstetrics*, vol. 136 (1), pp. 72–78. | Women’s empowerment in form of education: studies that provided education to women and/or girls living with any type of FGM or residing in countries where FGM is predominantly practiced. In all the studies, educational interventions were introduced to the intervention groups in the form of female reproductive health education, human rights, advocacy against FGM, basic hygiene, and problem solving. | Secondary; Systematic review and meta-analysis. Includes five studies with intervention and control groups. | Educational interventions provided to women living with FGM to improve body image and care-seeking behaviour were effective. Educational interventions resulted in fewer women recommending FGM for their daughters and reduced the incidence of FGM cases among daughters of women who received the educational interventions. Providing information to women and girls living with FGM appeared to result in fewer new cases of FGM reported in daughters of women who received the information. The odds of new cases of FGM were significantly lower in intervention women compared with controls. | ↑ | IIIa |
| 81 | Hussein, SA, and Ghattas, S. 2019. No to circumcision: The road to effective social marketing campaigns in Egypt. Population Council, Washington DC. | Communication/social marketing: anti-FGM communication messages through social marketing. | Primary; Mixed methods: a comparative, multi-site, mixed methods study comprising a secondary analysis of nationally representative data (the 2014 Ethiopia Demographic and Health Survey and Survey of Young People), a social media analysis of FGM content, and primary qualitative research. | Social marketing campaigns on FGM led to mothers and young women knowing the harms of FGM. Women who reported exposure to FGM information via television and radio, community education and health workers’ visits were significantly more likely to support FGM abandonment than those who were not exposed to information via these channels. The association was strongest for those exposed to FGM information via community education. Prevalence of FGM among girls aged 15 to 17 years dropped from 74% to 61% from 2008 to 2014 based on a secondary analysis of DHS data. | → | IIIb |
| 82 | UNFPA. 2017. 17 ways to end FGM/C. UNFPA | Various interventions reviewed: religious approach, engaging communities and young people, and women’s empowerment. | Secondary; other review | In Guinea, the taboo against discussing FGM publicly has been broken, and religious, community and political leaders, as well as young people, have begun to speak out against FGM. In Sudan, due to the Saleema project, being uncut is now a source of pride (over a period of just 5 years, disapproval of the practice had increased by about one-third). In Ethiopia, the meblo debates and information communicated via dagu in Afambo Woreda led to declarations of abandonment of both child marriage and FGM among some 250 communities, affecting about a quarter of a million people (133,000 male and 117,000 female). In Egypt, 65 trained community women leaders who conducted awareness-raising activities on abandonment of FGM reached parents in their respective communities. As a result, 1,080 families in Assiut have publicly declared abandonment of FGM, including 132 who had cut one daughter but vow to spare others. In Burkina Faso, there is evidence of a substantial drop in the likelihood of girls being cut since the passage of the law. The scholars estimated that legal measures have prevented nearly a quarter of a million girls and women from being cut in the past 10 years. | → | V |
| 83 | Al-Nagar, S, Tønnessen, L, Bamkar, S. 2017. Weak law forbidding female genital mutilation in Red Sea State, Sudan. CMI. | Legislation: criminalization of FGM in Red Sea State, a state with one of the highest prevalence rates of FGM in Sudan. | Secondary; other review | The 2011 law only addressed pharaonic circumcision, which sent a symbolic signal that Sunna circumcision was legal and legitimate. Furthermore, since the law does not describe what constitutes pharaonic circumcision, traditional midwives who customarily perform pharaonic circumcision needed only to change the label by which they call it to continue engaging in the practice. The law leaves it to the state minister of health to issue a decree forbidding FGM and does not stipulate any penalty for offenders. | ↑ | IV |
| 84 | Abdulah, DM, Dawson, A, Sedo, BA. 2020. The impact of health education on attitudes of parents and religious leaders towards female genital mutilation. BMJ Sexual and Reproductive Health, vol. 46, pp. 51–58. | Health education: 192 Mullahs (religious leaders), 212 Mokhtars (traditional leaders) and 523 parents in rural areas in Iraqi Kurdistan were invited to participate in a pre- and post-test community-based health education study. Three sessions of an FGM education intervention underpinned by the Health Belief Model (HBM) were conducted. | Primary; Observational; Quantitative; Pre-post design | The attitudes of religious leaders and parents substantially changed from a position of supporting FGM to expressing a wish to abandon the practice and not cut their future daughters. | ↑ | IIIb |
| 85 | Boyden, J, Pankhurst, A, Tafere, Y. 2013. Harmful Traditional Practices and Child Protection: Contested Understandings and Practices of Female Child Marriage and Circumcision in Ethiopia. Young Lives Working Paper. | Legislation: law enforcement and campaigns against FGM. Longitudinal intervention to tackle beliefs attitudes and knowledge on girls and women in Ethiopia. | Primary; Observational; Mixed methods: longitudinal design | Change in FGM and early marriage was influenced by participation in school, and greater economic opportunities for youth associated with modernisation. Paradoxically, especially in some of the areas where government and non-government advocates have been very active with campaigns and law enforcement, efforts have resulted in counter-reactions (unintended consequences). There has been an overall decline in both practices, with greater change in urban areas, but different regional patterns. | → | IV |
| 86 | Ahmed, A. 2012. Evaluation of Norwegian Church Aid’s (NCA) support to GBV projects implemented by SNCTP in Mayo Farm (2004–2010). Norwegian Church Aid. | Various interventions implemented: human rights, legal, communication interventions and community change and development. The main methods used by SNCTP included community dialogues, education via participation, as well as rights-based approach, and later, psychological intervention. Presentation of visual materials such as leaflets was also used by SNCTP. | Primary; Observational; Mixed methods: cross-sectional design | The pedagogical methods used by SNCTP were appropriate to raise awareness amongst the population in the area and to enable broad accessibility for stakeholders, particularly amongst the hard-to-reach groups such as religious leaders and policy makers. An increasing number of women and men have no intention to circumcise their daughters or continue to support circumcision. Men are less likely to have preference for future partner to be circumcised. | → | IIIb |
| 87 | Oloo, H, Wanjiru, M, Newell-Jones, K. 2011. Female genital mutilation practices in Kenya: The role of Female genital mutilation practices in Kenya: The role of alternative rites of passage. A case study of Kisii and Kuria alternative rites of passage. A case study of Kisii and Kuria districts. Population Council, Washington DC. | Alternative Rites of Passage (ARP): community sensitization about ARP, training on family life education and public graduation ceremony. | Primary; Observational; Qualitative; cross-sectional design | ARP generally considered most appropriate for communities where FGM involves a public celebration, with the intention that the ARP graduation would, over time, replace the cut whilst retaining the traditional celebration. This would suggest that ARP would be more readily accepted among the Kuria, than among the Kisii. ARP is most effective when it takes place at the end of a structured girls empowerment programme and involves a community ceremony and is explicitly recognised as an alternative to undergoing FGM. There were some positive changes in Kisii but not in Kuria: appears that ARP in Kisii was integrated with other programmes, and is also a community affair, while in Kuria there was limited sustained community engagement, with emphasis on rescue centres hence low effect. | ↑ | IIIb |
| 88 | Brown, E, and Hemmings, J. 2013. The FGM Initiative: Summary of PEER Research Endline Phase 1. Trust for London: Tackling Poverty and Inequality. | Communication interventions and community change and development through community-based projects. | Primary; Observational; Qualitative; Participatory Ethnographic Evaluation Research (PEER) approach | Where community-based preventive work was taking place, attitudes towards FGM were changing; there was growing opposition to FGM, and growing support for a more interventionist stance to be taken by the UK government in safeguarding all women and girls from FGM. These changes are taking place against a backdrop of heightened media and policy attention on FGM. The most effective approach to FGM prevention requires multiple stakeholders at a local level, mainstreaming FGM under VAWG and/or safeguarding strategies, and community groups playing a role in prevention. | → | V |
| 89 | Barsoum, G et al. 2011. National efforts toward FGM-free villages in Egypt: The evidence National efforts toward FGM-free villages in Egypt: The evidence of impact of impact. Population Council, Washington DC. | Communication interventions and community change and development: through the FGM-Free Village Model is Egypt’s national programme designed to eradicate the practice of FGM. The objective is to eliminate the social pressure on families to perform FGM on their daughters by fostering a sociocultural environment conducive to the abandonment of the practice through messages in the media, supportive policies, and community-based initiatives. The project was initiated in 60 villages in Upper Egypt. | Primary; Quasi-Experimental; Mixed methods (quantitative survey and qualitative: FGDs and IDIs) | More than 78% of women in the intervention group retained the information that FGM has harmful health consequences, whereas only 30% of women in the control group retained the same information. 81% of women in the intervention group stated that the information they received convinced them to re-evaluate their views about FGM, compared with only 17% of women in the control group. 76% of women in the intervention group who received information and whose daughters were uncircumcised said that the information they had received about FGM convinced them not to circumcise their daughters as compared to only 19% of women in the control sample. Only 27% of women in the intervention group believed that FGM should continue as compared to 77% of women in the control group. 25% of women in the intervention group believed that FGM was required by their religion, whereas 59% of the women in the control group held similar beliefs. Women in the control sample were more likely to believe that FGM preserves girls’ virginity and protects them from being unfaithful to their husbands when they marry. Women in the intervention group were six times less likely than women in the control group to intend to circumcise their daughters in future. | → | IIIa |
| 90 | UNICEF. 2012. Progress in abandoning female genital mutilation/cutting and child marriage in self-declared woredas in Ethiopia. Evaluation report 2012. UNICEF. | Various interventions implemented: public declaration, legislation, health services provision and psychosocial support, training and conversion of traditional practitioners and communication interventions. The main instrument employed to bring about social change was the creation of sustained conversation on FGM complimented by teaching and/or awareness raising activities from the perspective of health, religion, and legal awareness. Involvement of religious leaders and elders, health extension workers, and law enforcement officials in the actual teaching. Utilized existing community structures to fight FGM. | Primary; Observational; Qualitative; cross-sectional design | Fewer girls were circumcised compared to adult women. FGM did not increase since the declaration of abandonment in most woredas as most respondents perceived a declining trend. There was consensus that FGM was no longer practised in the open and that it was being gradually abandoned. Nonetheless, respondents indicated that FGM had gone underground in some places especially in remote rural areas. | ↑ | IIIb |
| 91 | Nielssen, H, and Coulibaly, S. 2014. The Development Program of the Region of Mopti (PDRM) Mission Evangélique Luthérienne au Mali (MELM) Final Evaluation. NORAD. | Women’s empowerment: project dedicated to the fight against FGM, accompanied by development projects in the municipality of Konna. | Primary; Observational; Qualitative; cross-sectional design | The Programme succeeded in establishing a discourse on FGM in a setting where the subject had been highly taboo. Furthermore, sensitization and training have resulted in a new awareness and knowledge of the consequences and dangers of the practice. Entire communities have now officially declared the abandonment of the practice of FGM as a consequence of the Programme. Two out of the three villages involved in the Programme had made a collective stand against FGM by signing a declaration witnessed by local authorities. | → | V |
| 92 | Bø Nesje, FH. 2014. Effects of Schooling on Female Genital Cutting: The Case of Kenya. Master thesis, University of Oslo. | Formal education: the effect of maternal education on the prevalence of FGM amongst their eldest daughters. | Primary; Observational; Quantitative; cross-sectional design using demographic and health survey (DHS) data | Receiving an additional year of schooling led to a 1.4 percentage points decrease in the likelihood that the eldest daughter of respondents complying with the reform was cut. The effect was causal after undertaking robust checks. Mothers with low levels of education have a higher tendency to have their daughters cut. | ↑ | IIIb |
| 93 | Johansen, REB et al. 2013. What works and what does not: a discussion of popular approaches for the abandonment of female genital mutilation. *Obstetrics and Gynecology International*. | Various interventions reviewed: health-care professionals training, training and conversion of traditional practitioners, alternative rites of passage, capacity-building and institutional strengthening, communication interventions and community change and development. | Secondary; Other review. Evidence focused on six studies. | Targeting FGM is most effective and well received when a broader approach is used and the community are assisted with other challenges. | → | IIIa |
| 94 | Boyden, J. 2012. Why are current efforts to eliminate female circumcision in Ethiopia misplaced? *Culture, Health and Sexuality*, vol. 14(10), pp. 1111–1123. | Legislation: criminalization of FGM | Primary; Observational; Mixed methods: cross-sectional design | Legislative intervention resulted in the transformation, rather than the elimination, of FGM, the exchange of one type of risk for another, or even increased risk to girls. There has been a misapplication of the risk concept in the promotion of change (unintended consequences) in Ethiopia. | → | IV |
| 95 | Nabaneh, S, and Muula, AS. 2019. Female genital mutilation/ cutting in Africa: A complex legal and ethical landscape. *International Journal of Gynecology and Obstetrics*, vol. 45(2). | Legislation: Criminalization of FGM | Secondary; Other review | The fact that the practice continues despite legislative measures to protect women and girls against FGM raises the question of whether change can be legislated. Criminalization of FGM can be effective if there is a full commitment and political will within the government. In addition to enacting laws, governments must put programmes, structures, and resources in place to intensify sensitization against the practice. | → | V |
| 96 | UNFPA–UNICEF. 2018. Performance Analysis for Phase II. UNFPA–UNICEF Joint Programme on Female Genital Mutilation: Accelerating Change. Report. UNFPA–UNICEF Joint Programme on Female Genital Mutilation. | Various interventions implemented: legislation, trainings and capacity-building, communication/mass media/social marketing campaigns and public declarations. | Primary; Observational; Pre-post design, and secondary analysis of DHS data and other national surveys | While laws alone cannot change social norms, the adoption of criminal laws prohibiting FGM in many countries has demonstrated the positive role legislation can play in advancing the process of social change (enabling environment). Public declarations of FGM abandonment are critical, as they signal a commitment and readiness to abandon FGM. Mass and social media and other forms of communications have played a central role in amplifying public declarations and in turn encouraged other communities to abandon FGM. Community and religious leaders often have the access, power and influence to change social norms in their communities. Awareness-raising campaigns organised by youth groups in schools and universities have played a major role in changing attitudes among young people. And when boys and young men have been involved in these campaigns, change has been more likely, and harmful social norms have been tackled. | ↑ | IV |
| 97 | Equality Now. 2011. Protecting Girls from Undergoing Female Genital Mutilation: The Experience of Working with the Maasai Communities in Kenya and Tanzania. Equality Now. | Various interventions assessed: alternative rites of passage, community mobilisation and education, safe houses/rescue centres, promoting girls’ education, community outreach, application of the law, empowering the youth - especially young girls, and mass education through public radio and through coalition work. | Primary; Observational; Case studies (i) The Tasaru Ntomonok Initiative (TNI) in Kenya and (ii) Network Against Female Genital Mutilation (NAFGEM) in Tanzania. | TNI’s campaigns – along with other initiatives in the area – have made great contributions in the wider campaign to end FGM in the Rift Valley province. According to the KDHS survey 2008/2009, prevalence in the region has reduced from 42% in 2005 to 35% in 2009. Successful NAFGEM sensitization campaigns have empowered girls to reject the practice, with many running away or threatening to report their parents to police for prosecution. | **→** | IV |
| 98 | Scott, M, de Jersey, S. 2011. Female Genital Mutilation Education Program Evaluation Final Report. West Wood Spice. | Multiple interventions implemented: provision of education and training to health and other professionals; education and community development with affected communities; resource and information development and dissemination; and advocacy towards the prevention of FGM. | Primary; Observational; Mixed method approach-Quantitative, focus groups, face to-face, telephone and teleconference meetings. | The reach of the impact of the programme has also been international, with reported instances of changes in FGM prevalence in practising countries. Training of providers had resulted in increased knowledge and awareness of FGM related issues and confidence and capacity to respond appropriately with less judgement to women and girls from practising communities. | **→** | IV |
| 99 | Gillespie, D, and Melching, M. 2010.The Transformative Power of Democracy and Human Rights in Nonformal Education: The Case of Tostan. *Adult Education Quarterly*. | Human rights: Tostan’s original educational approach created a meaningful context for integrating democracy and human rights into its curriculum, a process that took place from 1995 to 2003. | Primary; Observational; Mixed methods (case study) that included desk reviews (documents on Tostan) and IDIs. | The integration of human rights into the educational programme produced unexpected results, including a participant-led social movement to end harmful practices such as female genital cutting and child and/or forced marriage. The involvement of men expedited the movements to abandon harmful practices. | ↑ | V |
| 100 | Johansen, REB, et al.. 2018. Health sector involvement in the management of female genital mutilation/ cutting in 30 countries. *BMC Health Services Research*, vol. 18, article 240. | Health system: involvement of the health sector in the management of FGM, both in countries where FGM is a traditional practice (countries of origin), and countries where FGM is practiced mainly by migrant populations (countries of migration). | Primary; Observational; Mixed methods: cross-sectional design – quantitative data was collected in 30 countries (11 countries of origin and 19 countries of migration). Qualitative data was used to elucidate the quantitative data. | The level of the health sectors’ involvement varied considerably across and within countries. Systematic training of health-care providers (HCP) was more prevalent in countries of origin, whereas involvement of HCP in the prevention of FGM was more prevalent in countries of migration. | ↑ | V |
| 101 | Meroka-Mutua, AK, Mwanga, D, Olungah, CO. 2020. Assessing the role of law in reducing the practise of FGM/C in Kenya. Population Council, Washington DC. | Legislation: assessing the effect of legislation in six communities in Kenya, where FGM is criminalized under the 2001 Children’s Act and the 2011 Prohibition of FGM Act. | Primary; Observational; Mixed methods: cross-sectional design – KII with community gatekeepers, community leaders, and government officials; FGDs with women and men; and a quantitative survey of 1,200 respondents. | Fear of criminal sanctions acted as a motivator to obey the law but also led to FGM practised in secrecy. Conflict between custom and the law resulted in some cases in non-compliance. Poor enforcement makes it difficult to comply. 13% of the studied population would still make the decision to cut women and girls in the future. | → | IV |
| 102 | Boyle, EH, and Corl AC. 2010. Law and culture in a global context: Interventions to eradicate female genital cutting. Annual Review of Law and Social Science, vol. 6, pp. 195–215. | Legislation: a review to evaluate the effects of the intersection between law, culture and context at the global level. | Secondary; Other reviews | Change at community level in eliminating FGM is slow in the South, and faster in the North where laws are typically accompanied by enforcement. | ↑ | V |
| 103 | Wouango, J, Ostermann, SL, Mwanga, D. 2020. When and How Does Law Effectively Reduce the Practice of Female Genital Mutilation/Cutting? Population Council, Washington DC | Legislation: in 2018, the government of Burkina Faso revised its FGM law, which is embedded in the Penal Code. In Mali, the Minister of Health, the Elderly and Solidarity instructed all the Regional Directors of Public Health and Directors of the Hospitals of Bamako and Kati, "to take all necessary measures to prevent the practice of FGM in health establishments under their moral and technical responsibility". | Primary; Observational; Mixed methods: cross-sectional design – quantitative survey and qualitative data using KIIs and FGDs. | There was a widespread belief that FGM is illegal in both countries, even though Mali does not have a law that specifically criminalizes the practice. Progressive attitudes and behaviours in line with the international agenda, motivated by health consequences and other reasons, supported a shift towards FGM abandonment. The innovative list experiment showed that the 20 years of efforts made by Burkina Faso’s government have produced results in the three villages included in the study, as the projected future prevalence rates for all villages in Burkina Faso are far lower than those of their counterparts in Mali. | ↑ | IV |
| 104 | Droy, L, et al. 2018. Alternative Rites of Passage in FGMC Abandonment Campaigns in Africa: A research opportunity. University of Leicester. | Alternative rite of passage (ARP): The genesis of ARP in Kenya since 1996, and its significance as a hybridised cultural assemblage that forms part of new cultural and relational processes. | Secondary; Other reviews | Not conclusive: no study has yet concretely demonstrated the positive impact of ARP interventions on the practice of FGM. The methodological limitations of existing studies mean that it is not possible to conclusively state whether (a) interventions evaluated actually generated changes in the practice of FGM (versus whether such changes would have occurred anyway); and (b) whether any changes that did occur can be attributed to the inclusion of ARP components in the interventions (as opposed to the generic community sensitization and educational activities which also occur in interventions without specific ARP components). | **→** | V |
| 105 | Alkhalaileh, D, Hayford, SR, Norris, AH, Gallo, MF. 2017. Prevalence and attitudes on female genital mutilation/cutting in Egypt since criminalisation in 2008. *Culture, Health and Sexuality. An International Journal for Research, Intervention and Care*, vol. 20(2), pp. 173–182. | Legislation: criminalization of FGM law passed in Egypt in 2008. | Primary; Observational; Quantitative; cross-sectional design using demographic and health survey (DHS) data. Analysed data from the 2005, 2008 and 2014 EDHS. | The prevalence of FGM/C among adolescent women significantly decreased, from 94% in 2008 to 88% in 2014. Prevalence of support for the continuation of FGM also significantly decreased, from 62% in 2008 to 58% in 2014. The prevalence of FGM/C among ever-married women aged 15–19 years in Egypt has decreased since its criminalization in 2008 but continues to affect the majority of this subgroup. Likewise, support for FGM continuation has also decreased but continues to be held by a majority of ever-married women of reproductive age. | ↑ | IIIb |
| 106 | Ekwueme, OC, Ezegwui, HU, Ezeoke, U. 2010. Dispelling the myths and beliefs toward female genital cutting of woman: assessing general outpatient services at a tertiary health institution in Enugu state, Nigeria. *East Africa Journal of Public Health*, vol. 7(1), pp. 64–67. | Health education: education on FGM, its consequences, dispelling myths and beliefs about FGM, and knowledge to change attitudes towards FGM. | Primary; Observational; Quantitative; Pre-post without control group design – Questionnaires were administered to a sample of 100 women drawn by systematic sampling from the General Outpatient Department of University of Nigeria Teaching Hospital. | The differences in the baseline and post-intervention data on knowledge, beliefs and attitudes of the respondents toward FGM were statistically significant. | ↑ | IIIb |
